# Algorithms for the automated correction of vertical drift in eye-tracking data

**DOI:** 10.3758/s13428-021-01554-0

**Published:** 2021-06-22

**Authors:** Jon W. Carr, Valentina N. Pescuma, Michele Furlan, Maria Ktori, Davide Crepaldi

**Affiliations:** International School for Advanced Studies (SISSA), Via Bonomea 265, 34136 Trieste, TS Italy

**Keywords:** Algorithms, Dynamic time warping, Eye tracking, Line assignment, Reading, Vertical drift

## Abstract

A common problem in eye-tracking research is vertical drift—the progressive displacement of fixation registrations on the vertical axis that results from a gradual loss of eye-tracker calibration over time. This is particularly problematic in experiments that involve the reading of multiline passages, where it is critical that fixations on one line are not erroneously recorded on an adjacent line. Correction is often performed manually by the researcher, but this process is tedious, time-consuming, and prone to error and inconsistency. Various methods have previously been proposed for the automated, post hoc correction of vertical drift in reading data, but these methods vary greatly, not just in terms of the algorithmic principles on which they are based, but also in terms of their availability, documentation, implementation languages, and so forth. Furthermore, these methods have largely been developed in isolation with little attempt to systematically evaluate them, meaning that drift correction techniques are moving forward blindly. We document ten major algorithms, including two that are novel to this paper, and evaluate them using both simulated and natural eye-tracking data. Our results suggest that a method based on dynamic time warping offers great promise, but we also find that some algorithms are better suited than others to particular types of drift phenomena and reading behavior, allowing us to offer evidence-based advice on algorithm selection.

**Supplementary Information:**

The online version contains supplementary material available at 10.3758/s13428-021-01554-0.

## Introduction

Reading is a fundamental skill for navigating modern society and, as such, is subject to intense study in the cognitive and language sciences. Among the many tools that researchers use to investigate reading in the laboratory, eye tracking occupies a prominent position. Using this technique, participants’ eye movements may be recorded as they read written material, providing a window into the relevant cognitive processes as they unfold. Technological advancements in eye tracking, particularly from the 1970s (Rayner, 1998), have allowed researchers to collect increasingly accurate measures of eye movements during reading tasks, leading to great improvements in the investigation of the cognitive processes underlying reading and reading acquisition.

Many eye-tracking studies involve the reading of single words or sentences. For example, researchers may embed target words into different sentence contexts and manipulate predictability (e.g., Rayner et al.,, 2001), display isolated words to gain insight into how a reader’s eye moves when processing a word (e.g., Vitu et al.,, 2004), or reveal parts of words in a gaze-contingent fashion to investigate parafoveal processing (e.g., Schotter et al.,, 2012). Sentence reading experiments have also been essential in revealing the cognitive processes behind different levels of written language processing, from the width of the perceptual span (e.g., Blythe et al.,, 2009; Rayner, 1986) to the effects that word length and frequency have on eye movements (e.g., Joseph et al.,, 2009; Tiffin-Richards & Schroeder, 2015), as well as the effects of syntactic (e.g., Frazier & Rayner, 1982; Pickering & Traxler, 1998) and lexical (e.g., Sereno et al.,, 2006) ambiguity.

In our everyday experience, however, we often do not encounter sentences in isolation; a good part of our reading experience involves connected text that is distributed over multiple lines. Therefore, experiments based on paragraph reading also provide insight into the reading experience, while allowing us to address levels of processing that are simply not available when one reads a single sentence, such as the role of broader context or the integration of syntactic relations across sentence boundaries (Jarodzka and Brand-Gruwel, 2017). Indeed, studies of multiline reading have become more prevalent in recent years, with researchers using passage reading tasks to investigate, for example, the effect of text- and participant-level characteristics on eye movements (Kuperman et al., 2018) or of contextual facilitation on developing readers’ eye movements (Tiffin-Richards & Schroeder, 2020). Several multiline-reading datasets have also been released, including GECO (Cop et al., 2017), MECO (Kuperman et al., under review), and Provo (Luke & Christianson, 2018).

**Fig. 1:**
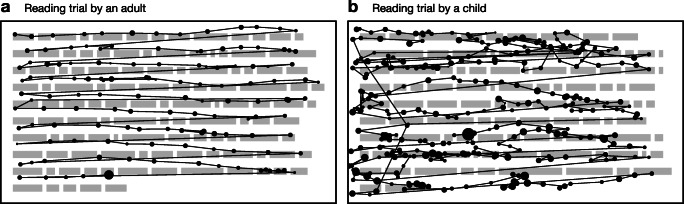
Example reading trials from an adult participant (*left*) and a child participant (*right*) taken from Pescuma et al., (in prep). Each *dot* represents a fixation and the size of the dots represents duration. The adult trial exhibits upward shift, especially in the lower left part of the passage. The child trial is extremely noisy and exhibits not just vertical drift issues but also many natural reading phenomena that will pose challenges to the algorithms

A technical issue that arises from the particular circumstances of multiline reading is so-called “vertical drift,” which we define as the progressive displacement of fixation registrations on the vertical axis *over time*. In other words, fixations may be recorded above or below the line of text that the participant was actually reading, and the degree and directionality of this error may fluctuate dynamically with each subsequent fixation, making it nontrivial to eliminate. Figure 1a depicts a reading trial exhibiting vertical drift phenomena; in this case, fixations—especially those on the left-hand side—are recorded around one line higher than where the reader was actually fixating, but they also tend to slope down to the right such that fixations on the right-hand side seem to be better aligned.

Vertical drift can occur quite unpredictably, even following good-quality calibration, and it is likely caused by spatial phenomena such as degraded eye-tracker calibration at the corners of the screen, or temporal phenomena such as subtle movements in head position or pupil dilation, which can be difficult to control for, even in a laboratory setting. Such sources of measurement error are often exacerbated in the context of multiline reading because, in comparison to single words or sentences, passages of text are distributed over a larger portion of the screen, including areas where general calibration may be worse, and they take longer to read, during which time calibration may begin to degrade. There are also less frequent opportunities to recalibrate the device during passage reading, since this can only be performed between trials or pages and not during the reading of a passage.

Whatever the cause, and however it manifests itself, vertical drift will ultimately have a negative impact on the analysis of eye-tracking data because fixations will be mapped to words that were not actually being fixated at a given point in time (as we can see in Fig. 1a). It is therefore incumbent on the researcher to recognize such issues when they occur and to take corrective measures. Specialized software packages, such as EyeLink Data Viewer (SR Research, Toronto, Canada) or EyeDoctor (UMass Eyetracking Lab, Amherst, MA, USA), provide the ability to manually move fixations, either individually or in small batches. However, manual realignment can be very time-consuming and is likely to be error prone. In particular, the realignment process can be greatly complicated by other sources of noise or idiosyncratic reading behaviors. For example, Fig. 1b depicts a reading trial by a child reader; in this case, not only are the fixations affected by drift issues, but there are also various natural reading behaviors, such as within- and between-line regressions, which add an additional layer of complexity to the task of realignment, not to mention the baseline level of noise and unusual features such as the arching sequence of fixations targeting line 4.

A number of methods have previously been developed to automate post hoc vertical drift correction. FixAlign, an R package developed by Cohen (2013), is currently the most well-established method in the experimental psychology community, although other methods have recently been proposed by Schroeder (2019) and Špakov et al., (2019). In addition, there is a disparate body of work from several subfields of computer science, such as biometrics (Abdulin and Komogortsev, 2015), educational technology (Hyrskykari, 2006), and user-interface design (Beymer & Russell, 2005), in which various ad-hoc algorithms have been reported (see also Carl, 2013; Lima Sanches et al.,, 2015; Lohmeier, 2015; Martinez-Gomez et al.,, 2012; Mishra et al.,, 2012; Nüssli, 2011; Palmer & Sharif, 2016; Sibert et al.,, 2000; Yamaya et al.,, 2017).

These reported methods can be difficult to evaluate and use because they vary widely in terms of their availability, design choices, implementation languages, usability, level of documentation, expected input data, and the extent to which they rely on project-specific heuristics or particular eye-tracker hardware. Furthermore, these methods have largely been developed in isolation from each other, and there has been little attempt to systematically evaluate them, so drift correction software is moving forward blindly without an evidence base to support new directions. In this paper, we attempt to classify the reported methods into ten major approaches, which we formalize as ten simple algorithms that adopt a consistent design model. In other words, we do not attempt to evaluate existing software implementations; rather, we explore the spectrum of drift correction algorithms by isolating and evaluating the core principles on which previous methods have been based. Our goal is to provide a systematic comparison of these algorithms in order to guide researchers’ choices about the most suitable methods and to lay a solid foundation for future drift correction software.

To be clear, the algorithms we consider in this paper are restricted to one specific problem. Firstly, we only consider algorithms designed for the ordinary reading of passages of text; other uses of eye tracking, such as visual search and scene perception, can also undergo drift correction, but the methods required are quite different (see, e.g., Vadillo et al.,, 2015; Zhang & Hornof, 2011, 2014). Similarly, the reading of source code has received some attention, but the affordances and constraints in this domain are quite different from ordinary linguistic reading (see, e.g., Nüssli 2011; Palmer & Sharif, 2016). Secondly, we only consider the problem of post hoc correction; vertical drift can also be corrected in real time, but this imposes a more restrictive set of constraints that are better handled by other types of algorithm (see, e.g., Hyrskykari, 2006; Sibert et al.,, 2000). Thirdly, we only consider fully automated algorithms that do not require human supervision.

The paper proceeds in four main sections. First, we outline the algorithms. Second, we test the algorithms on simulated fixation sequences afflicted with various types of measurement error. Third, we test the algorithms on an eye-tracking dataset (two examples from which are presented in Fig. 1). Finally, we discuss the major properties of the algorithms, provide guidance to researchers about their use, and suggest ways in which they can be improved further. All code and data required to reproduce the analyses reported in this paper, as well as Matlab/Octave, Python, and R implementations of the algorithms, are available from the public data archive associated with this paper: 10.17605/OSF.IO/7SRKG

## Algorithms

In this section, we describe ten algorithms for the automated, post hoc correction of vertical drift. The reader may also wish to refer to Supplementary Item 1 where we present the algorithms in pseudocode alongside other technical details.

### Attach

The attach algorithm is the simplest of the algorithms considered in this paper. The algorithm simply attaches each fixation to its closest line. While this has the benefit of being extremely simple, it is generally not resilient to the kinds of drift phenomena described above. However, attach serves as a useful baseline algorithm, since it essentially corresponds to an eye-tracking analysis in which no correction was performed—a standard analysis of eye-tracking data would simply map fixations to the closest words or other areas of interest. We return to this point later in the paper.

### Chain

The chain algorithm is based closely on one of the methods implemented in the R package popEye (Schroeder, 2019) and can be seen as an extension of attach. Fixations are first linked together into “chains”—sequences of consecutive fixations that are within a specified *x* and *y* distance of each other. Fixations within a chain are then attached to whichever line is closest to the mean of their *y* values. This procedure is similar to the slightly more complex methods reported by Hyrskykari (2006) and Mishra et al., (2012), so we consider these to be special cases of chain.

The chain algorithm generally provides better performance over attach by exploiting the sequence’s order information. A disadvantage of the method, however, is that it is necessary to specify appropriate thresholds that determine when a new chain begins. If these thresholds are set too low, chain becomes equivalent to attach; if they are set too high, chain will group large numbers of fixations together and force them onto a single inappropriate line. By default, popEye sets the *x* threshold to 20 × the font height and the *y* threshold to 2 × the font height. It is not exactly clear how these defaults were chosen, but we would tentatively suggest that the *x* threshold should be set to approximately one long saccade length (we use 192 px), and the *y* threshold to around half a line height (we use 32 px).

### Cluster

The cluster algorithm is also based on one of the methods implemented in popEye (Schroeder, 2019). cluster applies *k*-means clustering^1^ to the *y* values of all fixations in order to group the fixations into *m* clusters, where *m* is the number of lines in the passage. Once each fixation has been assigned to a cluster, clusters are mapped to lines based on the mean *y* values of their constituent fixations: The cluster with the smallest mean *y* value is assigned to line one and so forth.

Unlike attach and chain, cluster does not assign fixations to the closest line in absolute terms; instead, it operates on the principle that fixations with similar *y* values must belong to the same line regardless of how far away that line might be. As such, the algorithm generally handles drift issues quite well. However, cluster will often not perform well if there is even quite mild overlap between fixations from different lines. In addition, since *k*-means clustering is not guaranteed to converge on the same set of clusters on every run, the cluster algorithm is nondeterministic and can be somewhat unpredictable across multiple runs on the same reading trial, which is an important consideration from the point of view of reproducible research output.

### Compare

The compare algorithm is directly based on the method reported by Lima Sanches et al., (2015) and is very similar to the more complex methods described by Yamaya et al., (2017). The fixation sequence is first segmented into “gaze lines” by identifying the return sweeps—long saccades that move the eye from the end of one line to the start of the next. The algorithm considers any saccade that moves from right to left by more than some threshold value (we use 512 px) to be a return sweep. Gaze lines are then matched to text lines based on a measure of similarity between them. Lima Sanches et al., (2015) considered three measures of similarity and found dynamic time warping (DTW; Sakoe & Chiba, 1978; Vintsyuk, 1968) to be the best method (we discuss DTW in more detail later in this section). Similarly, Yamaya et al., (2017) use the closely related Needleman–Wunsch algorithm (Needleman and Wunsch, 1970).

The gaze lines and text lines are compared in terms of their *x* values under the assumption that the fixations in a gaze line should have a good horizontal alignment with the centers of the words in the corresponding text line. Relying only on the *x* values helps the algorithm overcome vertical drift issues, but it is also problematic because in many standard reading scenarios the lines of text in a passage tend to be horizontally similar to each other; each line tends to contain a similar number of words that are of a similar length, resulting in potential ambiguity about how gaze lines and text lines should be matched up. To alleviate this issue, both Lima Sanches et al., (2015) and Yamaya et al., (2017) only compare the gaze line to a certain number of nearby text lines (we set this parameter to 3, which is effectively the closest line plus one line above and one line below).

### Merge

The merge algorithm is closely based on the post hoc correction method described by Špakov et al., (2019). The algorithm begins by creating “progressive sequences”—consecutive fixations that are sufficiently close together. This is similar to chain, except that the sequences are strictly progressive (they only move forward), so a regression will initiate a new progressive sequence. The original method uses several parameters to define what constitutes “sufficiently close together,” but here we boil this down to a single parameter, the y_thresh, which determines how close the *y* values of two consecutive fixations must be to be considered part of the same progressive sequence (we use 32 px).

Once these sequences have been created, they are repeatedly merged into larger and larger sequences until the number of sequences is reduced to *m*, one for each line of text. On each iteration of the merge process, the algorithm fits a regression line to every possible pair of sequences (with the proviso that the two sequences must contain some minimum number of fixations). If the absolute gradient of the regression line or its error (root-mean-square deviation) is above a threshold (we use 0.1 and 20 respectively), the candidate merger is abandoned. The intuition here is that, if two sequences belong to the same text line, the regression line fit to their combined fixations will have a gradient close to 0 and low regression error. Of the candidate mergers that remain, the pair of sequences with the lowest error are merged and added to the pool of sequences, replacing the original two sequences and reducing their number by one. This process is repeated until no further mergers are possible.

The algorithm then enters the next “phase” of the process, in which the criteria are slightly relaxed, allowing more mergers to occur. These phases could in principle be defined by the user, but we follow the four-phase model reported by Špakov et al., (2019), which effectively builds a set of heuristics into the algorithm. In Phase 1, the first and second sequences must each contain a minimum of three fixations to be considered for merging; in Phase 2, only the second sequence must contain a minimum of three fixations; in Phase 3, there is no minimum number of fixations; and in Phase 4, the gradient and regression error criteria are also entirely removed. Of course, as soon as the number of sequences is reduced to *m* the algorithm exits the merge process, so not all four phases will necessarily be required. Finally, the set of *m* sequences is matched to the set of text lines in positional order: The sequence with the smallest mean *y* value is mapped to line one and so forth.

A similar sounding method is reported by Beymer and Russell (2005) whose technique is based on “growing” a gaze line by incrementally adding fixations until this results in a poor fit to a regression line, at which point a new gaze line is begun. However, the description of the method lacked sufficient detail for us to consider it further.

### Regress

The regress algorithm, which is closely based on Cohen’s (2013) R package FixAlign, treats the fixations as a cloud of unordered points and fits *m* regression lines to this cloud. These regression lines are parameterized by a slope, vertical offset, and standard deviation, and the best parameters are obtained by minimizing^2^ an objective function that determines the overall fit of the lines through the fixations. The algorithm has six free parameters which are used to specify the lower and upper bounds of the slope, offset, and standard deviation. Here, we directly adopt FixAlign’s defaults: [− 0.1,0.1], [− 50,50], and [1,20], respectively. Once the *m* best-fitting regression lines are obtained, regress assigns each fixation to the highest-likelihood regression line, which itself is associated with a text line.

regress tracks FixAlign very closely, except that we did not implement the “run rule,” an option that is switched on by default in FixAlign. This option maps ambiguous fixations to the same line as the surrounding fixations, if the surrounding fixations were classified unambiguously (Cohen, 2013, p. 680). Cohen’s run rule is a more general method that could in principle be applied to the output of any algorithm, so in the interest of isolating the core concept of FixAlign and comparing all algorithms on a level playing field, we did not to implement the option here.

regress has some conceptual similarities with merge but differs in several important respects. Notably, regress takes a top-down approach, where merge is more bottom-up, and the regression lines that regress fits to the fixations cannot take independent values—it is assumed that all fixations are sloping in the same direction, with the same vertical offset, and with the same amount of within-line variance. In addition, unlike merge, regress does not utilize the order information; instead, like cluster, it views the fixations as a collection of unordered points.

### Segment

The segment algorithm is a slight simplification of the method described by Abdulin and Komogortsev (2015). The fixation sequence is first segmented into *m* disjoint subsequences based on the *m* − 1 most extreme backward saccades along the *x*-axis (i.e., the saccades that are most likely to be return sweeps). These subsequences are then mapped to the lines of text chronologically, under the assumption that the lines of text will be read in order. Abdulin and Komogortsev (2015) do not state precisely how they identify the return sweeps, but it seems they potentially allow for more than *m* subsequences to be identified, in which case, rereadings of a previous line, based on a threshold level of similarity, are discarded. The version of the algorithm considered here does not discard any fixations and instead always identifies exactly *m* subsequences.

The advantage of this general approach, as emphasized by Abdulin and Komogortsev (2015), is that the *y* values of the fixations are completely ignored, rendering any vertical drift entirely invisible to the algorithm. However, the approach does not allow for the possibility that the lines of text might be read out of order or that a line of text might be read more than once, which is not uncommon in normal reading behavior. Therefore, the great strength of segment—its identification of *m* consecutive subsequences, permitting a chronological, as opposed to positional, mapping—is also its great weakness: If a large regression is mistakenly identified as a return sweep, this will lead to a catastrophic off-by-one error in subsequent line assignments.

### Split

As far as we know, the split algorithm takes an approach that is distinct from anything previously reported, although it is conceptually similar to segment. Like segment, the split algorithm works on the principle of splitting the fixation sequence into subsequences by first identifying the return sweeps. However, split is not restricted to finding exactly *m* − 1 return sweeps; instead, it identifies the most likely set of return sweeps, however many that turns out to be. There are various ways of approaching this classification problem, but here we use *k*-means clustering to partition the set of saccades into exactly two clusters. Since return sweeps are usually highly divergent from normal saccades (i.e., a return sweep is usually represented by a large negative change on the *x*-axis), one of the two clusters will invariably contain the return sweeps, which can then be used to split the fixation sequence into subsequences. However, since this is not guaranteed to produce *m* − 1 return sweeps (and therefore *m* subsequences), an order-based mapping is not possible, so split must use absolute position: Subsequences are mapped to the closest text lines in absolute terms. split has the advantage of generally finding all true return sweeps, and even if it identifies some false positives, the resulting subsequences can still be mapped to the appropriate lines by absolute position. However, this also means the algorithm is less resilient to vertical drift issues.

### Stretch

The stretch algorithm is loosely based on the method proposed by Lohmeier (2015) and shares some similarities with Martinez-Gomez et al., (2012) and Nüssli (2011). Lohmeier’s (2015) original method was designed for the reading of source code and therefore takes advantage of the fact that code has very irregular line lengths and indentation levels. The method works by finding an *x*-offset, *y*-offset, and scaling factor that, once applied to the fixations, minimizes alignment error between the fixations and lines of text.

The framework we adopt herein never adjusts the *x* values, and we also assume that an ordinary passage of text is being read, so line length is substantially more constant than during code reading and therefore less informative. Therefore, we simplified the original method by dispensing with all dependencies on the *x* values. Instead, stretch finds a *y*-offset, *o*^∗^, and a vertical scaling factor, *s*^∗^, that minimizes the sum absolute difference between the corrected fixation positions (*f*_*y*_*s* + *o*) and the corrected fixation positions once attached to their closest lines. The equations presented in Lohmeier (2015, pp. 37–38) therefore simplify to:

1 $$ o^{\ast}, s^{\ast} = \operatornamewithlimits{arg min}_{o,s} \sum\limits_{f \in F} | (f_{y} s + o) - \text{attach}(f_{y} s + o) |,  $$

where attach(⋅) returns the *y*-axis position of the nearest line of text. In other words, the algorithm seeks a transformation of the fixations that results in minimal change following the application of attach.

To constrain the minimization problem, the user must specify appropriate lower and upper bounds for the offset and scaling factor, resulting in four free parameters. Here, we adopt offset bounds of [− 50,50], following the regress algorithm, and scaling factor bounds of [0.9,1.1]. Effectively, this means the algorithm can move the set of fixations up or down by up to 50 pixels and stretch their positions on the vertical axis by between 90% and 110%. While approaching the problem from a different angle, stretch is computationally similar to regress, except that it emphasizes systematic offset issues rather than systematic slope issues.

### Warp

The final algorithm we consider, warp, is novel to this paper, although it is mostly a wrapper around a preexisting algorithm—dynamic time warping (DTW; Sakoe & Chiba, 1978; Vintsyuk, 1968). DTW was used by the compare algorithm to provide a measure of dissimilarity between a gaze line and a text line. To our knowledge, however, there have been no previous reports of DTW being used directly to align fixations to text lines. This is somewhat surprising because DTW is the natural computational choice for tackling drift and alignment problems. The closest previously described method is Carl (2013), who uses a basket of reading-related measures to place a cost on different paths through a lattice of fixation-to-character mappings and selects the path with minimal cost. This is quite complex, however, and we consider it to be a special case of warp, which is a direct application of the standard DTW algorithm to eye-tracking data.

DTW is typically useful when you have two sequences, not necessarily of the same length, and you want to (a) calculate how similar they are (as is the case in the compare algorithm) or (b) align the two sequences by mapping each element in one sequence to a corresponding element in the other. For example, DTW may be used to calculate the similarity between a signature, which can be expressed as a sequence of *xy*-coordinates over time, and a reference signature (e.g., Lei & Govindaraju, 2005; Riesen et al.,, 2018). Importantly, the two sequences do not need to be perfectly matched in terms of overall magnitude or patterns of acceleration and deceleration for a good alignment to be found. In the case of signature verification, for example, it does not matter if the candidate signature has the same size as the reference or that it was drawn at the same speed, what matters is that there is a good match in the overall shape and that the strokes were drawn in the same order. DTW finds many other applications in, for example, genomics (Aach & Church, 2001), medicine (Caiani et al., 1998), and robotics (Vakanski et al., 2014).

**Fig. 2:**
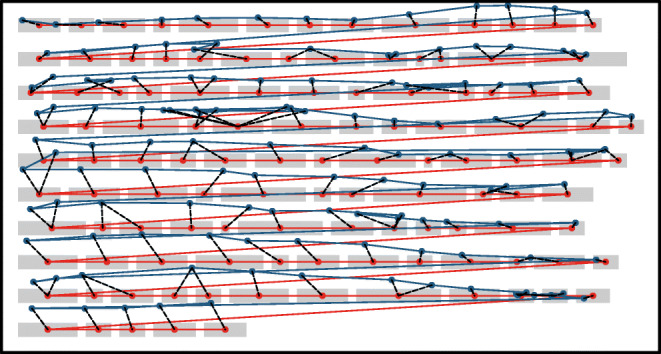
Illustration of the warp algorithm. The veridical fixation sequence is represented in *blue*, and the expected fixation sequence (the sequence of word centers) is represented in *red*. The *dashed black lines* show the DTW warping path—the optimal way to align the two sequences, such that the sum of the Euclidean distances between matched points (i.e., the sum of the dashed lines) is minimized

In order to use DTW to realign the fixation sequence to the text, we first need to specify an expected fixation sequence. Since we expect the reader to traverse the passage from left to right and from top to bottom, we can use the series of word centers as the expected sequence, under the assumption that readers will target the centers of words (O’Regan et al., 1984). Given the expected and veridical sequences as inputs, the DTW algorithm finds the optimal way to nonlinearly warp the sequences on the time dimension such that the overall Euclidean distance between matched points across the two sequences is minimized, while maintaining a monotonically increasing mapping.^3^ In the “warping path” that results from this process, every fixation is mapped to one or more words and every word is mapped to one or more fixations (see Fig. 2 for an example). It is then simply a case of assigning each fixation to whichever line its mapped word(s) belong(s) to. In the unlikely event that the mapped words belong to different lines, the majority line wins or an arbitrary choice is made in the case of ties.

If the final fixation on line *i* were mapped to the first word on line *i* + 1, this would result in a large increase in the overall cost of the mapping, so line changes act as major clues about the best alignment. The upshot of this is that warp effectively segments the fixation sequence into exactly *m* subsequences, which are mapped to the lines of text in chronological order. In this sense, warp behaves very much like segment. However, the additional benefit of warp is that it can simultaneously consider different possibilities about which saccades are the return sweeps, selecting only those that result in the best fit to the passage at a global level. Nevertheless, warp is ultimately limited by the veracity of the expected fixation sequence, which encodes one particular way of reading the passage—line by line from start to end. If the reader deviates from this assumption (e.g., by rereading or skipping lines), warp can fail to correctly assign fixations to lines.

### Summary

In this section we have described ten algorithms for aligning a fixation sequence to a multiline text, each of which takes a fundamentally different approach. A summary of the information utilized by the algorithms is provided in Table 1; each algorithm uses at least one piece of information about the fixations and at least one piece of information about the passage, and some also rely on additional parameters set by the user or built-in heuristics.

**Table 1:** Information utilized by the algorithms

	Fixation Information			Passage Information			Other Information	
Algorithm	*X*	*Y*	Order	No. Lines	Line *Y*	Word *X*	Parameters	Heuristics
attach		✓			✓			
chain	✓	✓	✓		✓		2	
cluster		✓		✓				
compare	✓	✓	✓		✓	✓	2	
merge	✓	✓	✓	✓			3	✓
regress	✓	✓		✓	✓		6	
segment	✓		✓	✓				
split	✓	✓	✓		✓			
stretch		✓			✓		4	
warp	✓	✓	✓		✓	✓		

Broadly speaking, the algorithms proceed in three stages, analysis, assignment, and update, the one exception being attach which has no analysis stage. In the analysis stage, the fixations are analyzed, transformed, or classified in some sense. The rationale behind this process varies by algorithm, but in general the algorithms can be categorized into those that classify the fixations into *m* groups (i.e., one group per text line; cluster, merge, regress, segment, and warp) and those that do not (attach, chain, compare, split, and stretch).

In the assignment stage, the fixations are assigned to text lines. If the analysis stage does *not* produce *m* groups, then assignment must be based on absolute position (or similarity in the case of compare, although it still uses absolute position to select neighboring lines to compare to). If the analysis stage *does* produce *m* groups, then they can be assigned to text lines based on order; this generally allows for better handling of vertical drift because absolute position is ignored. In the case of cluster, merge, and regress, which produce unordered groups at the analysis stage, groups are matched to text lines based on the order in which they are positioned vertically (i.e., mean *y* value). In the case of segment and warp, the groups are assigned to text lines in chronological order, which is only possible because these two algorithms produce subsequences that inherit the order of the original fixation sequence. An overview of the analysis and assignment methods is provided in Table 2 for quick reference.

**Table 2:** Summary of the analysis and assignment stages of each algorithm

Algorithm	Analysis stage	Assignment stage
attach	N/A	Assign fixations to closest text lines
chain	Chain consecutive fixations that are sufficiently close to each other	Assign chains to closest text lines
cluster	Classify fixations into *m* clusters based on their *y* values	Assign clusters to text lines in positional order
compare	Split fixation sequence into subsequences based on saccades that are longer than a threshold	Assign subsequences to text lines by measuring horizontal similarity with the words in neighboring text lines
merge	Form a set of progressive sequences and then reduce the set to *m* by repeatedly merging those that appear to be on the same line	Assign merged sequences to text lines in positional order
regress	Find *m* regression lines that best fit the fixations and group fixations according to best fit regression lines	Assign groups to text lines in positional order
segment	Segment fixation sequence into *m* subsequences based on *m* − 1 most-likely return sweeps	Assign subsequences to text lines in chronological order
split	Split fixation sequence into subsequences based on best candidate return sweeps	Assign subsequences to closest text lines
stretch	Find an offset and scaling factor that results in a good alignment between the fixations and lines of text	Assign transformed fixations to closest text lines
warp	Map fixations to word centers by finding a monotonically increasing mapping with minimal cost, effectively resulting in *m* subsequences	Assign fixations to the lines that their mapped words belong to, effectively assigning subsequences to text lines in chronological order

Finally, in the update stage, the original fixation sequence is modified to reflect the line assignments identified in the previous stage. In the versions of the algorithms reported in this paper, we always use the same update approach: The *y* values of the fixations are adjusted to the *y* values of the assigned lines, while the *x* values and the order of the fixations are always left untouched. In principle, however, there are other ways of performing the update stage (e.g., retaining the original *y*-axis variance or discarding ambiguous fixations).

## Performance on simulated data

We now test the ability of each algorithm to correctly recover the intended lines from simulated fixation sequences. These fixation sequences are simulated with particular characteristics, allowing us to understand how the algorithms respond to specific, isolated phenomena.

### Method

**Fig. 3:**
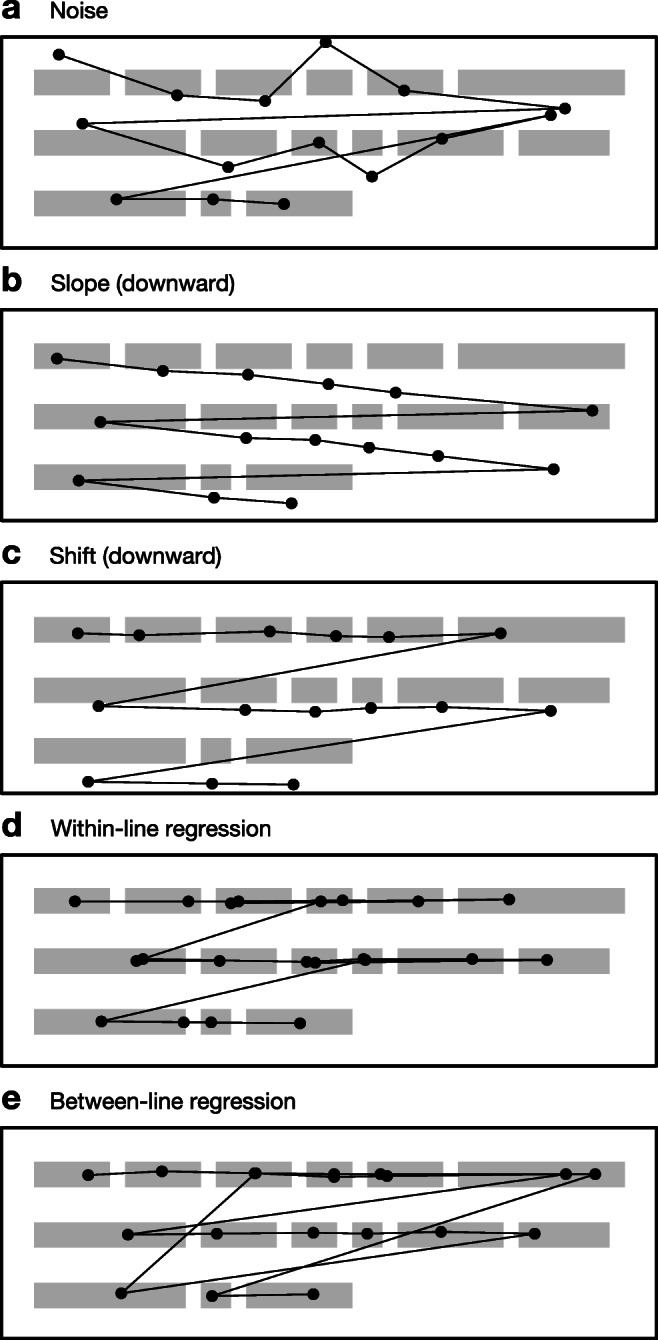
Example simulated fixation sequences under five phenomena considered in this paper. The algorithms must overcome these phenomena in order to correctly infer the intended line of each fixation

In each simulation, we generate a passage of “Lorem ipsum” dummy text consisting of between 8 and 12 lines with up to 80 characters per line and 64 px of line spacing. We then generate a fixation sequence consisting of one fixation for every word in the passage: The *x* value of a fixation (*f*_*x*_) is set randomly within the word; the *y* value of a fixation (*f*_*y*_) is calculated according to:

2 $$ f_{y} = \mathcal{N}(l_{y}, d_{\text{noise}}) + f_{x} d_{\text{slope}} + l_{y} d_{\text{shift}},  $$

where *l*_*y*_ is the vertical center point of the intended line—the *y* value that the reader is targeting. This models three types of distortion: noise, slope, and shift. Additionally, we simulate two types of regression that are characteristic of normal reading behavior but which can nevertheless disrupt algorithmic correction. Together, these five phenomena are illustrated in Fig. 3 and described below.

#### Noise distortion

The noise distortion parameter, *d*_noise_, controls the standard deviation of the normally distributed noise around the intended line and represents imperfect targeting by the reader and/or measurement error. In our exploration of this parameter, we use values of *d*_noise_ = 0, representing no noise, through *d*_noise_ = 40, representing extreme noise. The noise parameter is also a proxy for line spacing (raising the noise level effectively corresponds to tightening the line spacing), so this parameter also provides an indication of how the algorithms will perform under different degrees of line spacing.

#### Slope distortion

The slope distortion parameter, *d*_slope_, controls the extent to which fixations progressively move downward as the reader moves from left to right across the passage; fixations on the left edge of the passage will be correctly located, but for every one pixel the reader moves to the right, the fixations will drift downward by *d*_slope_ pixels. Unlike noise, this is solely attributable to measurement error. In our exploration of this parameter, we use values of *d*_slope_ = − 0.1, representing extreme upward slope, through *d*_slope_ = 0.1, representing extreme downward slope.

#### Shift distortion

The shift distortion parameter, *d*_shift_, controls the extent to which fixations progressively move downward as the reader moves from one line to the next; fixations on the first line will be correctly located, but for every one pixel of intentional downward movement, the fixations will drift downward by a further *d*_shift_ pixels. Like slope, this represents systematic measurement error. Our exploration of this parameter uses values of *d*_shift_ = − 0.2, representing extreme upward shift, through *d*_shift_ = 0.2, representing extreme downward shift.

#### Within-line regression

As mentioned above, we also consider the effects of two types of regression. The first of these is within-line regressions, which is where the reader momentarily jumps back to a previous point in the current line. The extent to which the reader performs within-line regressions is formalized by a probability. If this probability is set to 1, the reader will perform a regressed fixation after every normal fixation, doubling the number of fixations on the line; if the parameter is set to 0, the reader will never perform a regression within the line. The *x* position of the regressed fixation is located randomly between the start of the line and the current fixation with longer regressions being linearly less probable than shorter regressions. The *y* value of the regressed fixation follows Eq. 2.

#### Between-line regression

The second type of regression, between-line regressions, is where the reader rereads text from a previous line. Between-line regressions are expressed in terms of the probability that the reader will go back to a previous line at some point during reading of the current line. Once the regression is completed, the reader returns to the point in the passage before the regression occurred. If the parameter is set to 1, the reader will reread part of a previous line for every line they read; if it is set to 0, the reader will never perform a regression to a previous line. When a between-line regression occurs, the previous line is determined randomly, with more recent lines linearly more probable than less recent lines; the section of the previous line is determined randomly by two uniformly distributed *x* values. The *y* values of the regressed fixations follow Eq. 2.

### Results

For each phenomenon, we ran 100 simulations for each of 50 gradations in the parameter space, and each of these 5000 simulated reading scenarios was corrected by all ten algorithms. Accuracy is measured as the percentage of fixations that were correctly mapped back to the target line. Before describing the results, there are three important things to note. Firstly, the extreme values we have chosen for each phenomenon are arbitrary, so the algorithms should only be compared within, and not across, phenomena.^4^ Secondly, we have not modeled the interactions between phenomena because it is inherently difficult to explore the effects of five dimensions on accuracy and it is not clear how the dimensions should be weighted a priori. Thirdly, for the algorithms that have free parameters (chain, compare, merge, regress, and stretch), we use the default parameter settings defined in the previous section. We have not systematically manipulated the parameter settings because (a) this would result in an explosion in the number of algorithm/parameter combinations that we must consider, (b) manipulating a parameter to deal with one phenomenon can have unexpected consequences for other phenomena,^5^ and (c), in a sense, these algorithms ought to incur a penalty for not being parameter-free.

#### Performance on noise

**Fig. 4:**
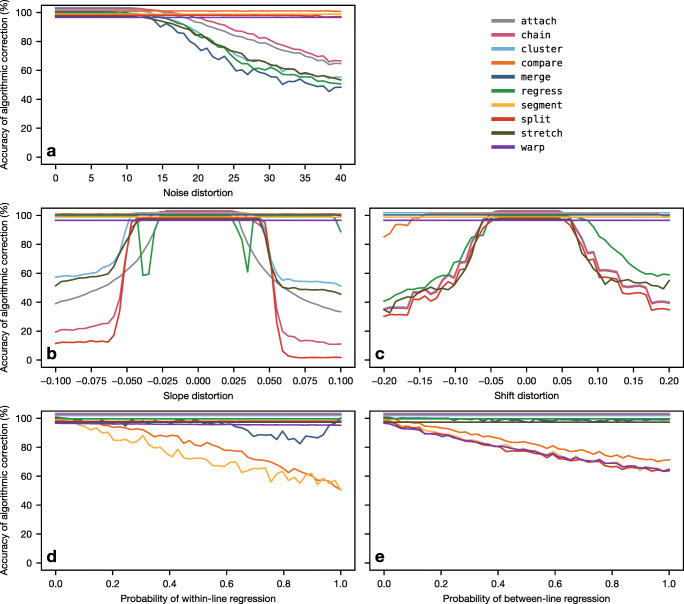
Mean accuracy of the ten algorithms in response to the five eye-tracking phenomena. For example, some algorithms (attach, chain, cluster, merge, regress, and stretch) are adversely affected as the noise level is increased, while the other algorithms are either resilient to noise (compare and split) or entirely invariant to noise (segment and warp). The plotted lines have been vertically staggered to aid visualization

Results for the noise distortion parameter are shown in Fig. 4a. Under zero noise, all algorithms perform at 100% accuracy, but six of the algorithms are adversely affected by noise when it reaches a sufficiently high level of around 10: Of these, chain performs best, closely followed by attach, then cluster, regress, and stretch, and finally merge. Of the remaining algorithms, compare and split are highly resilient to noise, while segment and warp are entirely invariant.

#### Performance on slope

In terms of slope distortion (Fig. 4b), when the parameter is set to zero, all algorithms perform perfectly, but as the slope becomes more extreme (in either the upward or the downward direction), five of the algorithms experience a sustained loss in accuracy. Of these, cluster and stretch generally perform best and, initially at least, attach performs worst; chain and split initially perform better than attach, but are eventually outperformed. Interestingly, although regress is mostly resilient to slope, it has two weak spots around the values of − 0.03 and 0.03. When the slope takes one of these values, regress struggles to disambiguate between (a) zero offset combined with the appropriate slope and (b) a large offset combined with slope in the opposite direction; if it selects the wrong option, fixations on one half of the passage will be misaligned, causing a substantial drop in accuracy. This reveals a hidden weakness of the regress algorithm, and we will see an example of it later. Of the remaining algorithms, compare and merge are highly resilient to slope, while segment and warp are invariant.

#### Performance on shift

In terms of shift (Fig. 4c), when the parameter is zero, all algorithms perform perfectly, but as it becomes more extreme, five of the algorithms—attach, chain, regress, split, and stretch—drop in accuracy. In fact, attach, chain, and split produce identical results in the case of shift because they are all fundamentally reliant on absolute position. Somewhat surprisingly, stretch does not perform especially well on shift. This is because stretch can only handle up to one full line of shift; any more than this and the bounds have to be relaxed, but this results in an objective function with multiple maxima which is difficult to optimize. The compare algorithm is mostly resilient to shift, while the remaining four algorithms—cluster, merge, segment, and warp—are invariant.

#### Performance on within-line regression

Results for within-line regressions are shown in Fig. 4d. When there are no within-line regressions, all algorithms perform at 100%, but three of the algorithms drop off as the probability of within-line regression is increased. Of these, compare and segment track each other quite closely because they rely on identifying the return sweeps; merge is generally quite resilient, except when the parameter is around 0.7–0.9 because these values cause a large number of progressive sequences to be generated which cannot then be merged very freely, so the merge process tends to get trapped in local minima (i.e., bad mergers that happen early on cannot later be reverted). Of the remaining algorithms, split^6^ and warp are highly resilient, while attach, chain, cluster, regress, and stretch are invariant.

#### Performance on between-line regression

In terms of between-line regressions (Fig. 4e), four algorithms are negatively impacted by increases in this parameter. Of these, compare and split can in principle find more than *m* gaze lines, but they have difficulties identifying when a between-line regression occurs, while segment and warp are limited to identifying exactly *m* gaze lines in strictly sequential order, so they fundamentally cannot handle between-line regressions. Of the remaining algorithms, merge is resilient to between-line regressions, while attach, chain, cluster, regress, and stretch are entirely invariant.

### Summary

**Fig. 5:**
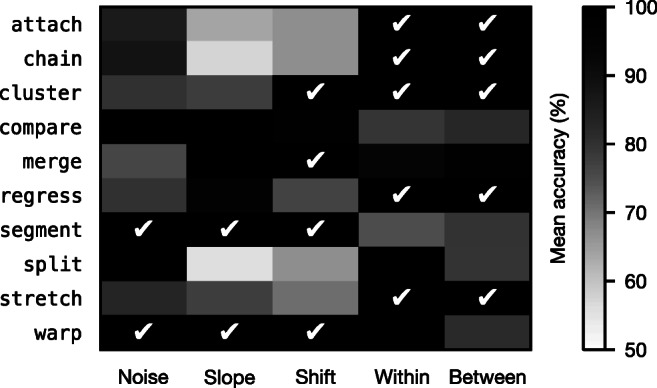
Mean accuracy of the algorithms for each of the eye-tracking phenomena. *Darker cells* indicate phenomena that an algorithm performs well on. A *check mark* indicates that the algorithm is entirely invariant to the phenomenon in question, scoring 100% in all 5000 simulations

In this section, we have simulated five eye-tracking phenomena that are particularly relevant to understanding the performance characteristics of the algorithms. Fig. 5 summarizes how accurately the algorithms perform on each phenomenon. No single algorithm is invariant—or even resilient—to all phenomena, although merge and warp come quite close: merge is only weak on noise, while warp is only weak on between-line regressions. In general, there tends to be a tradeoff between how well an algorithm can handle distortion and how well it can handle regressions; the ability to deal with one tends to come at the cost of the other. Nevertheless, in real world scenarios, performance will very much depend on the degree and relative prevalence of the phenomena. Furthermore, there are likely to be other important forms of measurement error and reading behavior that we have neglected to consider here, and those that we have considered are likely to interact in complex, unpredictable ways. It is therefore important to test the algorithms against natural eye-tracking data to get a more holistic understanding of their performance.

## Performance on natural data

In this section, we test the algorithms against an eye-tracking dataset that has been manually corrected by human experts. Unlike the simulations, there is no ground truth, and we cannot isolate particular phenomena; however, the benefit of this approach is that the phenomena are combined in a realistic way, allowing us to estimate how well the algorithms are likely to perform in real-world scenarios.

### Method

**Fig. 6:**
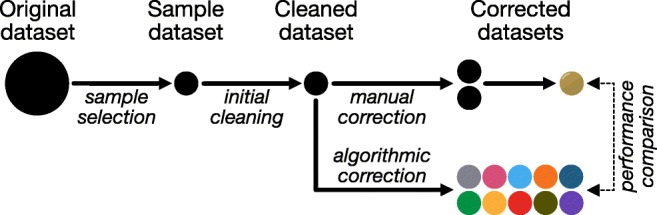
Pipeline for testing the algorithms on a natural dataset. The original dataset was first reduced to a smaller sample, which then underwent some initial cleaning steps. This cleaned dataset was then corrected by the ten algorithms and two human correctors, whose corrections were merged to form the gold standard. Performance is measured by how closely the algorithmic corrections match the gold standard correction

We tested the algorithms on an eye-tracking dataset collected by Pescuma et al., (in prep), which includes reading data for both adults and children, allowing us to test the algorithms on two distinct populations. Our general approach is illustrated in Fig. 6 and discussed over the following sections.

#### The dataset

Pescuma et al., (in prep) collected eye-tracking data for 12 passages from Italian children’s stories (e.g., a passage from *Goldilocks*). The passages were around 130 words in length, spanning 10–13 lines and were presented in 20-point Courier New (each character occupying around 0.45 degrees of visual angle). Either of two sets, each comprised of six passages, was administered for silent reading to a large sample of children aged 8–11 (*N* = 140) and a smaller sample of adult controls (*N* = 33) for a total of 877 reading trials. Eye movements were recorded using a tower mounted EyeLink 1000 Plus eye tracker (SR Research, Toronto, Canada) for which a typical accuracy of 0.25–0.50 degrees is reported by the manufacturer. Recording was monocular (right eye) with a 1000-Hz sampling rate.

#### Selection of the sample for manual correction

Since it was impractical to manually correct all 877 trials (to do so would require months of work), we selected a sample for manual correction. For each of the 12 passages, we selected two reading trials by adult participants and two reading trials by child participants, for a total of 48 trials (5.5% of the full dataset). The reading trials were selected pseudorandomly such that no single participant was represented more than once. Additionally, we manually checked and adjusted the sample to ensure it contained an equal balance of easy and challenging cases, as well as examples of all the various eye-tracking phenomena discussed previously.

#### Initial data cleaning

We performed two initial cleaning steps in order to isolate the core problem of line assignment from two extraneous issues. Firstly, any fixation that was located more than 100 px from any character in the passage was removed (i.e., out-of-bounds fixations that occur in the margins or off-screen). This is because the algorithms are not designed to detect and discard these fixations, and such cases can hinder their ability to match fixations to the appropriate lines. Secondly, prior to reading a passage and on its completion, a reader’s fixations will typically jump around the text unpredictably; again, since the algorithms are not designed to automatically discard such fixations, we manually removed any such cases from the starts and ends of the fixation sequences, allowing the algorithms to concentrate on the core problem of assigning fixations to lines.

#### Manual correction procedure

The cleaned sample dataset was corrected independently by two human correctors (JWC and VNP). To perform the correction, each corrector studied plots of the participants’ fixation sequences and recorded, fixation by fixation, which line each one belonged to, guided by fixation position, saccade trajectories, textual cues, and fixation duration, as well as general knowledge of eye tracking and reading behavior. Unlike the algorithms, the human correctors also had the option to discard fixations as they saw fit. This is because there were cases where it was clear a fixation should be discarded—for instance, due to spatial misplacement or ultra-short duration—and it would have therefore felt disingenuous to assign these cases to a line anyway.

Across the 48 reading trials, the correctors initially disagreed on 299 of 10,245 fixations (2.9%). Of these 299 disagreements, only 15 related to which line a fixation was assigned to; on inspection, all 15 cases turned out to be human error on the part of one corrector or the other. The other 284 disagreements related to whether or not a fixation should be discarded; following discussion of these cases, the correctors reached consensus about how these fixations should be treated. This resulted in a single manual correction, which we consider to be the gold standard against which the algorithms can be evaluated. In this gold standard correction, a total of 255 fixations were discarded across all 48 trials (2.5%; 5.3 fixations per trial).

It is interesting to note that although the two correctors had slightly different intuitions about when it was appropriate to discard a fixation, they essentially had perfect agreement about which line a fixation ought to be assigned to if it was retained. This suggests that the correction of vertical drift is actually quite objective—there is usually an unequivocally correct solution to any given trial, even if that solution may be difficult and time-consuming to obtain.

### Results

We analyze the performance characteristics of the algorithms in four ways. Firstly, we look at how the algorithms fare against the gold standard manual correction; secondly, we look at what proportion of trials are likely to be usable following drift correction; thirdly, we look at how the algorithms perform in comparison to using no drift correction at all; and finally, we look at how the algorithms relate to each other, regardless of their accuracy.

#### Accuracy against the gold standard

**Fig. 7:**
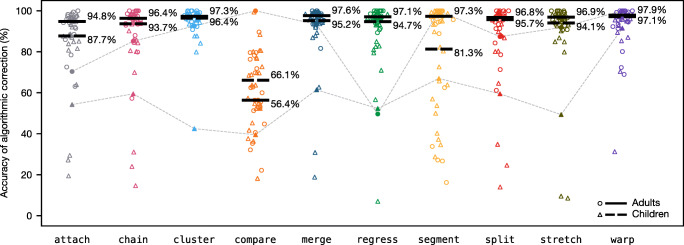
Accuracy of the algorithms on adult reading trials (*circles*) and child reading trials (*triangles*). The *y*-axis measures the percentage of fixations assigned to the correct line, as defined by the gold standard manual correction. The *filled points*, linked together by *dashed lines*, correspond to the two example trials illustrated in Figs. 8 and 9. The *black bars* show median accuracy for the adults (*solid bars*) and children (*broken bars*)

As with the simulations, accuracy is measured as the percentage of fixations that the algorithm mapped to the correct line; the ground truth is defined by the gold standard manual correction. In cases where the correctors chose to discard a fixation, the algorithm is automatically wrong, which amounts to a constant baseline level of error that all algorithms suffer from equally. Figure 7 plots accuracy on the 48 sample trials by algorithm. The most striking result is compare with overall median accuracy of 61.2%, substantially worse than all other algorithms.^7^ This contrasts with our simulations, which indicated that compare should at least be relatively strong on distortion. The reason for this discrepancy is that the simulated fixation sequences were generated directly from the lines of text with one fixation per word, so the artificial gaze lines that compare identified tended to be very horizontally similar to the artificial text lines. In the natural dataset, however, this is not the case; when the data contains a lot of natural noise and regressions, gaze lines cannot be reliably matched to text lines based on similarity, even if the set of candidate text lines is narrowed down to the three closest neighbors. Given that compare exhibited such poor performance, we consider it to be an algorithmic dead end and do not discuss it any further.

Of the remaining algorithms, median accuracy is typically around 95%, the worst performer being attach at 92% and the best performer being warp at 97.3%. Accuracy on child trials tends to be lower and more variable than accuracy on adult trials; however, the difference in medians was usually quite small. The major exception to this was segment for which median accuracy on adult trials was 97.3%, while median accuracy on child trials was 81.3%, making segment one of the best algorithms in terms of adult data but one of the worst in terms of child data. This may be because children tend to perform more regressions (e.g., Blythe & Joseph, 2011) and have more disfluent return sweeps (e.g., Parker et al.,, 2019), both of which create obstacles for the segment algorithm.

**Fig. 8:**
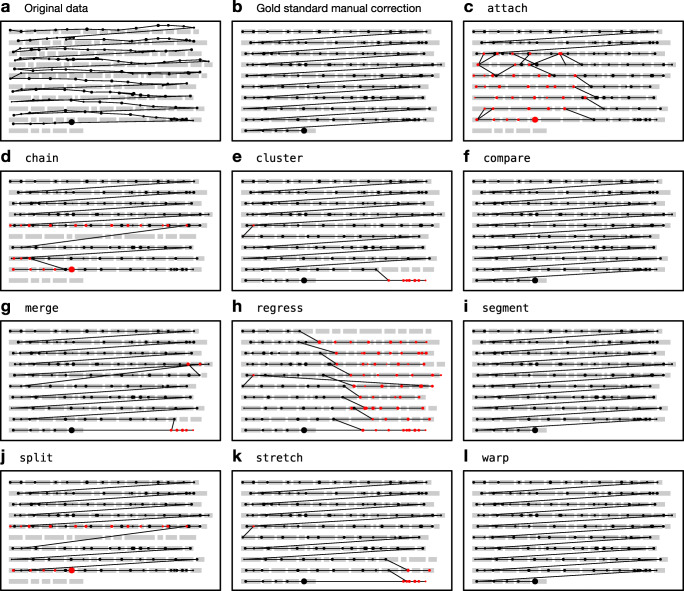
Original data and corrections of an adult trial. Fixations *in red* have been assigned to the wrong line. The algorithmic corrections correspond to the filled circles in Figs. 7 and 11

Median performance alone conceals the fact that accuracy is often highly variable and long tailed. In the best-case scenario, an algorithm will produce a perfect correction that is identical to the gold standard—all algorithms (even compare) scored 100% in at least one trial. In the worst-case scenario, an algorithm will perform as low as 10–30% accuracy. In addition, the algorithms often differ markedly on particular trials. We have highlighted this in Fig. 7 by singling out two trials, one by an adult and one by a child, which are represented by the filled data points that are linked together with dashed lines. Algorithmic corrections of the adult trial (filled circles) are depicted in Fig. 8. In this particular case, compare, segment, and warp were able to correctly recover the intended line of every fixation. However, the trial presented problems for some of the other algorithms; in particular, attach failed to handle the upward shift in the lower left quadrant of the passage, and regress misinterpreted the situation as a case of upward slope, resulting in fixations on the right-hand side of the passage being forced down by one line, a potential weakness highlighted by our simulations.

**Fig. 9:**
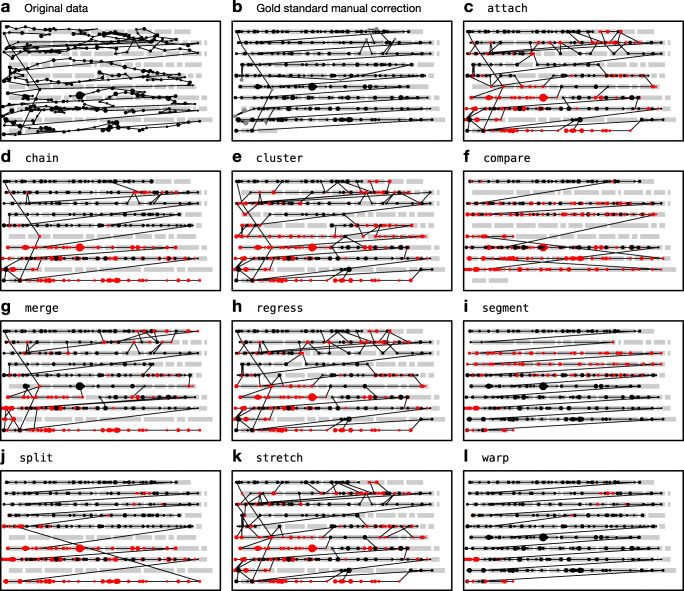
Original data and corrections of a child trial. Fixations *in red* have been assigned to the wrong line. Fixations that were discarded in the gold standard manual correction are shown in *gray*. The algorithmic corrections correspond to the filled triangles in Figs. 7 and 11

Fig. 9 depicts the algorithmic corrections of the child reading trial, which are represented by the filled triangles in Fig. 7. Performance on this trial is much worse due to the large amount of noise. cluster, for example, has struggled to correctly classify the fixations due to the large amount of overlap between fixations intended for adjacent lines, and segment has identified one particularly long within-line regression as a return sweep, resulting in some misalignment in the middle of the passage. Only warp was able to recover the intended lines for the majority of fixations, and the few errors it did make appear to be cases where the correctors chose to discard some fixations. Overall, these two example trials highlight that, although the algorithms have a similar level of performance on average, performance on a particular trial can be quite divergent depending on its particular characteristics. Illustrated corrections of all 48 trials by each algorithm can be found in Supplementary Item 2.

#### Proportion of corrections likely to be usable

**Fig. 10:**
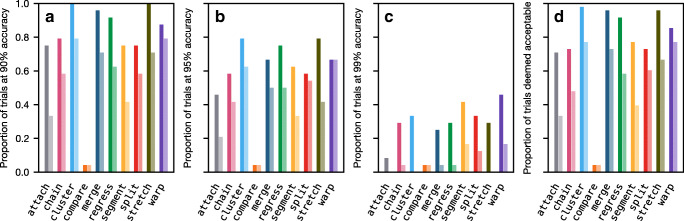
Proportion of trials that surpassed **a** 90%, **b** 95%, and **c** 99% accuracy, and **d** the proportion of corrections deemed acceptable by two human raters. The *dark* and *light bars* represent the adult and child datasets, respectively

Fig. 10 reports the proportion of corrections that surpassed an accuracy level of 90%, 95%, and 99% by algorithm. If you are willing to accept relatively low accuracy at the trial level (e.g., 90%, Fig. 10a), then cluster, merge, and stretch will provide the best performance—a large proportion of corrections will meet this criterion. In comparison, if you have more stringent accuracy requirements at the trial level (e.g., 99%, Fig. 10c), then segment, split, and warp are likely to provide better performance. Of course, the cost of a more stringent accuracy criterion is that fewer corrections will be usable overall, and it is not possible to know which trials have low accuracy in the absence of manual correction data. This highlights the fact that it is currently not possible to confidently achieve a high level of accuracy in a high proportion of trials, so researchers may still need to invest a significant amount of time if a high level of accuracy is demanded.

To estimate how usable the corrections are likely to be to a typical researcher, we performed a more subjective analysis of their quality. All 480 algorithmic corrections were presented blind and in random order to two raters (JWC and VNP), who independently classified every correction as either “acceptable” or “needs more work.” The raters did not discuss in advance what criteria they would use to make these judgments, but agreement was nevertheless very high at 94%. In addition to the overall number of errors, the raters weighed up other factors, such as how the errors were distributed over the passage, how challenging the input data seemed to be, and what effect the errors might have for downstream analyses. The results, which are shown in Fig. 10d, suggest that cluster, merge, and stretch are likely to produce very satisfactory results on adult data.

#### Improvement over no drift correction

Another way to measure performance is in terms of how much of an improvement an algorithm provides in comparison to applying no drift collection at all. To estimate this, we first need to define a baseline level of accuracy. As mentioned previously, the attach algorithm essentially corresponds to a standard eye-tracking analysis; it is equivalent to drawing maximal, nonoverlapping bounding boxes around the words in a passage and then mapping fixations to whichever bounding box they fall into (as would be the case in a standard analysis of eye-tracking data using the widely adopted area-of-interest paradigm). Therefore, we can estimate the potential improvement that a given algorithm offers by comparing its accuracy to the accuracy of the attach algorithm.

**Fig. 11:**
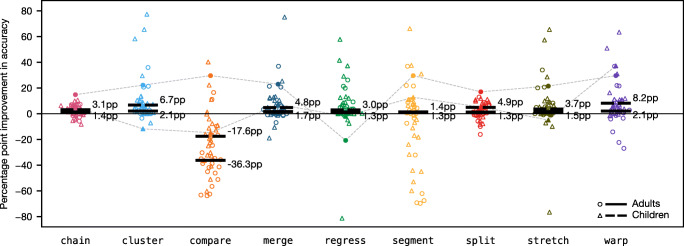
Improvement in accuracy in comparison to performing a standard eye-tracking analysis with no drift correction. The *y*-axis measures the percentage point increase (or decrease) in accuracy beyond the baseline accuracy of the attach algorithm. The *filled points*, linked together by *dashed lines*, correspond to the two example trials illustrated in Figs. 8 and 9. The *black bars* show median improvement for the adults (*solid bars*) and children (*broken bars*)

The results of this analysis are plotted in Fig. 11. The *y*-axis shows the percentage point increase (or decrease) in accuracy that results from applying vertical drift correction. The zero line represents the baseline of no drift correction (equivalent to attach). As before, the datapoints themselves tell us a lot more than the medians. The chain and split algorithms tend to be quite conservative, while the others tend to have more extreme effects. In the best case, cluster resulted in a 77 percentage point increase in accuracy in comparison to leaving the data uncorrected (i.e., attach = 19%, cluster = 96%); while in the worst case, regress resulted in an 81 percentage point drop in accuracy, badly corrupting the original input data (i.e., attach = 88%, regress = 7%).

These results highlight that, although in most cases the application of vertical drift correction can improve data quality, the process is not without risk. Furthermore, there is potentially more to gain from applying drift correction to child data, since the baseline level of accuracy tends to be lower to begin with; for example, warp offered a modest 2.1 percentage point increase in accuracy on adult data but an 8.2 percentage point increase on child data.

#### Relationships between algorithms

As noted above, some algorithms tend to produce very similar output where others produce quite different output. This raises the issue of how the algorithms relate to each other regardless of their performance characteristics on real or simulated data. To investigate this, for each pair of algorithms, we measured the DTW distance between the corresponding algorithmic corrections of each of the 48 sample trials and took the median distance as an estimate of how dissimilar those two algorithms are.^8^ We then analyzed the pairwise distances in two ways.

**Fig. 12:**
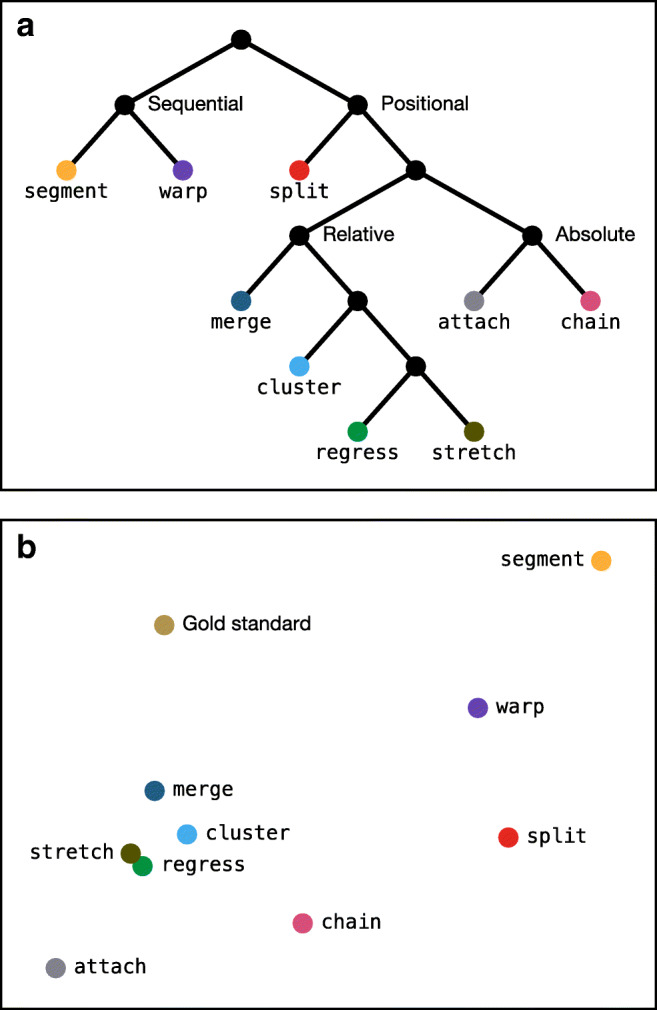
**a** Hierarchical clustering analysis of the algorithmic outputs, providing an approximate taxonomy of the algorithms. **b** Multidimensional scaling analysis of the algorithmic outputs; the distance between two algorithms corresponds to how dissimilar their corrections tend to be, so the space as a whole approximates how the algorithms relate to each other on two hypothetical dimensions

Firstly, we used agglomerative hierarchical clustering to produce a dendrogram (see Fig. 12a), which yields an approximate taxonomy of the algorithms based on their similarity. The root node represents all algorithms, which initially fork into two major groups. The “sequential algorithms,” segment and warp, both operate on the principle of identifying the return sweeps and mapping the resulting subsequences to the lines of text in sequential order; in other words, their analysis stages can only produce groups consisting of fixations that were arranged consecutively in the original fixation sequence. This means they tend to produce similar outcomes—they both, for example, force fixations onto inappropriate lines in order to preserve sequentiality. Of the “positional algorithms,” split is the first to branch off, perhaps because—like the sequential algorithms—it leans heavily on the return sweeps, and among the remaining algorithms, there is a clear dichotomy between those that assign based on relative position (cluster, merge, regress, and stretch) and those that assign based on absolute position (attach and chain).

Secondly, we used multidimensional scaling to locate the algorithms in some latent “algorithm space.” Fig. 12b shows the output of this analysis projected into two hypothetical dimensions: Algorithms that are close together in this space tend to produce similar results, while algorithms that are far apart tend to produce dissimilar results. The two dimensions of the space appear to roughly correspond to the algorithms’ analysis strategy (*x*-axis) and assignment strategy (*y*-axis). split, for example, shares its analysis strategy with segment (it groups fixations based on return sweeps), but it has an assignment strategy that is more similar to chain (it assigns based on absolute position). We also see that regress and stretch tend to produce very similar output and are therefore likely to be somewhat interchangeable. Interestingly, the human correctors—represented by the gold standard manual correction—are located in a relatively unexplored part of algorithm space: Their analysis strategy appears to be more similar to chain or merge (finding local linear clusters), while their assignment strategy seems to be more global and sequential, like warp. Anecdotally, this aligns with our experience of performing the manual corrections, and this observation is suggestive of fertile ground for the future development of correction algorithms.

### Summary

In this section, we tested the ten algorithms on a real eye-tracking dataset. Although warp was marginally the most performant algorithm across the majority of measures, our results indicate that the best algorithm will largely depend on the particular characteristics of a given trial, as well as the general characteristics of the dataset being corrected. All 48 reading trials could be improved by at least one of the algorithms; the difficulty for the researcher, of course, is in knowing which algorithm to apply to a given trial in the absence of a gold standard.

## Discussion

We have identified ten core approaches to the methodological problem of correcting vertical drift in eye-tracking data. We instantiated each of these approaches as a simple algorithm that can be evaluated in a consistent and transparent way. Our first analysis using simulated data allowed us to identify which phenomena the algorithms are invariant to and to quantify how the algorithms respond to increasing levels of those phenomena. Our second analysis validated the algorithms on a real eye-tracking dataset and allowed us to strengthen our qualitative intuitions about their similarities and differences. In the remainder of the paper, we sum up what we learned about the algorithms, provide some practical guidance for researchers in the field, and conclude with some thoughts about how vertical drift correction can be improved going forward.

### Major properties of the algorithms

The algorithms can be placed into three major categories. The **sequential algorithms**, segment and warp, hinge on their ability to correctly identify the return sweeps. If successful, these algorithms have excellent performance because any vertical drift in the data essentially becomes invisible. However, if the text is read nonlinearly, the premise on which the sequential algorithms are based breaks down. Therefore, one should apply these algorithms with great caution when the data are rich in regressions, either within-line (which warp tends to handle well) or between-line. The risk is particularly high with segment, which has good median performance on adult data, but can also lead to catastrophic errors if large regressions are mistakenly interpreted as return sweeps.

The **relative-positional algorithms**, cluster, merge, regress, and stretch, are mostly dependent on their ability to correctly classify the fixations into *m* groups, each using a slightly different technique to do so. So long as the identified groups are sound, then the use of relative position to assign the fixations to lines is generally very resistant to vertical drift.

The **absolute-positional algorithms**, attach, chain, and split, generally tend to be the worst at dealing with vertical drift because they assign based on absolute position; this feature makes them generally weaker than other algorithms at dealing with noise, slope, and shift. However, a benefit of these three algorithms is that they tend to be quite conservative and do not make dramatic changes to the data, which makes them a reasonable choice for researchers who would prefer a more minimalist data transformation or whose data are not overly affected by distortion issues.

### General guidance for researchers

The analyses presented in this paper clearly indicate that each algorithm performs best on a different set of factors, some of which we have not considered here in detail. For example, if the line spacing is quite tight, the eye-tracking data is more likely to be negatively impacted by distortion, making a sequential algorithm a better choice; conversely, if lines are spread far apart, a relative-positional algorithm may be more appropriate. Overall, different sets of data will require different correction algorithms, and a qualitative inspection of the data will be required to detect the relative severity of general noise, drift issues, and regression phenomena. To help in this process, one option might be to hand-correct a sample of the trials in order to assess which algorithm performs best on those specific cases and then apply this algorithm to the entire dataset. However, there might very well be too much trial-by-trial (or participant-by-participant) variability to use a single algorithm across an entire dataset. In this case, it may be preferable to create subsets of data exhibiting comparable patterns of eye-tracking phenomena and deal with those subsets with different algorithms. Another idea would be to run several algorithms over the dataset and manually inspect cases where there is disagreement.

Recording children’s eye movements poses extra challenges relative to adults’, particularly due to the difficulty that younger participants often experience sitting still for relatively long periods of time (Blythe & Joseph, 2011), which can lead to a loss of calibration. Therefore, especially in the case of multiline reading, developing readers’ eye movements are generally characterized by more noise, as well as by greater slope and shift, than adults’. This would suggest resorting to algorithms like segment and warp, which are entirely invariant to noise, slope, and shift (Fig. 5). However, children tend to generally make more regressions than adults (e.g., Blythe & Joseph, 2011; Reichle et al.,, 2003), which is exactly the phenomenon that affects segment and warp the most. The general tradeoff between the ability to handle distortion and the ability to handle regressions is at play here, and only an attentive, qualitative check of the data will tell the researcher which way to go. If there does not appear to be too much of an issue with between-line regressions, then warp is probably the best choice; otherwise, cluster or merge might be a better option.

Regarding the practicalities of the algorithm application pipeline, we suggest performing a few cleaning steps before drift correction, in order to isolate the line assignment problem from other issues that the algorithms considered here were not designed to deal with, and which would otherwise impair their performance. For example, researchers may first want to discard any fixations that lie beyond the text area and merge or eliminate extremely short fixations. Only after these basic cleaning steps have been performed, can algorithmic correction be safely applied.

Another important aspect to consider is the presence of free parameters. The chain, merge, regress, and stretch algorithms take additional input parameters that must be set appropriately by the user. In practice, Špakov’s (2019) and Cohen’s (2013) suggested defaults for the merge and regress algorithms seemed to work well on our test dataset, but in the case of chain, it was somewhat unclear how to set the *x* and *y* thresholds appropriately, so experimentation might be required to produce the best results. We also found that stretch was very sensitive to its parameter settings and that the upper and lower bounds must be tightly constrained around likely values for it to produce sensible results. An advantage of all other algorithms is that they are parameter free, making drift correction easier to perform, document, and justify.

It is also worth considering the complexity of the algorithms. Some, such as chain and segment, are very simple and intuitive, while others, such as merge and warp, are quite complex. Although complexity is not an important consideration from a performance perspective (in general, we should prefer whichever algorithm works best), it is worth considering how complexity might impinge on real-world use. For example, users may be less inclined to use an algorithm if they cannot intuitively understand how it will manipulate their data, so algorithms should, where possible, be designed in a way that researchers find easy to understand and easy to convey to their readership. In that regard, we hope that this paper will give researchers more confidence in the algorithms, which we have validated and benchmarked.

Finally, it is worth noting that most of the algorithms have linear time complexity and can process a reading trial in fractions of a second, so runtime does not warrant any special consideration. The one exception to this is merge which scales quadratically with the number of fixations; in our testing, for example, it took 100 ms for a trial consisting of around 100 fixations but up to 31 s for a trial of around 500 fixations.

### Improvements on the algorithms

We would not wish to claim that the algorithms, as presented here, are the only approaches one may take nor that they are the ultimate form of each core method; all can be improved in one way or another. Furthermore, there are likely to be ways of combining the outputs of multiple algorithms to increase confidence in particular solutions. The goal of this paper, however, was to evaluate the algorithms in their more abstract, idealized forms in order to make general recommendations and to provide a solid foundation for the future development of vertical drift correction software. Nevertheless, here we briefly note some of the most obvious ways in which the algorithms could be improved.

#### Chain

The main weakness of the chain algorithm is its reliance on threshold parameters that must be set by the user, but this situation could be improved if the parameters defaulted to sensible values based on reliable heuristics. For example, it may be the case that the parameters can be reliably estimated from the line and character spacing (as appears to be the case in Schroeder’s (2019) implementation in popEye) or other known properties of the passage, language, or reader. Secondly, our simulations showed that chain does not respond well to slope distortion, performing worse than attach under extreme values. This can be alleviated by a *y* threshold that grows as the reader progresses over the line, as is the case in Hyrskykari’s (2006) sticky lines algorithm.

#### Cluster

The biggest weakness of cluster was its ability to deal with general noise (or, equivalently, tight line spacing). One potential way to improve this would be to utilize the *x* values of the fixations. Unfortunately, it is not simply a case of performing a two-dimensional *k*-means clustering on the *xy* values because this leads to situations where clusters are identified that span multiple lines because they have similar *x* values. However, it might still be possible to utilize the *x*-axis information, perhaps by weighting the two axes differently in some way. Cluster analysis is a very broad topic in data science, and there are likely to be many other candidate algorithms, beyond simple *k*-means clustering, which will be worth investigating.

#### Merge

The core principle of merge is to start with small groups of fixations and gradually build them up into gaze lines, guided by their fit to regression lines. The most extreme version of this algorithm would start with every fixation in an individual group, and the algorithm would consider every sequence in which mergers could be performed (i.e., the entire binary search tree). This would allow the algorithm to explore cases where it is first necessary to make a bad merger in order to make a great merger later on (i.e., it would avoid becoming stuck in local maxima). Such an algorithm would be intractable, however, due to a combinatorial explosion in the number of possible merge sequences. To avoid this, merge uses an initial chain-like strategy to seed the merge process with a reduced set of groups, and it then explores just one possible path through the search tree, selecting only the most promising merger at each step. One way to improve the algorithm, then, would be to use more advanced tree traversal techniques, such as beam search in which several of the most promising mergers are fully explored on each iteration. This would come at the cost of making an already slow algorithm even slower, but it would probably result in better solutions and might also allow for the removal of the thresholds and heuristics.

#### Regress

The main weakness of the regress algorithm is that the *m* regression lines it fits to the data cannot take independent slope or offset values, limiting its ability to handle complex cases, especially those involving shift. Thus, one obvious way to advance the algorithm would be to allow for such independent values. However, even the simplest case of having a single slope parameter, a single standard deviation parameter, and one offset parameter per line of text would result in an objective function with *m* + 2 parameters, which may become difficult or impossible to minimize, especially as the number of lines increases. Another avenue for improving regress would be to try some form of nonlinear regression. In Fig. 9a, for example, we see a case where a gaze line forms a nonlinear arc, which a linear regression line cannot fully capture (cf. Fig. 9h).

#### Segment

The performance of the segment algorithm hinges on its ability to identify the true return sweeps; when it works, it tends to work very well, but when it fails, it does so catastrophically. One way to improve the segment algorithm would therefore be to encode additional heuristics about how to distinguish true return sweeps from normal regressions. For example, a return sweep is not just an extreme movement to the left but also a movement downward by a relatively predictable amount (one line space), ultimately landing near the left edge of the passage. Introducing such heuristics would not be without caveats, however; in the case of downward slope, for example, return sweeps can appear quite flat (see, e.g., the final sweep in Fig. 8a) and would therefore go unnoticed under this change.

#### Split

Like segment, split could also benefit from better sweep detection, as well as better detection of between-line regressions. There are likely to be many ways of approaching this classification problem, but one simple option would be to use both dimensions in the saccade clustering—the return sweeps would then be the cluster of saccades that have large negative change on the *x*-axis, as well as a small positive change on the *y*-axis. More generally, it might be possible to combine the split and segment algorithms, since they are quite closely related computationally. For example, the set of saccades that most resemble return sweeps could first be identified, and then the *m* − 1 most extreme of these could be treated as major segmentation points, allowing for a sequential assignment, while the remainder could be treated as minor segmentation points, allowing for the identification of between-line regressions.

#### Stretch

Our analyses showed that stretch behaves very similarly to regress. This is because they are essentially two variants on the same basic idea: Detect the magnitude of the underlying slope (regress) or shift (stretch) and then reverse it. However, this does not work so well if the underlying calibration error is fluctuating in time or space. One way to improve the method, then, would be to search a more complex transformation space by including rotations and shears, for example, or by applying separate transformations to each quadrant of the text. Additionally, as Eq. 1 makes clear, stretch essentially has the attach algorithm embedded within it, but in principle it should be possible to substitute this with any of the positional algorithms. For example, a stretch-chain algorithm would find a transformation of the fixations that results in minimal change when you apply the chain algorithm.

#### Warp

The primary weakness of warp is that the expected fixation sequence cannot encode unpredictable reading behavior that might be present in the veridical sequence, and there is no feasible way such unpredictability could be encoded. Instead, improving the warp algorithm is likely to involve relaxing DTW’s requirement that matches between sequences increase monotonically, allowing the algorithm to find a mix of global and local sequence alignments. In this respect, the so-called “glocal” alignment algorithms could prove useful (Brudno et al., 2003), as well as many other sequence alignment algorithms that ought to be systematically investigated for the present purposes (e.g., Keogh & Pazzani, 2001; Tomasi et al.,, 2004; Tormene et al.,, 2009; Uchida, 2005). One simpler option—which could also be applied to segment—would be to use attach as a fallback method in cases where a fixation’s revised *y*-axis coordinate is substantially different from its original *y*-axis coordinate. This would deal with cases where the strict sequentiality requirement forces fixations on to lines that are very far from their original positions.

### Improvements on the benchmarking

Aside from improving the algorithms themselves, it would also be useful to produce a much larger, heterogeneous benchmarking dataset, with data contributed from many different laboratories. This would offer more generalizable results and would help us understand how the algorithms respond to specific factors. For example, one useful feature of the dataset we used in this paper is that it includes data from both adults and children on the same passages of text, allowing us to compare how the algorithms respond to these two distinct populations. However, there are many other factors that will ultimately determine how the algorithms behave, such as the layout of the text, the complexity of the reading material, and the peculiarities of the eye-tracker hardware. In addition, there are likely to be important linguistic factors at play: For example, our current set of results may not generalize well to logographic writing systems, such as Chinese, right-to-left scripts, such as Hebrew, orthographically opaque languages, such as English, or agglutinating languages, such as Turkish, where fixation patterns might differ in ways that the algorithms are sensitive to. However, the main difficulty we foresee in creating such a heterogeneous dataset—aside from producing the required manual corrections—would be ensuring it is representative of the kinds of experiments that researchers most typically run, while also diverse enough to capture all relevant factors.

## Conclusions

Our intentions with this paper were twofold. Firstly, we wanted to systematically evaluate the various vertical drift correction algorithms that have been reported in the literature in order to provide guidance to researchers about how they work, when they should be used, and what their limitations are. In this respect, our most important observation was that there is no one killer app; different datasets—and even different trials within a dataset—will require different solutions, so researchers should select their correction method carefully. We hope that the guidance we have provided herein will be helpful in this regard.

Secondly, we wanted to lay a solid foundation for future work on post hoc vertical drift correction by delimiting the core algorithms, providing constraints that future work can operate inside, and offering new perspectives on how drift correction techniques can be improved going forward. In this respect, we have provided basic implementations of the ten algorithms in multiple languages, which can be used as a starting point for building new versions or, indeed, as a comparison group against which entirely new algorithms can be compared. Several of the algorithms are already implemented in the Python package Eyekit (https://jwcarr.github.io/eyekit/) and the R package popEye (https://github.com/sascha2schroeder/popEye), which provide higher level tools for processing and analyzing reading data more generally. In time, we hope that the algorithms might also be implemented in other software packages.

Finally, we have introduced two novel methods in this paper that are distinct from those that have previously been presented. The warp algorithm, in particular, showed great promise and is likely to be especially useful to researchers working on reading development in children. We also hope that connecting the literature on vertical drift to sequence alignment techniques might also open new avenues for future algorithm development.

## Electronic supplementary material

Below is the link to the electronic supplementary material.

(PDF 208 KB)

(PDF 7.74 MB)

## Footnotes

1 In both the cluster and split algorithms, we used the KMeans function from the Python library Scikit-learn (Pedregosa et al., 2011). There are many variations of *k*-means clustering, which we have not systematically compared.

2 In both the regress and stretch algorithms, we used the minimize function from the Python library SciPy (Virtanen et al., 2020). We have not systematically compared the choice of optimizer settings.

3 Specifically, the first fixation must be mapped to (at least) the first word; the last fixation must be mapped to (at least) the last word; every other fixation must be mapped to at least one word; and, if fixation *i* is mapped to word *j*, then fixation *i* + 1 must be mapped to word(s) ≥ *j*. And vice versa for the mapping from words to fixations.

4 For example, regress appears to have worse performance on shift compared to slope; however, if we had simulated a narrower range of shift values, the results might have led us to the opposite conclusion.

5 For example, if the standard deviation bounds of regress are widened, it may be possible to improve performance on noise, but the algorithm will be less capable of dealing with slope.

6 Unlike compare and segment, even if split misidentifies a regression as a return sweep, it will still be able to map the resulting gaze line to the appropriate text line because it assigns based on position rather than order.

7 This concurs with the 60% accuracy reported by Lima Sanches et al., (2015, p. 1231). Yamaya et al., (2017, p. 104), who describe a slightly more complex method with better sweep detection, report accuracy of 87%, which is still quite low compared to the other results obtained in this paper.

8 Since compare was highly divergent from all other algorithms due to its poor performance, it is not included in this analysis.
