# Supplementary material for: Algorithms for the automated correction of vertical drift in eye-tracking data
Source: Behav Res Methods. 2021 Jun 22;54(1):287–310. doi: 10.3758/s13428-021-01554-0 (PMC8863743; doi:10.3758/s13428-021-01554-0)
Supplement: Supplementary file 1 — (PDF 208 KB) [file 13428_2021_1554_MOESM1_ESM.pdf]

Algorithms for the Automated Correction of Vertical Drift in Eye Tracking Data

Supplementary Item 1: Pseudocode

For reference, we present the algorithms here in a pseudocode that should be clear to programmers of any high-level scientific programming language. Matlab/Octave, Python, and R implementations may be found at https://github.com/jwcarr/drift or https://osf.io/7srkg/. We have generally emphasized readability over optimization, and we make very minimal assumptions about the input and output. Most of the algorithms take two inputs: `fixation_XY`, an array of size $n \times 2$ representing the xy positions of n fixations, and `line_Y`, an array of length m representing the y positions of the m lines of text. Some algorithms take slightly different input or additional arguments as detailed below. All algorithms return a modified `fixation_XY` as output, in which only the y values have been adjusted.

Attach

```
function attach(fixation_XY, line_Y)
|   n = length(fixation_XY)
|   for fixation_i in 1 : n
|   |   fixation_y = fixation_XY[fixation_i, 2]
|   |   line_i = argmin(abs(line_Y - fixation_y))
|   |   fixation_XY[fixation_i, 2] = line_Y[line_i]
|   return fixation_XY
```

Chain

`chain` takes two additional arguments, `x_thresh` and `y_thresh`, which determine how much change is required on the x- or y-axis to start a new chain of fixations.

```
function chain(fixation_XY, line_Y, x_thresh=192, y_thresh=32)
|   n = length(fixation_XY)
|   dist_X = abs(diff(fixation_XY[:, 1]))
|   dist_Y = abs(diff(fixation_XY[:, 2]))
|   end_chain_indices = where(dist_X > x_thresh or dist_Y > y_thresh)
|   end_chain_indices = append(end_chain_indices, n)
|   start_of_chain = 1
|   for end_of_chain in end_chain_indices
|   |   mean_y = mean(fixation_XY[start_of_chain:end_of_chain, 2])
|   |   line_i = argmin(abs(line_Y - mean_y))
|   |   fixation_XY[start_of_chain:end_of_chain, 2] = line_Y[line_i]
|   |   start_of_chain = end_of_chain + 1
|   return fixation_XY
```

Cluster

`cluster` calls on one external function, `kmeans`, which returns `clusters`, an array of length n that gives the cluster index of each fixation, and `centers`, an array of length m that gives the mean y value of each cluster.

```
function cluster(fixation_XY, line_Y)
    n = length(fixation_XY)
    m = length(line_Y)
    fixation_Y = fixation_XY[:, 2]
    clusters, centers = kmeans(fixation_Y, m)
    ordered_cluster_indices = argsort(centers)
    for fixation_i in 1 : n
        cluster_i = clusters[fixation_i]
        line_i = where(ordered_cluster_indices == cluster_i)
        fixation_XY[fixation_i, 2] = line_Y[line_i]
    return fixation_XY
```

Compare

Instead of `line_Y`, `compare` takes `word_XY` as its second argument, an array representing the xy positions of the centers of all words in the order in which they are expected to be read. It takes two additional arguments: `x_thresh`, which specifies the threshold for considering backward saccades to be return sweeps, and `n_nearest_lines`, which determines how many neighboring text lines a gaze line will be compared to. `compare` calls on one external function, `dynamic_time_warping`, which returns the DTW cost between a gaze line and text line.

```
function compare(fixation_XY, word_XY, x_thresh=512, n_nearest_lines=3)
    n = length(fixation_XY)
    line_Y = unique(word_XY[:, 2])
    diff_X = diff(fixation_XY[:, 1])
    end_line_indices = where(diff_X < -x_thresh)
    end_line_indices = append(end_line_indices, n)
    start_of_line = 1
    for end_of_line in end_line_indices
        gaze_line = fixation_XY[start_of_line:end_of_line]
        mean_y = mean(gaze_line[:, 2])
        lines_ordered_by_proximity = argsort(abs(line_Y - mean_y))
        nearest_line_I = lines_ordered_by_proximity[1:n_nearest_lines]
        line_costs = zeros(n_nearest_lines)
        for candidate_i in 1 : n_nearest_lines
            candidate_line_i = nearest_line_I[candidate_i]
            candidate_line_y = line_Y[candidate_line_i]
            text_line = word_XY[word_XY[:, 2] == candidate_line_y]
            cost, _ = dynamic_time_warping(gaze_line[:, 1], text_line[:, 1])
            line_costs[candidate_i] = cost
        line_i = nearest_line_I[argmin(line_costs)]
        fixation_XY[start_of_line:end_of_line, 2] = line_Y[line_i]
        start_of_line = end_of_line + 1
    return fixation_XY
```

Merge

`merge` takes three additional arguments: `y_thresh` determines how much change is required on the y-axis to start a new sequence of progressive fixations; `g_thresh` determines the maximum absolute gradient of the fit regression lines; and `e_thresh` determines the maximum regression error. `merge` calls on one external function, `linear_model`, which fits a regression line to a candidate set of fixations and returns `g`, the absolute gradient, and `e`, the regression error (RMSD). The global variable `phases` defines three parameters per phase of the merge process: the minimum number of fixations in the first candidate sequence; the minimum number of fixations in the second candidate sequence; and a Boolean that removes the gradient and error constraints (this should be `TRUE` in the final phase to ensure that the number of sequences can be reduced to m).

```
phases = [[3, 3, FALSE], [1, 3, FALSE], [1, 1, FALSE], [1, 1, TRUE]]

function merge(fixation_XY, line_Y, y_thresh=32, g_thresh=0.1, e_thresh=20)
    n = length(fixation_XY)
    m = length(line_Y)
    diff_X = diff(fixation_XY[:, 1])
    dist_Y = abs(diff(fixation_XY[:, 2]))
    sequence_boundaries = where(diff_X < 0 or dist_Y > y_thresh)
    sequence_boundaries = append(sequence_boundaries, n)
    sequences = []
    start_of_sequence = 1
    for end_of_sequence in sequence_boundaries
        sequence = start_of_sequence : end_of_sequence
        sequences = append(sequences, sequence)
        start_of_sequence = end_of_sequence + 1
    for min_i, min_j, no_constraints in phases
        while length(sequences) > m
            best_merger = NONE
            best_error = INFINITY
            for i in 1 : length(sequences) - 1
                if length(sequences[i]) < min_i
                    next # first sequence too short, skip to next i
                for j in i+1 : length(sequences)
                    if length(sequences[j]) < min_j
                        next # second sequence too short, skip to next j
                    candidate_sequence = concatenate(sequences[i], sequences[j])
                    g, e = linear_model(fixation_XY[candidate_sequence])
                    if no_constraints == TRUE or (g < g_thresh and e < e_thresh)
                        if e < best_error
                            best_merger = [i, j]
                            best_error = e
            if best_merger == NONE
                break # no possible mergers, break while and move to next phase
            i, j = best_merger
            combined_sequence = concatenate(sequences[i], sequences[j])
            sequences = append(sequences, combined_sequence)
            delete sequences[j], sequences[i]
    mean_Y = zeros(length(sequences))
    for sequence_i in 1 : length(sequences)
        mean_Y[sequence_i] = mean(fixation_XY[sequences[sequence_i], 2])
    ordered_sequence_indices = argsort(mean_Y)
    for sequence_i in 1 : length(sequences)
        line_i = where(ordered_sequence_indices == sequence_i)
        fixation_XY[sequences[sequence_i], 2] = line_Y[line_i]
    return fixation_XY
```

Regress

`regress` takes three additional arguments, `K`, `O`, and `S`, which give the lower and upper bounds of the slope, offset, and standard deviation. `regress` calls on one external function, `minimize`, which minimizes the objective function `fit_lines`. The `fit_lines` function is nested inside `regress` so that it inherits its lexical scope.

```
function regress(fixation_XY, line_Y, K=[-0.1,0.1], O=[-50,50], S=[1,20])
    n = length(fixation_XY)
    m = length(line_Y)

    function fit_lines(params, return_line_assignments=FALSE)
        density = matrix(n, m)
        k = K[1] + (K[2] - K[1]) * cdf(params[1])
        o = O[1] + (O[2] - O[1]) * cdf(params[2])
        s = S[1] + (S[2] - S[1]) * cdf(params[3])
        predicted_Y_from_slope = fixation_XY[:, 1] * k
        line_Y_plus_offset = line_Y + o
        for line_i in 1 : m
            fit_Y = predicted_Y_from_slope + line_Y_plus_offset[line_i]
            density[:, line_i] = logpdf(fixation_XY[:, 2], fit_Y, s)
        if return_line_assignments == TRUE
            return argmax(density, axis=2)
        return -sum(max(density, axis=2))

    initial_params = [0, 0, 0]
    best_params = minimize(fit_lines, initial_params)
    line_assignments = fit_lines(best_params, TRUE)
    for fixation_i in 1 : n
        line_i = line_assignments[fixation_i]
        fixation_XY[fixation_i, 2] = line_Y[line_i]
    return fixation_XY
```

Segment

```
function segment(fixation_XY, line_Y)
    n = length(fixation_XY)
    m = length(line_Y)
    diff_X = diff(fixation_XY[:, 1])
    saccades_ordered_by_length = argsort(diff_X)
    line_change_indices = saccades_ordered_by_length[1:m-1]
    current_line_i = 1
    for fixation_i in 1 : n
        fixation_XY[fixation_i, 2] = line_Y[current_line_i]
        if fixation_i is in line_change_indices
            current_line_i = current_line_i + 1
    return fixation_XY
```

Split

`split` calls on one external function, `kmeans`, which returns `clusters`, an array of length $n-1$ that gives the cluster index of each saccade, and `centers`, an array of length 2 that gives the mean saccade length of each cluster. Whichever cluster has the smaller (i.e., more negative) mean saccade length is assumed to be the cluster that contains the return sweeps.

```
function split(fixation_XY, line_Y)
    n = length(fixation_XY)
    diff_X = diff(fixation_XY[:, 1])
    clusters, centers = kmeans(diff_X, 2)
    sweep_marker = argmin(centers)
    end_line_indices = where(clusters == sweep_marker)
    end_line_indices = append(end_line_indices, n)
    start_of_line = 1
    for end_of_line in end_line_indices
        mean_y = mean(fixation_XY[start_of_line:end_of_line, 2])
        line_i = argmin(abs(line_Y - mean_y))
        fixation_XY[start_of_line:end_of_line, 2] = line_Y[line_i]
        start_of_line = end_of_line + 1
    return fixation_XY
```

Stretch

`stretch` takes two additional arguments, `S` and `O`, which give the lower and upper bounds of the vertical scaling factor and vertical offset. `stretch` calls on one external function, `minimize`, which minimizes the objective function `fit_lines`. The `fit_lines` function is nested inside `stretch` so that it inherits its lexical scope.

```
function stretch(fixation_XY, line_Y, S=[0.9,1.1], O=[-50,50])
    n = length(fixation_XY)
    fixation_Y = fixation_XY[:, 2]

    function fit_lines(params, return_correction=FALSE)
        candidate_Y = fixation_Y * params[1] + params[2]
        corrected_Y = zeros(n)
        for fixation_i in 1 : n
            line_i = argmin(abs(line_Y - candidate_Y[fixation_i]))
            corrected_Y[fixation_i] = line_Y[line_i]
        if return_correction == TRUE
            return corrected_Y
        return sum(abs(candidate_Y - corrected_Y))

    initial_params = [1, 0]
    l_bounds = [S[1], O[1]]
    u_bounds = [S[2], O[2]]
    best_params = minimize(fit_lines, initial_params, l_bounds, u_bounds)
    fixation_XY[, 2] = fit_lines(best_params, return_correction=TRUE)
    return fixation_XY
```

Warp

Instead of `line_Y`, `warp` takes `word_XY` as its second argument: an array representing the *xy* center positions of all words in the order in which they are expected to be read. `warp` calls on one external function, `dynamic_time_warping`, which returns the warping path, a list-of-lists structure that records which words are mapped to each fixation.

```
function warp(fixation_XY, word_XY)
    n = length(fixation_XY)
    _, path = dynamic_time_warping(fixation_XY, word_XY)
    for fixation_i in 1 : n
        words_mapped_to_fixation_i = path[fixation_i]
        candidate_Y = word_XY[words_mapped_to_fixation_i, 2]
        fixation_XY[fixation_i, 2] = mode(candidate_Y)
    return fixation_XY
```
