# Supplementary material for: Algorithms for the automated correction of vertical drift in eye-tracking data
Source: Behav Res Methods. 2021 Jun 22;54(1):287–310. doi: 10.3758/s13428-021-01554-0 (PMC8863743; doi:10.3758/s13428-021-01554-0)
Supplement: Supplementary file 2 — (PDF 7.74 MB) [file 13428_2021_1554_MOESM2_ESM.pdf]

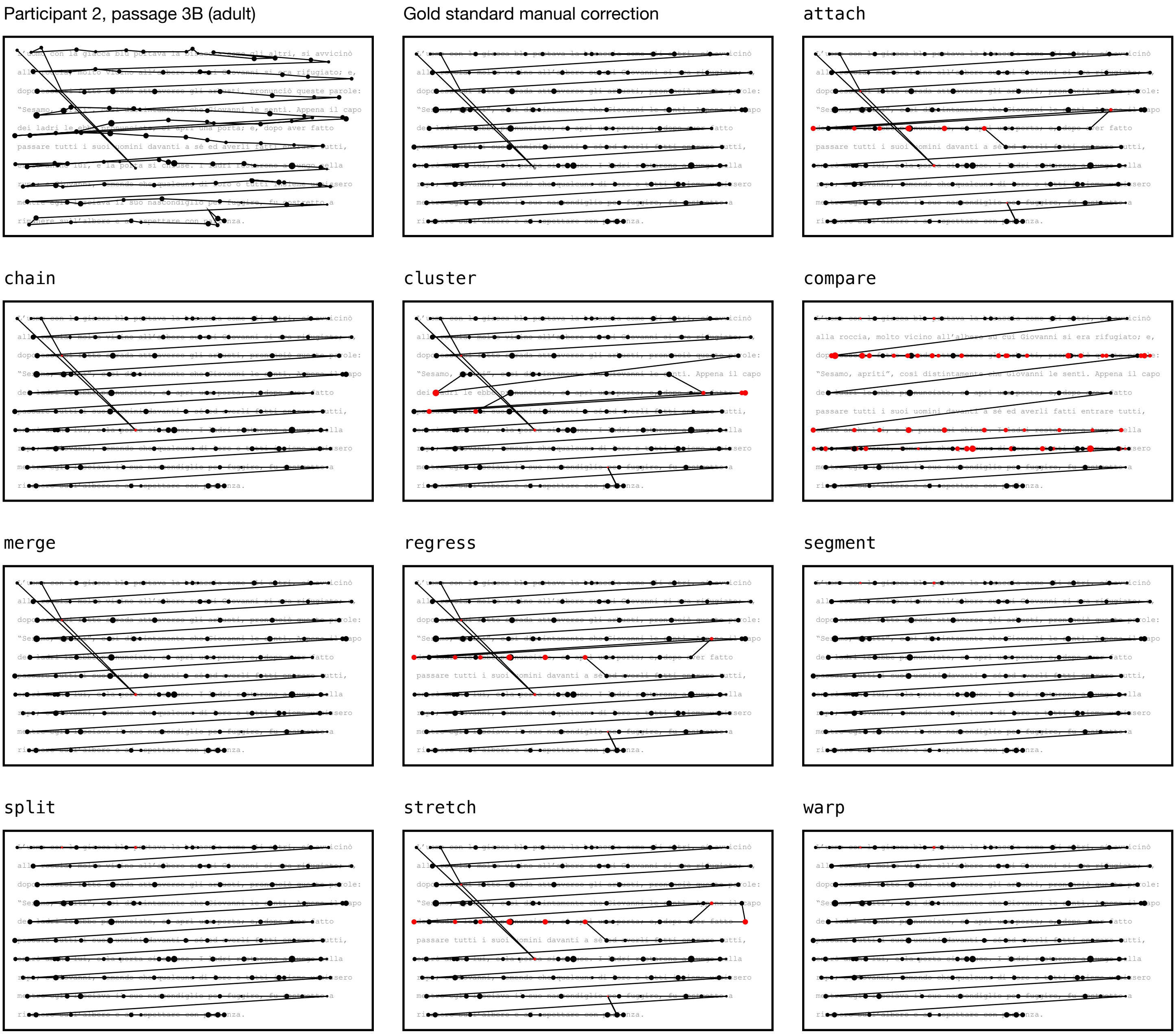
Participant 2, passage 3B (adult)
Gold standard manual correction
attach
chain
cluster
compare
merge
regress
segment
split
stretch
warp

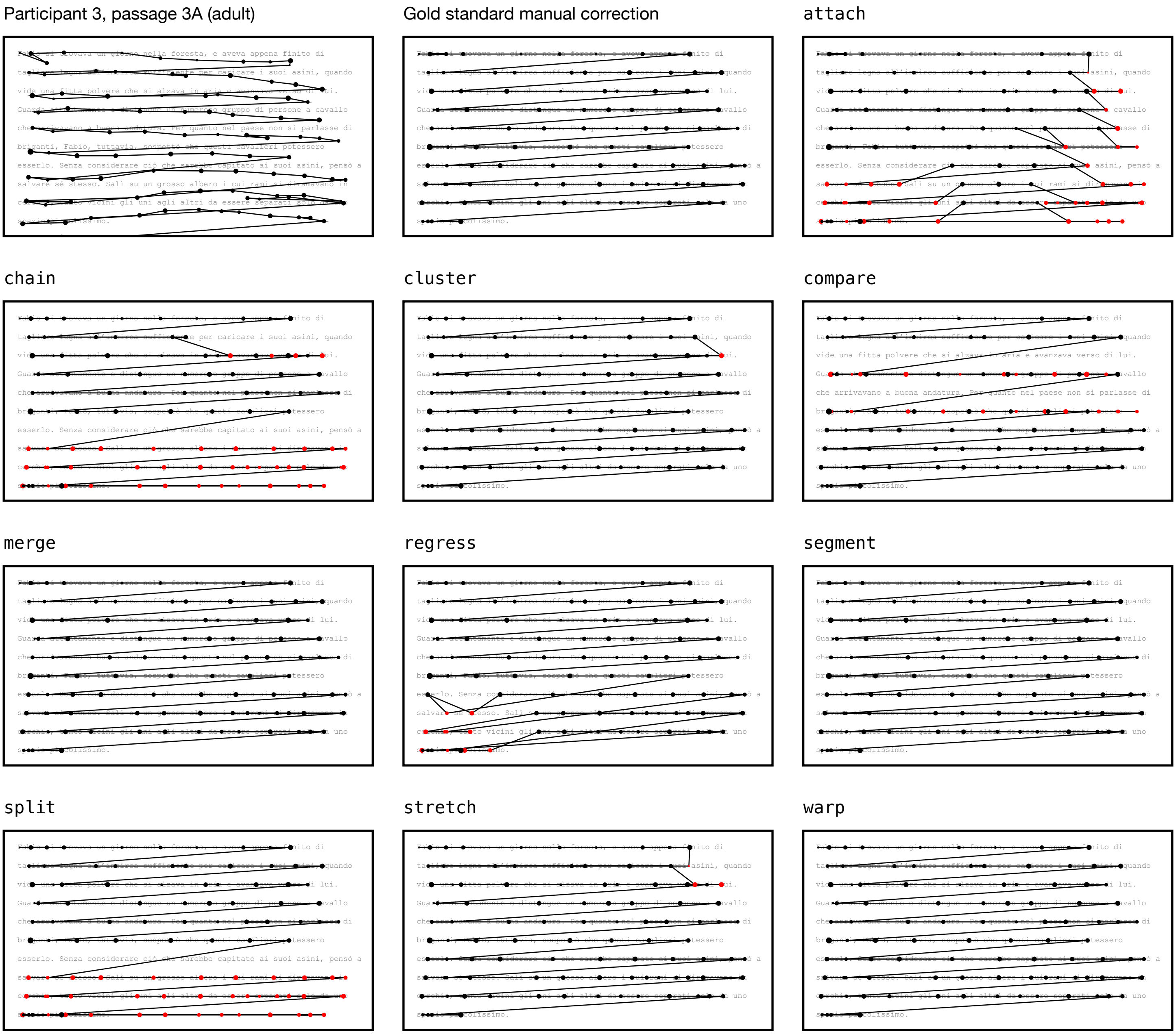
Participant 3, passage 3A (adult)
Gold standard manual correction
attach
chain
cluster
compare
merge
regress
segment
split
stretch
warp

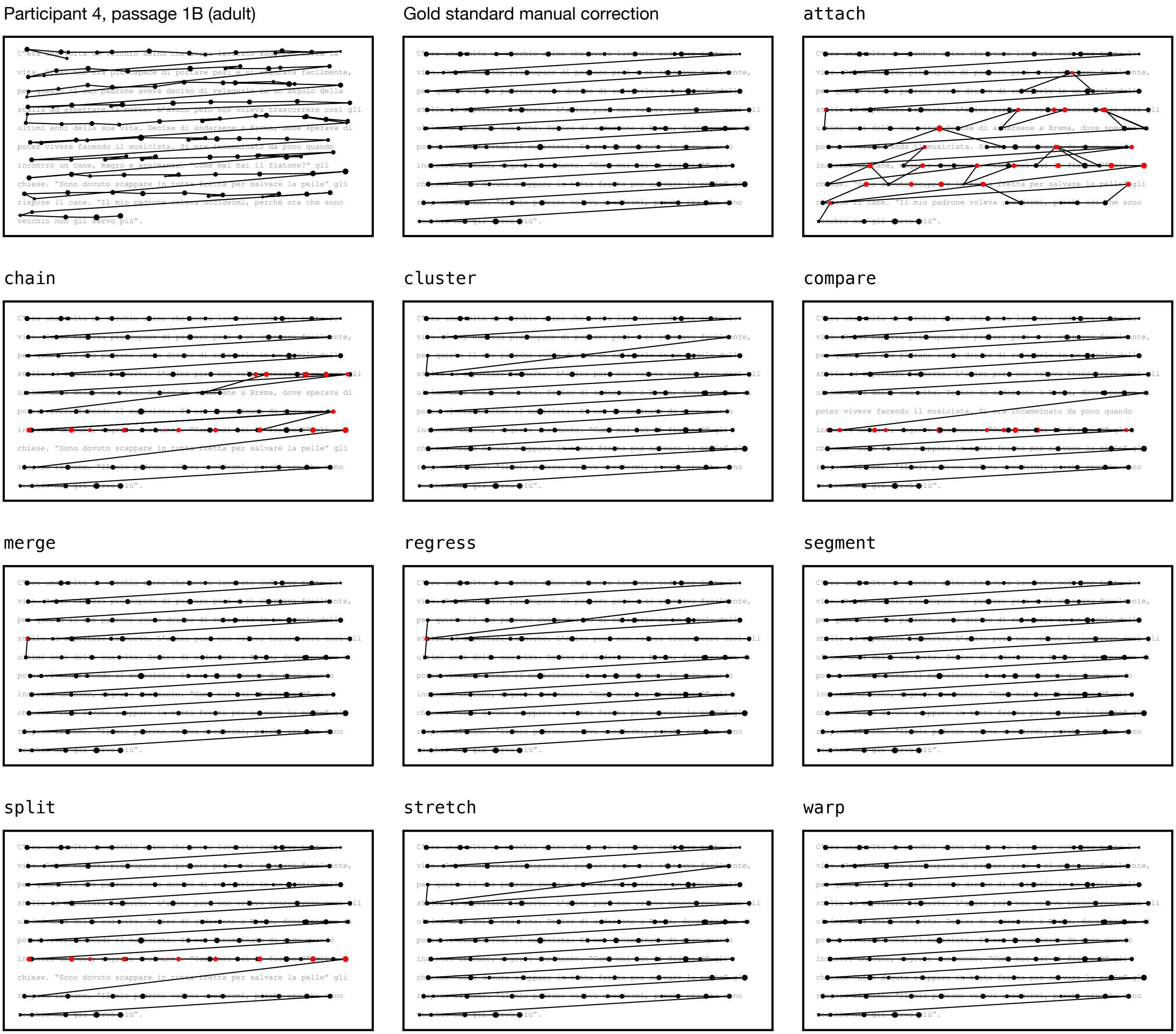
Participant 4, passage 1B (adult)
Gold standard manual correction
attach
chain
cluster
compare
merge
regress
segment
split
stretch
warp

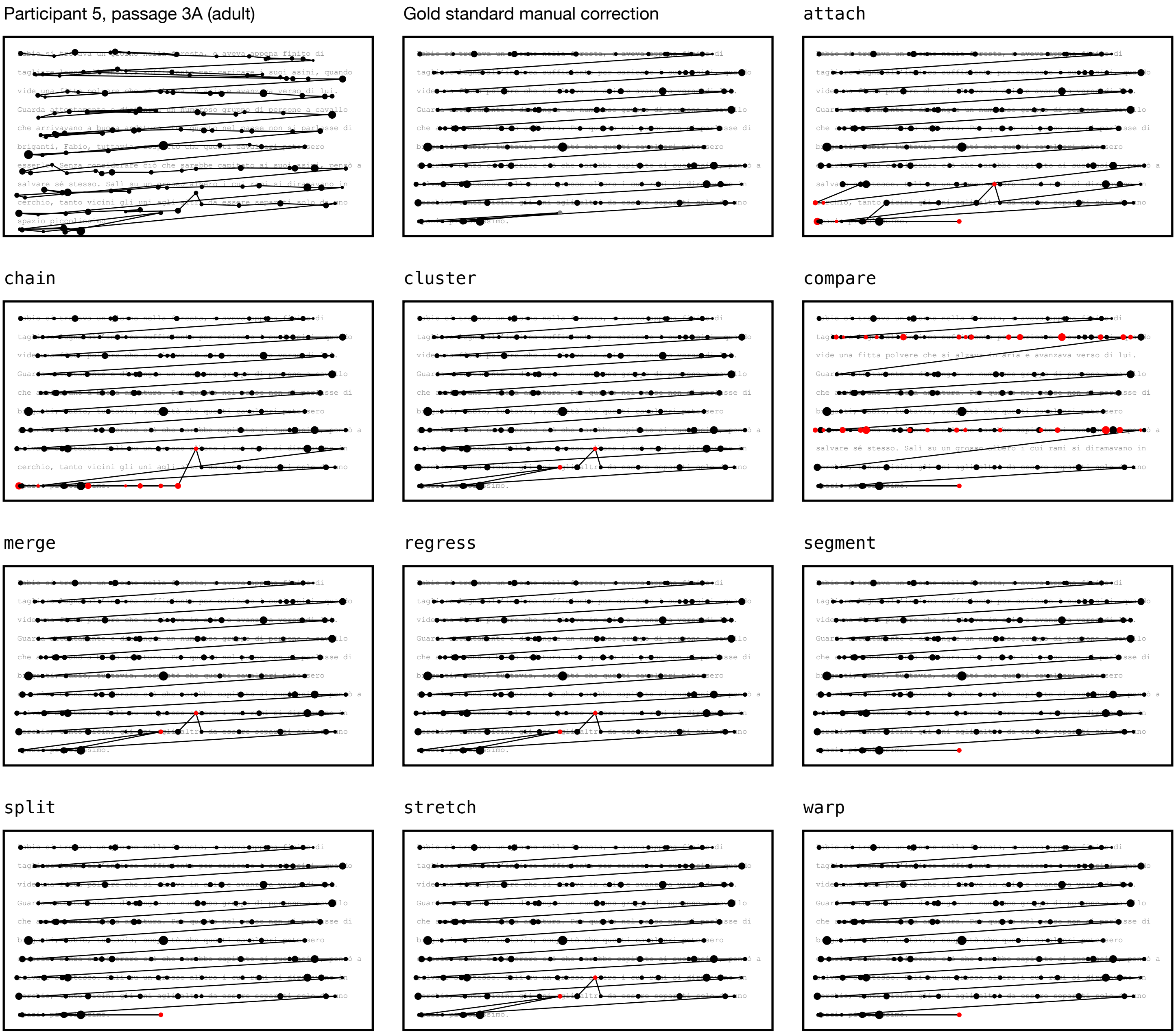
Participant 5, passage 3A (adult)
Gold standard manual correction
attach
chain
cluster
compare
merge
regress
segment
split
stretch
warp

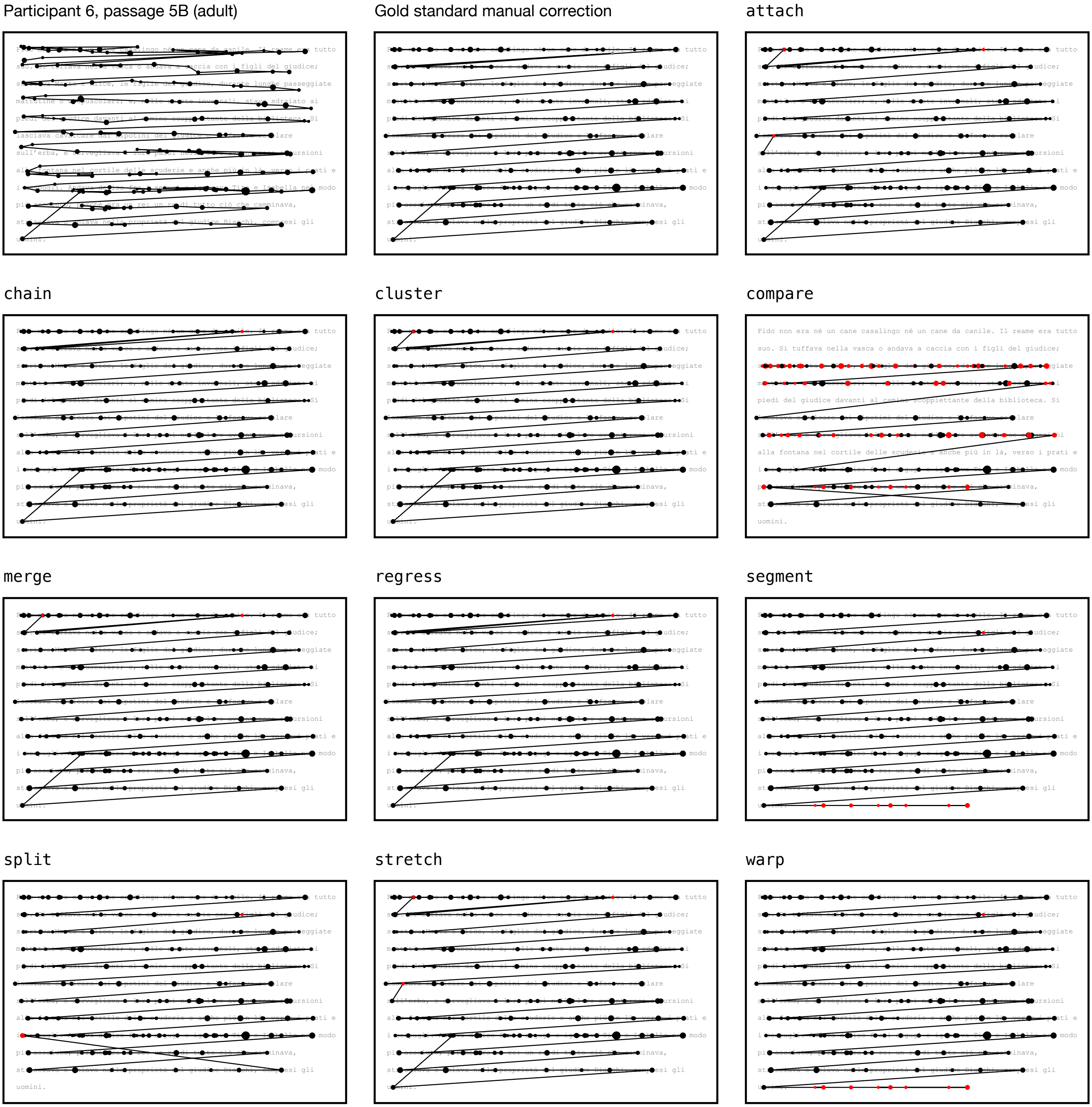
Participant 6, passage 5B (adult)
Gold standard manual correction
attach
chain
cluster
compare
merge
regress
segment
split
stretch
warp

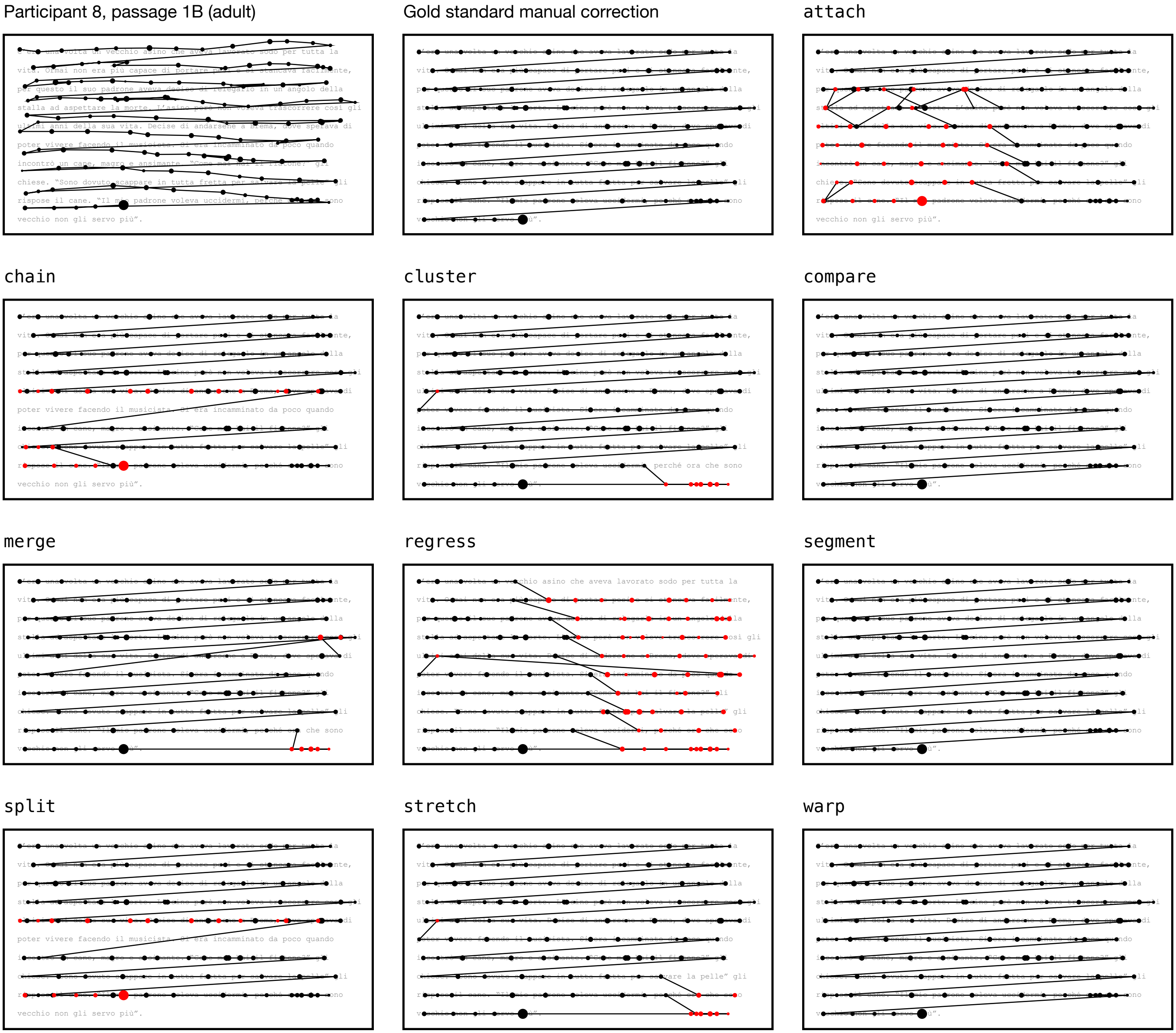

Participant 8, passage 1B (adult)
Gold standard manual correction
attach
chain
cluster
compare
merge
regress
segment
split
stretch
warp

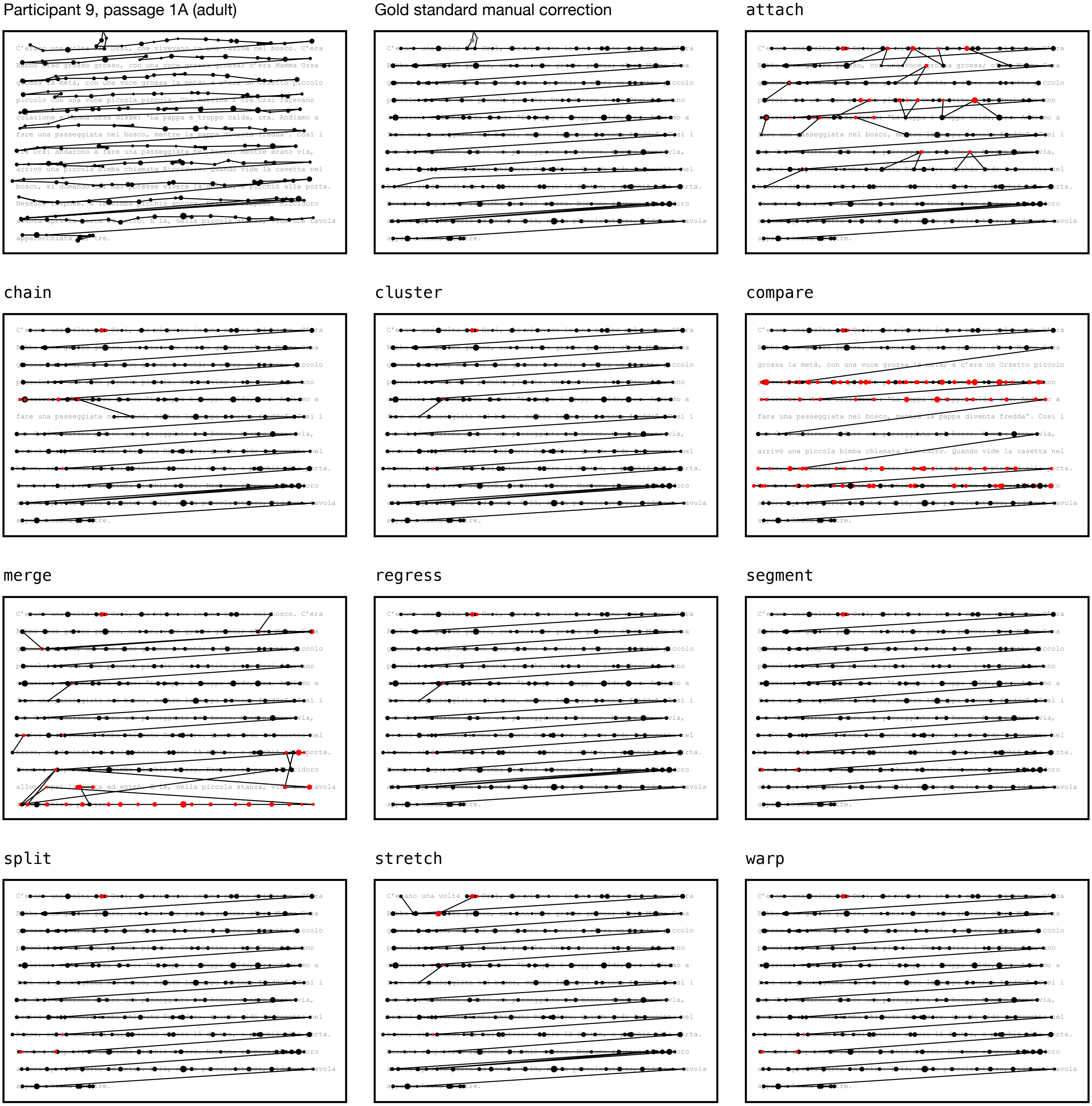

Participant 9, passage 1A (adult)
Gold standard manual correction
attach
chain
cluster
compare
merge
regress
segment
split
stretch
warp

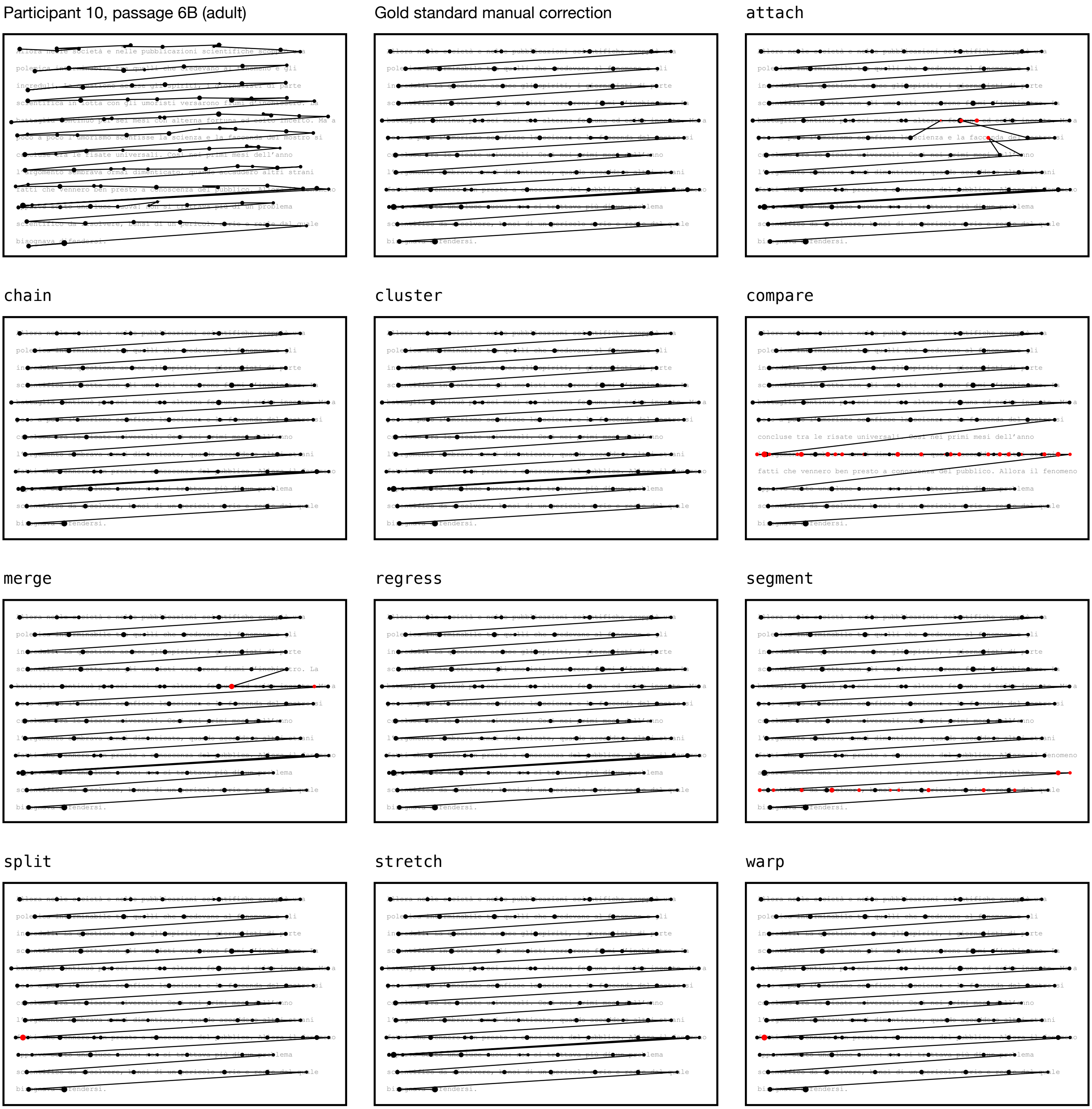

Participant 10, passage 6B (adult)
Gold standard manual correction
attach
chain
cluster
compare
merge
regress
segment
split
stretch
warp

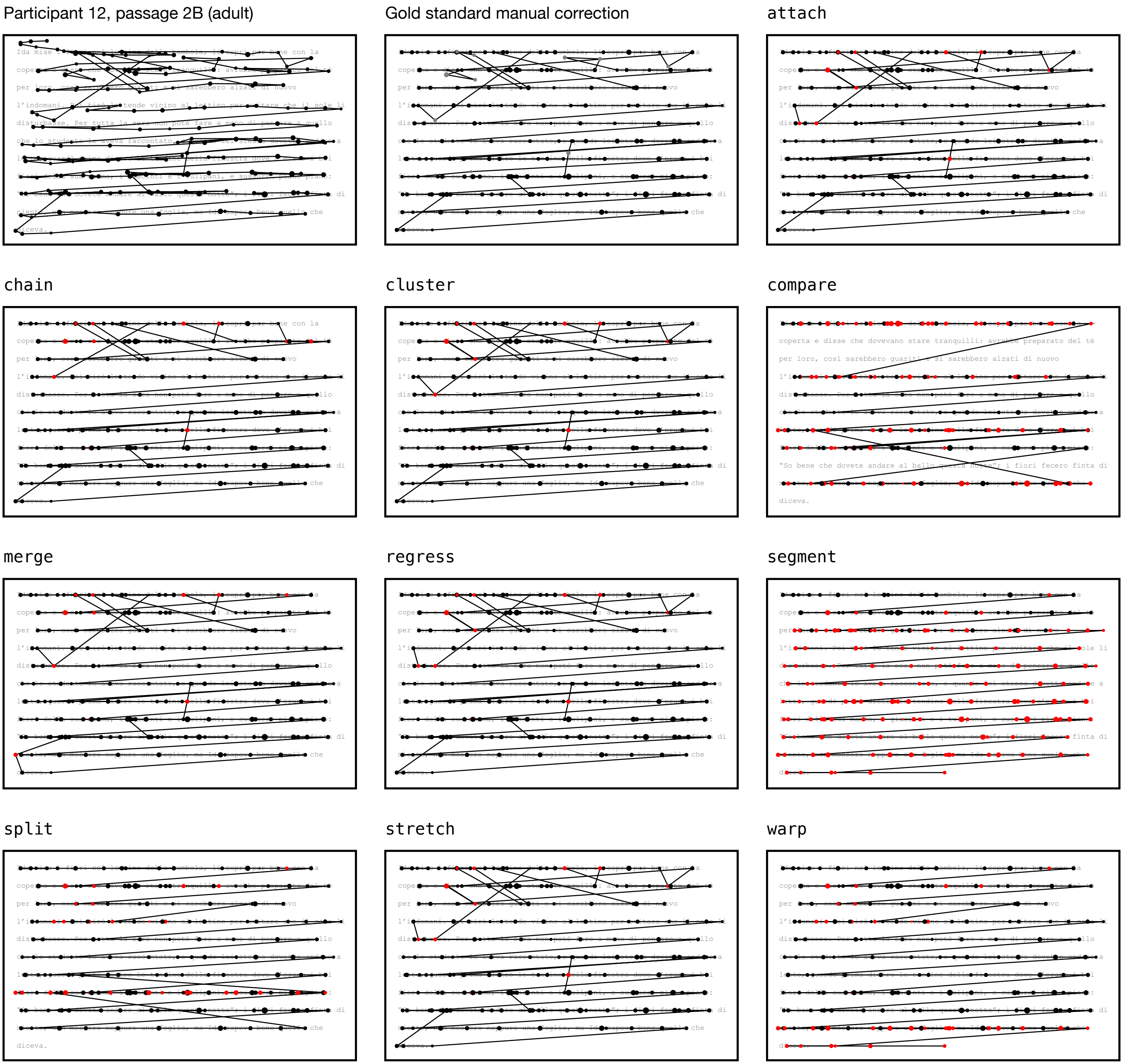
Participant 12, passage 2B (adult)
Gold standard manual correction
attach
chain
cluster
compare
merge
regress
segment
split
stretch
warp

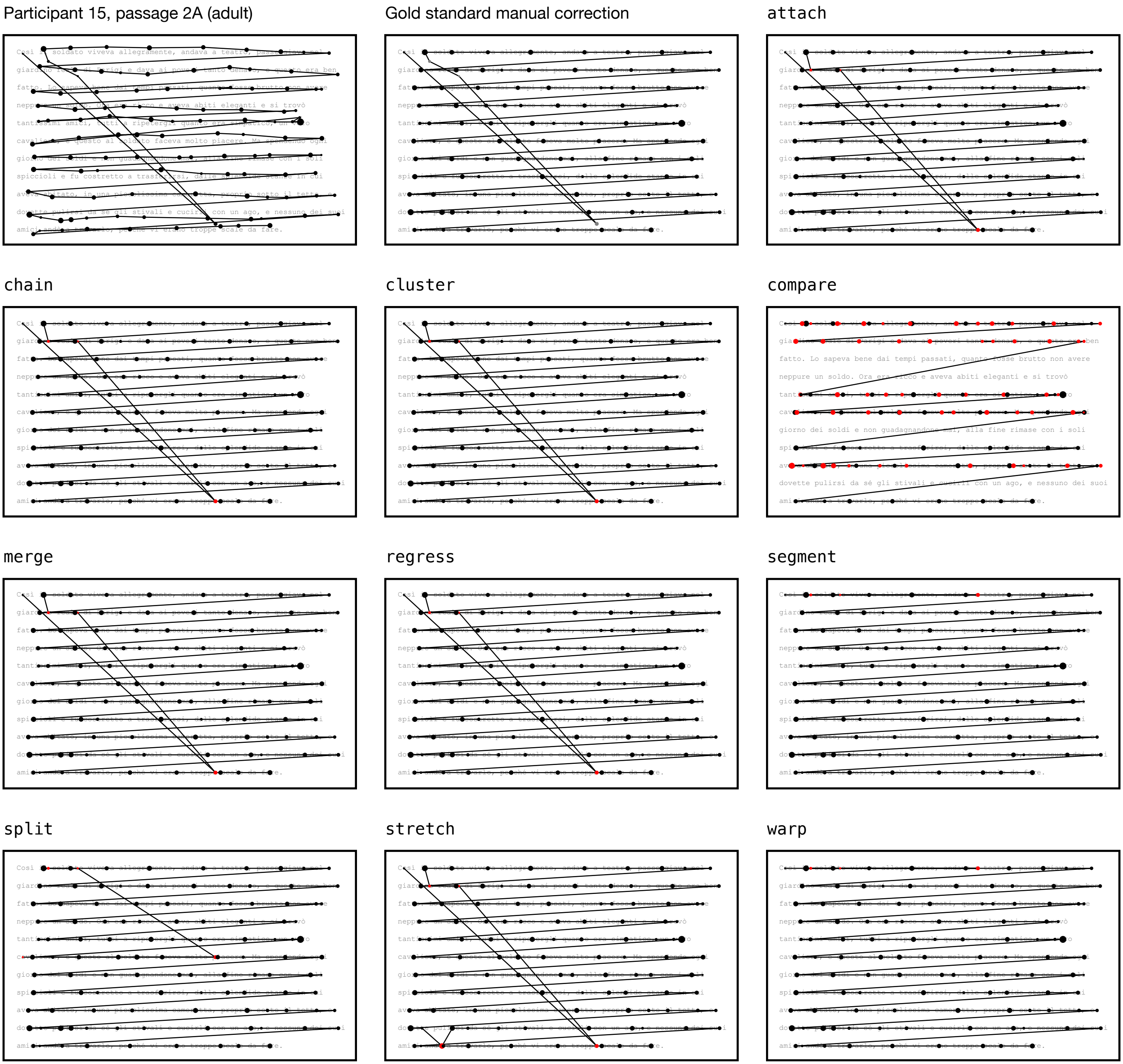

Participant 15, passage 2A (adult)
Gold standard manual correction
attach
chain
cluster
compare
merge
regress
segment
split
stretch
warp

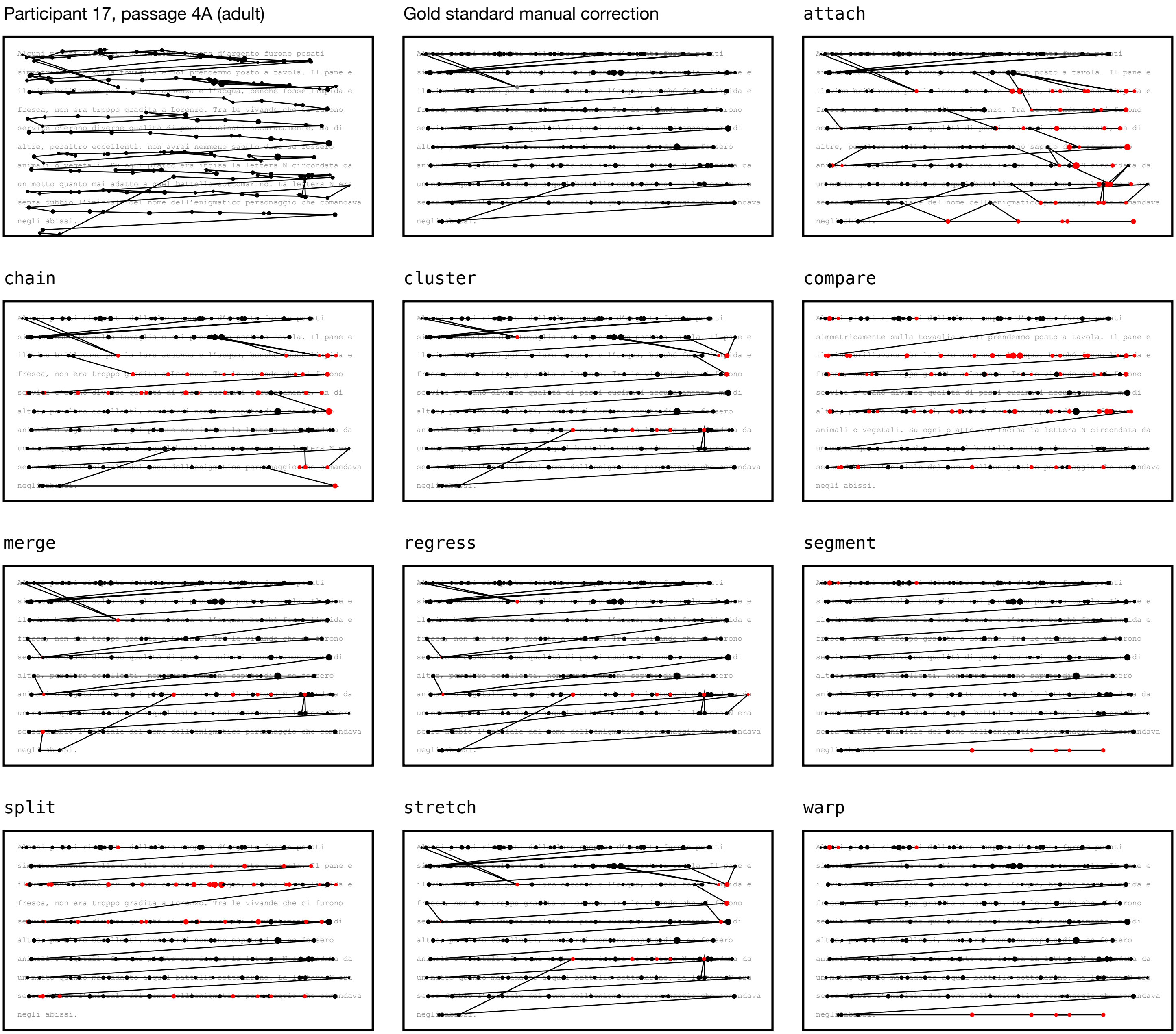

Participant 17, passage 4A (adult)
Gold standard manual correction
attach
chain
cluster
compare
merge
regress
segment
split
stretch
warp

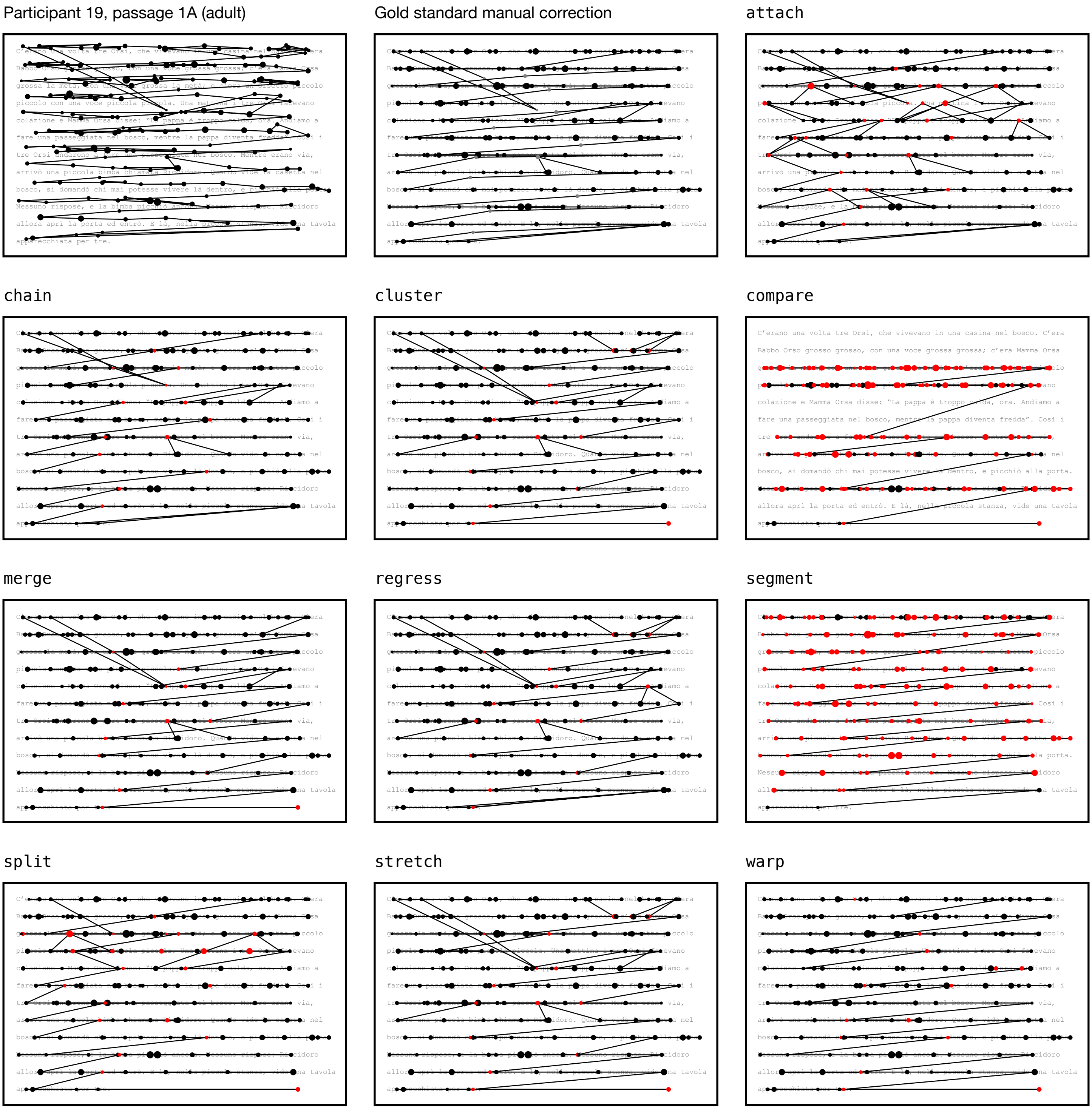
Participant 19, passage 1A (adult)
Gold standard manual correction
attach
chain
cluster
compare
merge
regress
segment
split
stretch
warp

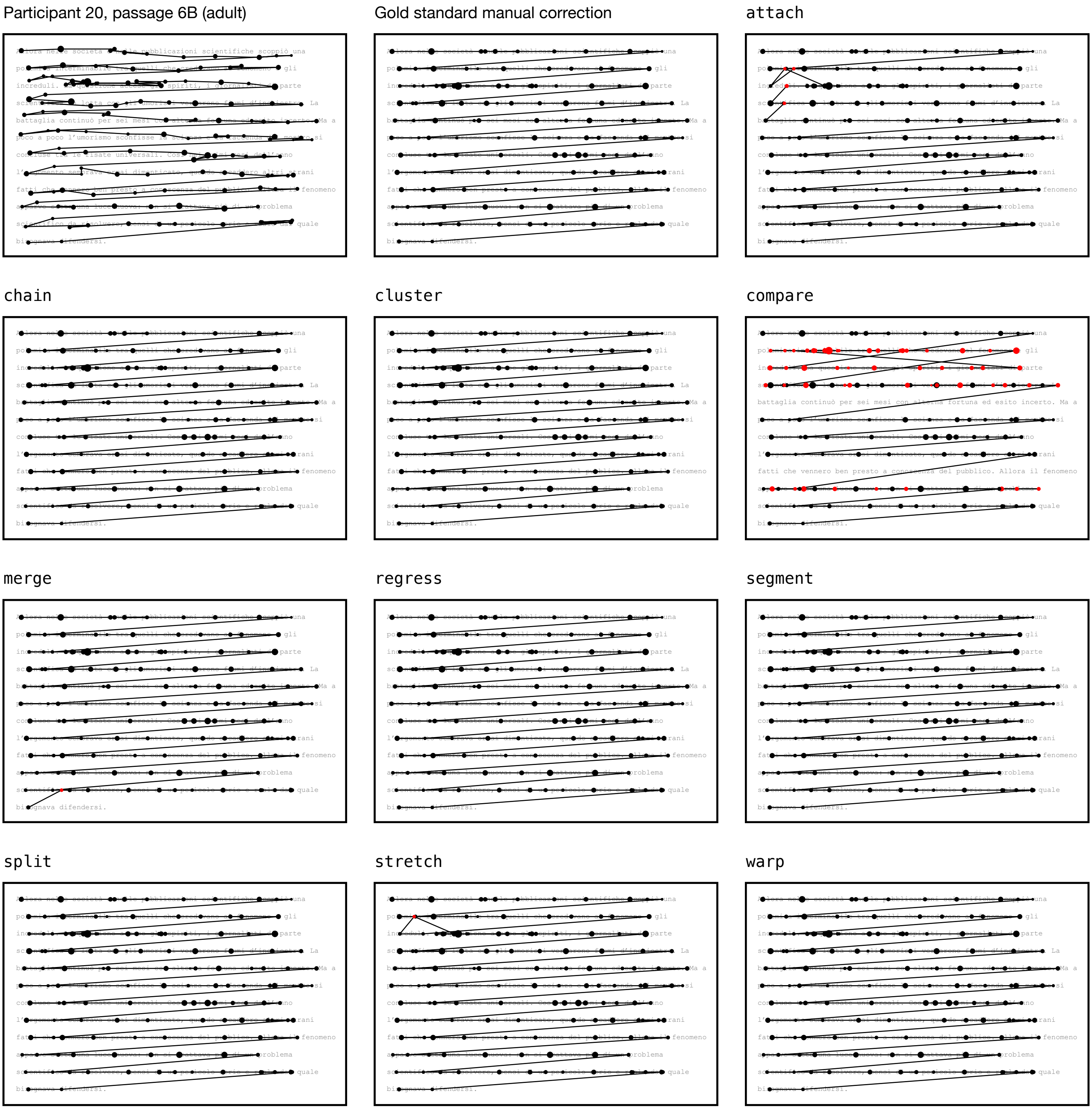
Participant 20, passage 6B (adult)
Gold standard manual correction
attach
chain
cluster
compare
merge
regress
segment
split
stretch
warp

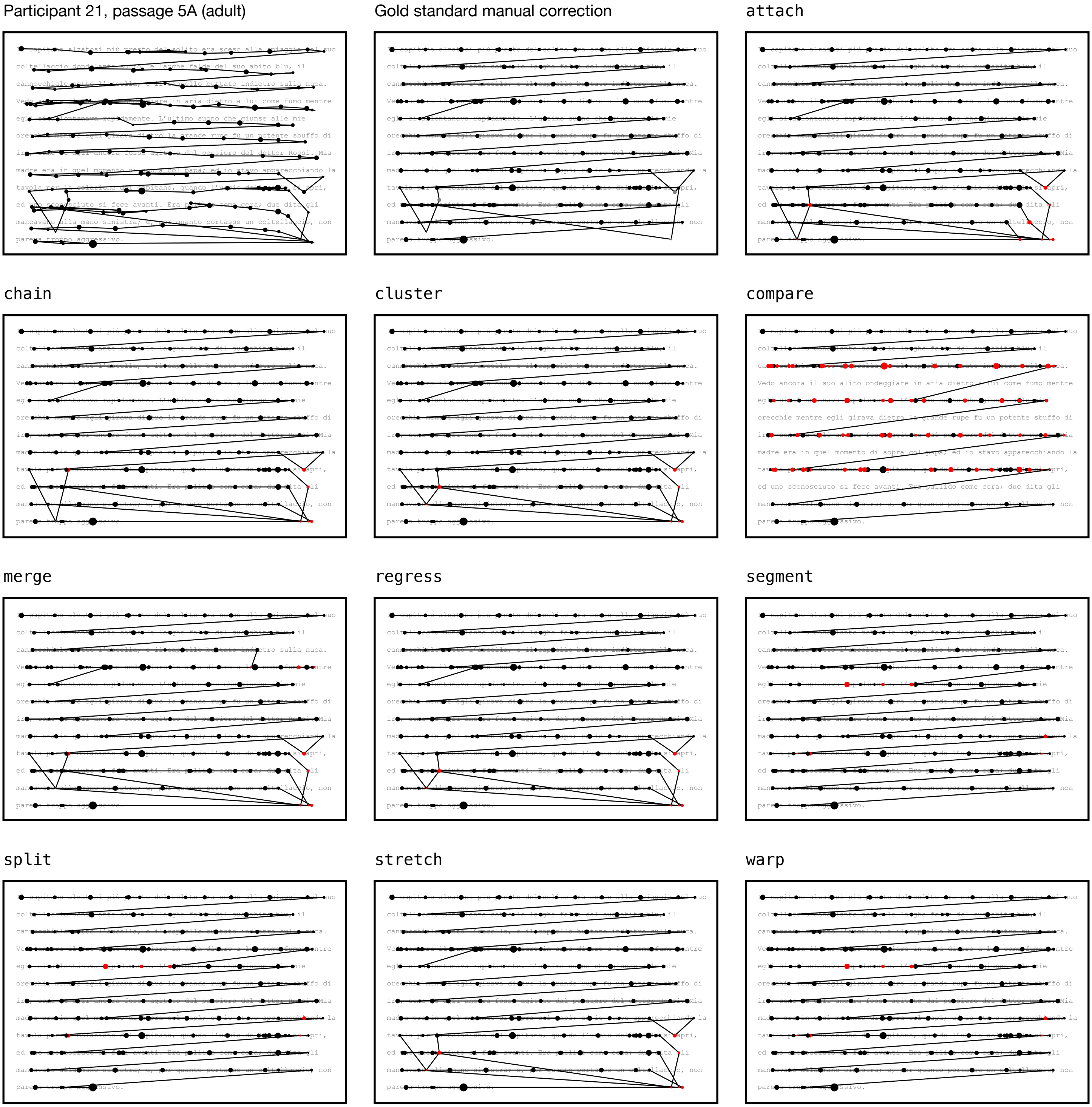
Participant 21, passage 5A (adult)
Gold standard manual correction
attach
chain
cluster
compare
merge
regress
segment
split
stretch
warp

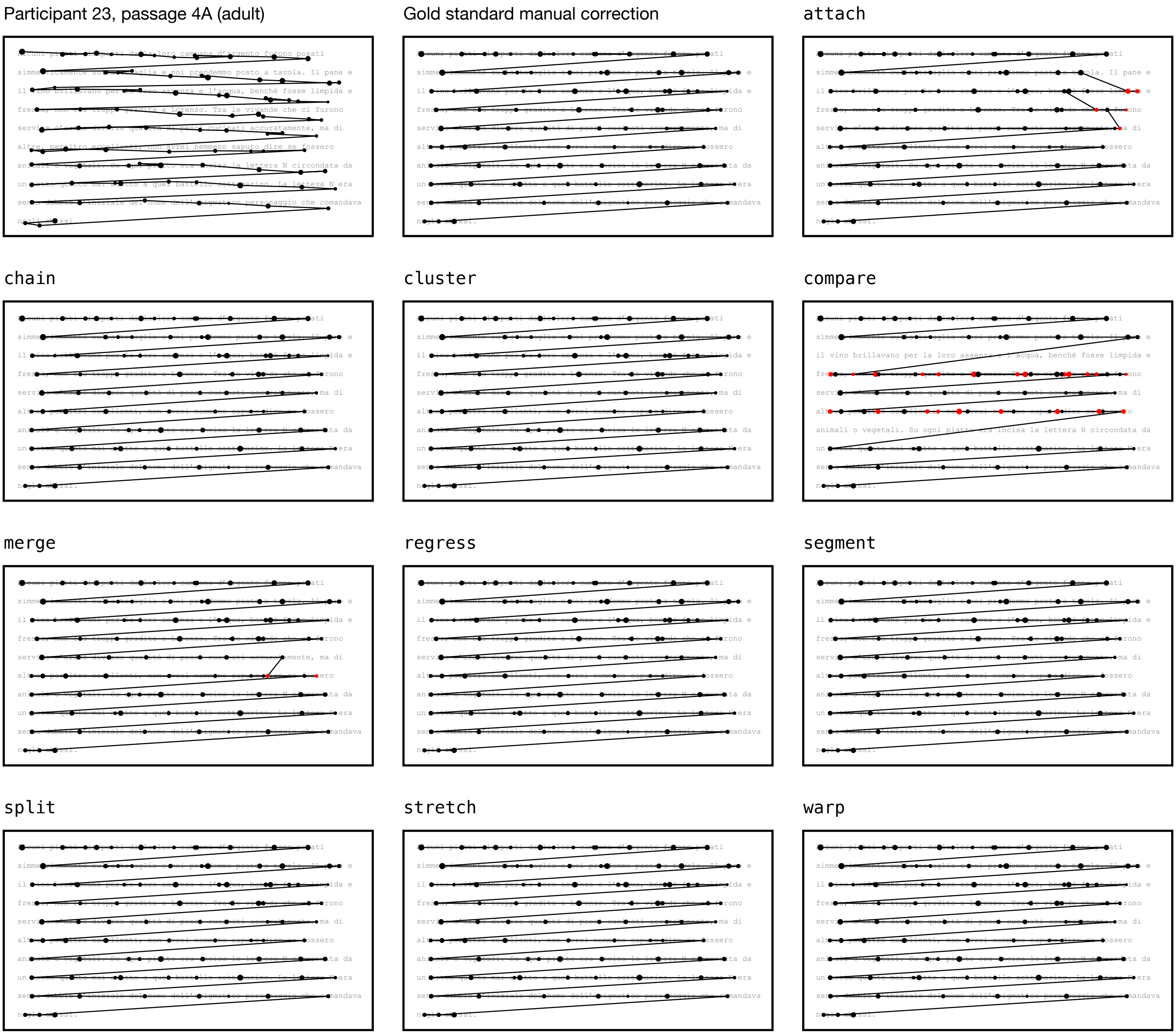
Participant 23, passage 4A (adult)
Gold standard manual correction
attach
chain
cluster
compare
merge
regress
segment
split
stretch
warp

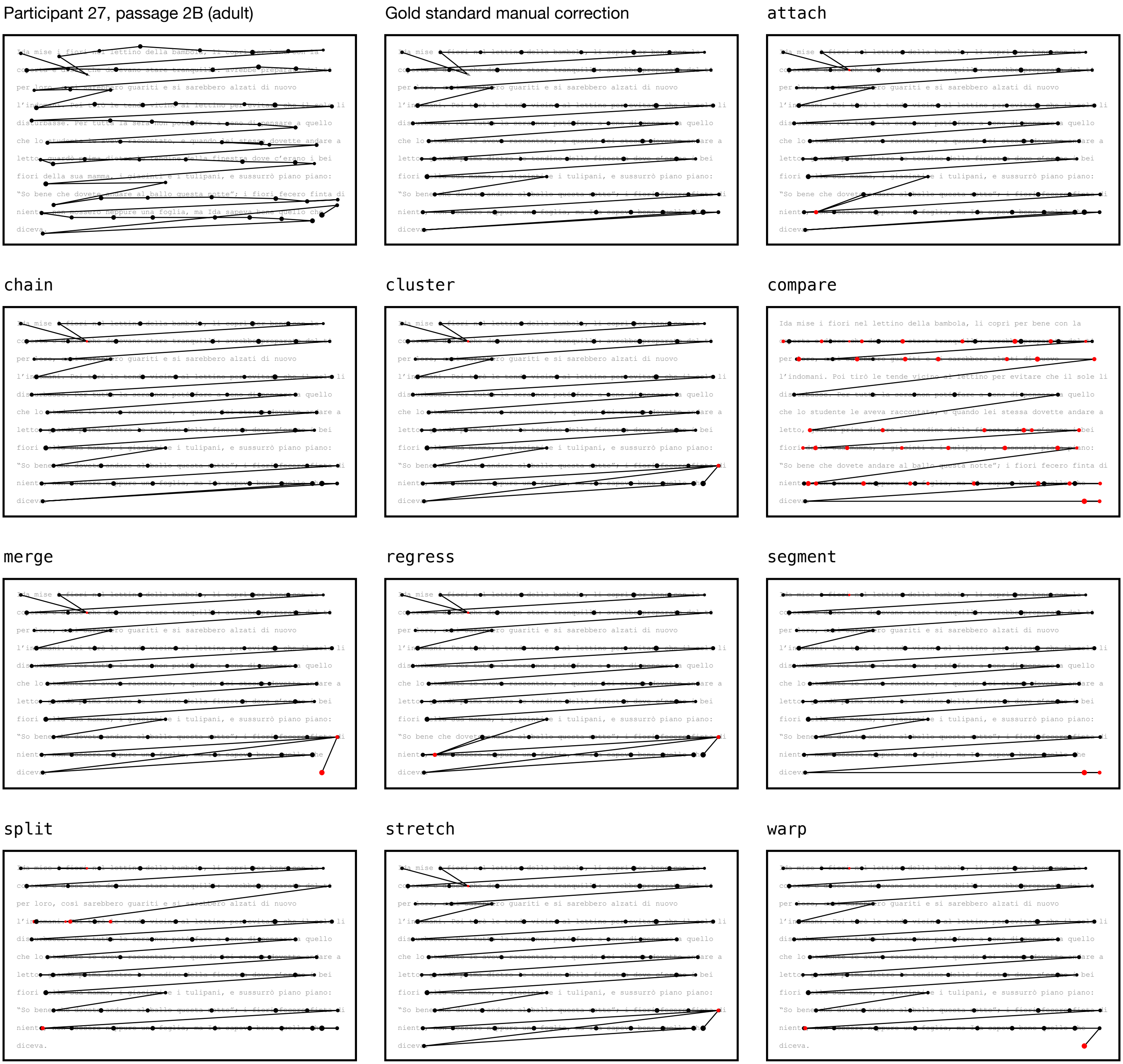
Participant 27, passage 2B (adult)
Gold standard manual correction
attach
chain
cluster
compare
merge
regress
segment
split
stretch
warp

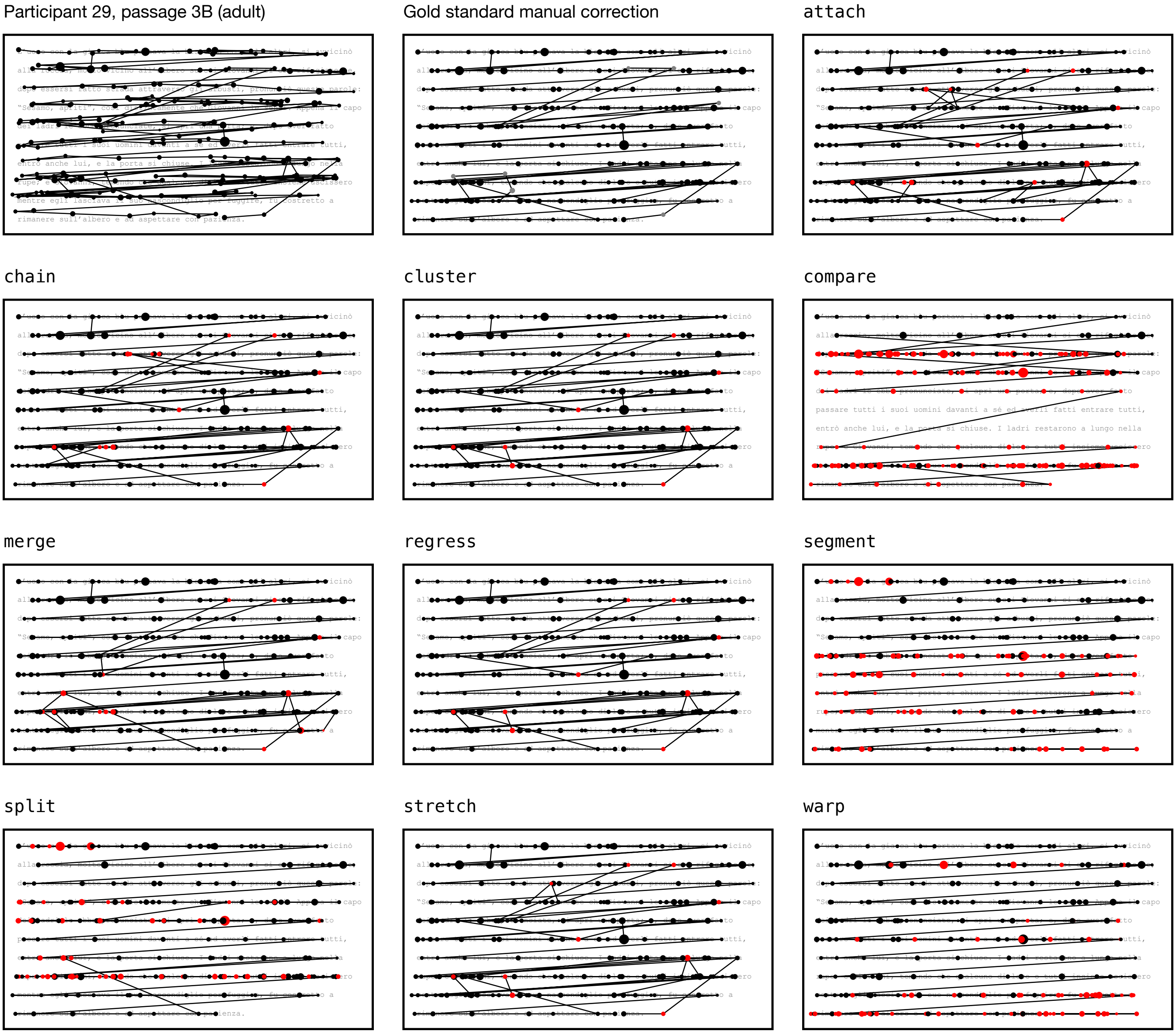
Participant 29, passage 3B (adult)
Gold standard manual correction
attach
chain
cluster
compare
merge
regress
segment
split
stretch
warp

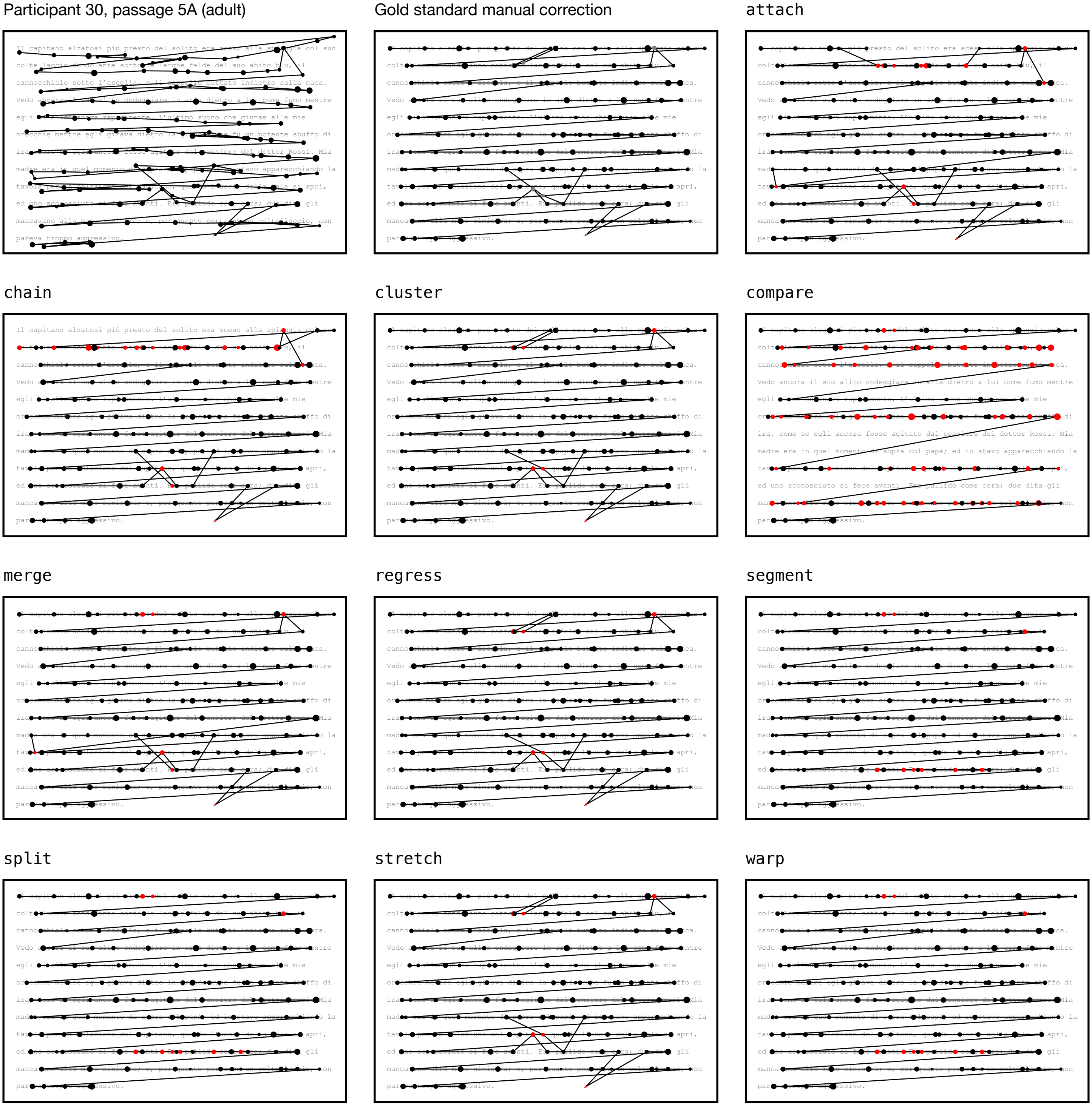
Participant 30, passage 5A (adult)
Gold standard manual correction
attach
chain
cluster
compare
merge
regress
segment
split
stretch
warp

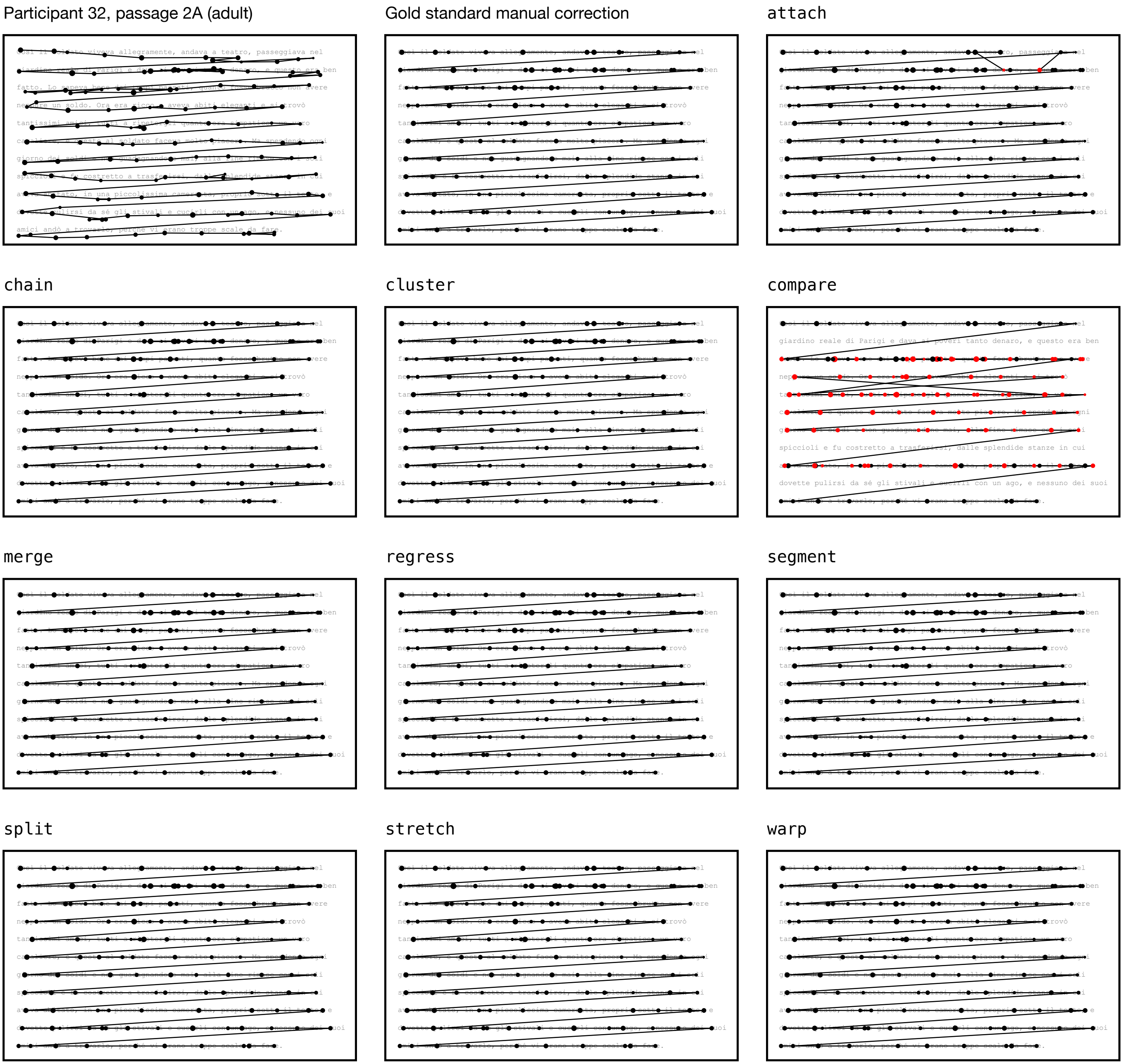

Participant 32, passage 2A (adult)
Gold standard manual correction
attach
chain
cluster
compare
merge
regress
segment
split
stretch
warp

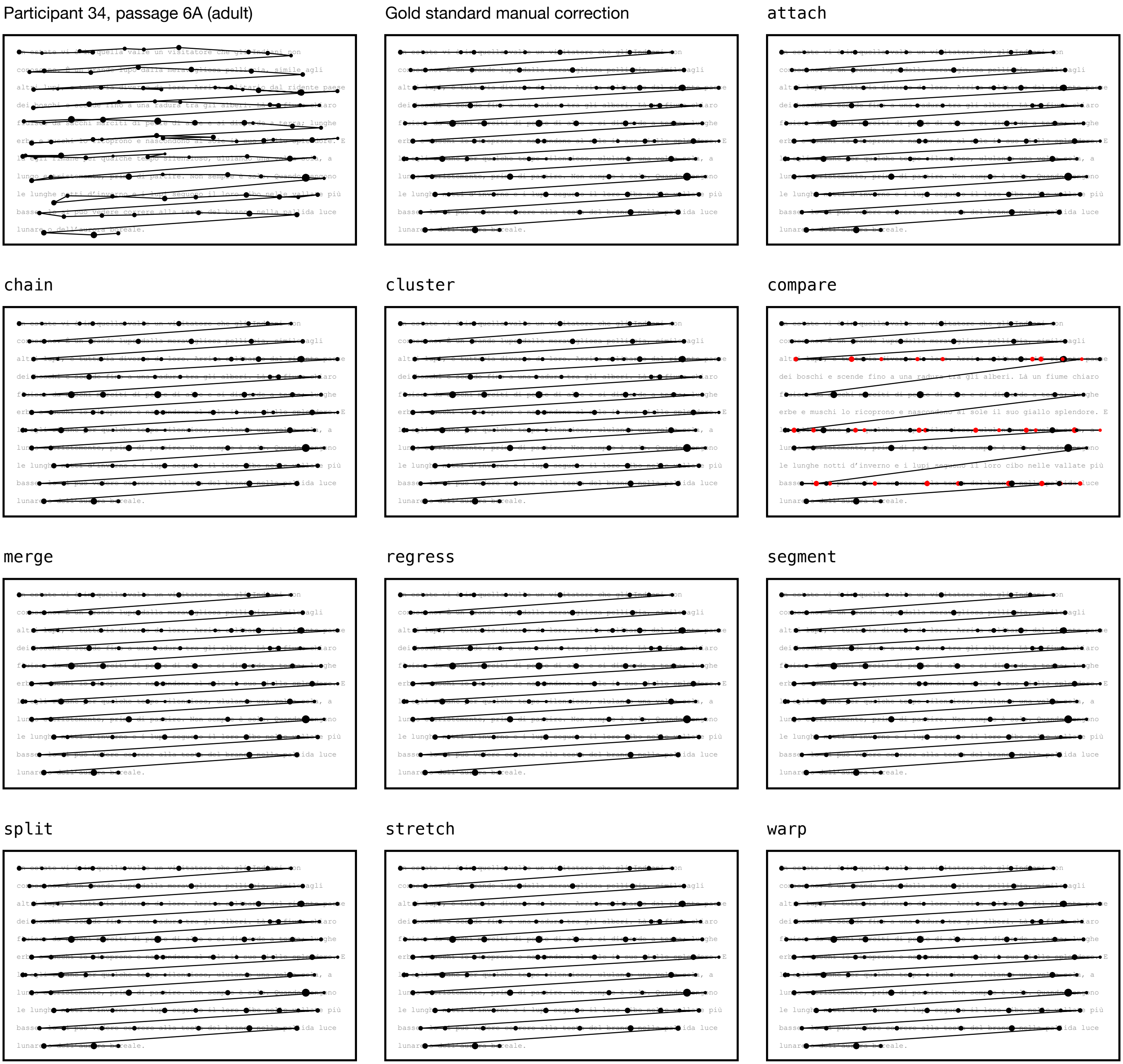
Participant 34, passage 6A (adult)
Gold standard manual correction
attach
chain
cluster
compare
merge
regress
segment
split
stretch
warp

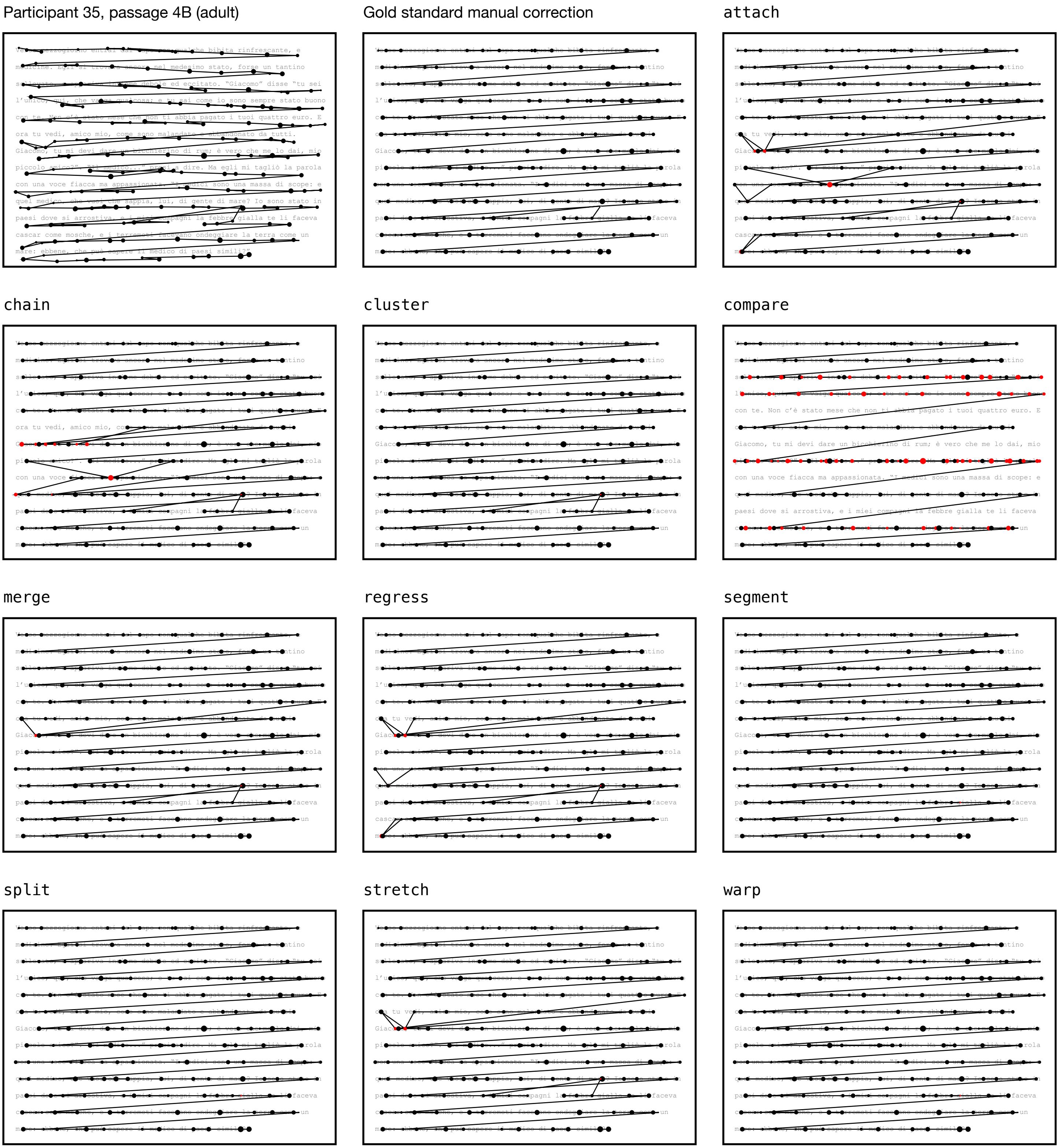
Participant 35, passage 4B (adult)
Gold standard manual correction
attach
chain
cluster
compare
merge
regress
segment
split
stretch
warp
ora tu vedi, amico mio, come sono malandato e abbandonato da tutti.
con te. Non c'è stato mese che non ti abbia pagato i tuoi quattro euro. E
Giacomo, tu mi devi dare un bicchierino di rum; è vero che me lo dai, mio
con una voce fiacca ma appassionata. "I medici sono una massa di scope: e
paesi dove si arrostiva, e i miei compagni la febbre gialla te li faceva

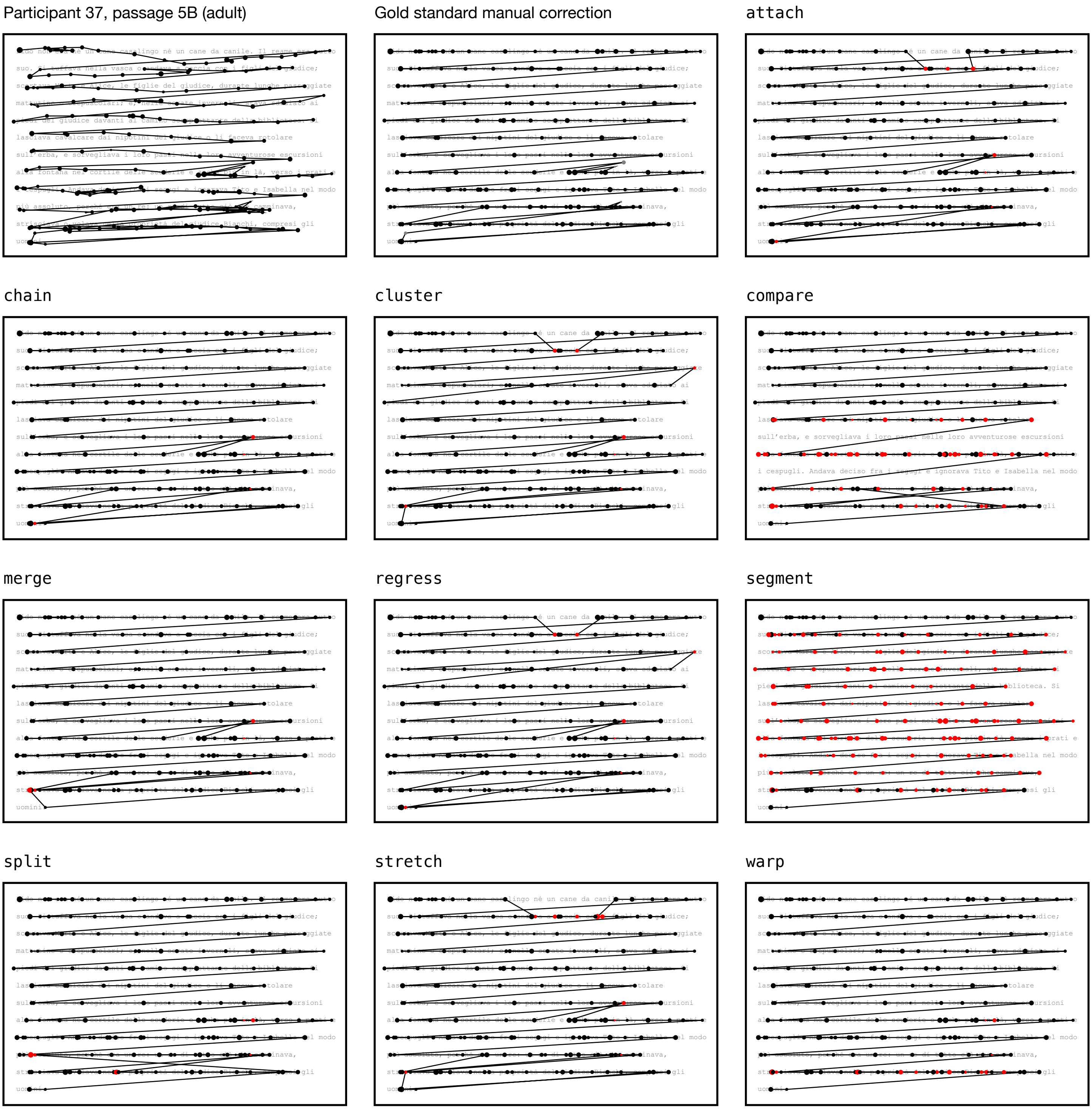
Participant 37, passage 5B (adult)
Gold standard manual correction
attach
chain
cluster
compare
merge
regress
segment
split
stretch
warp

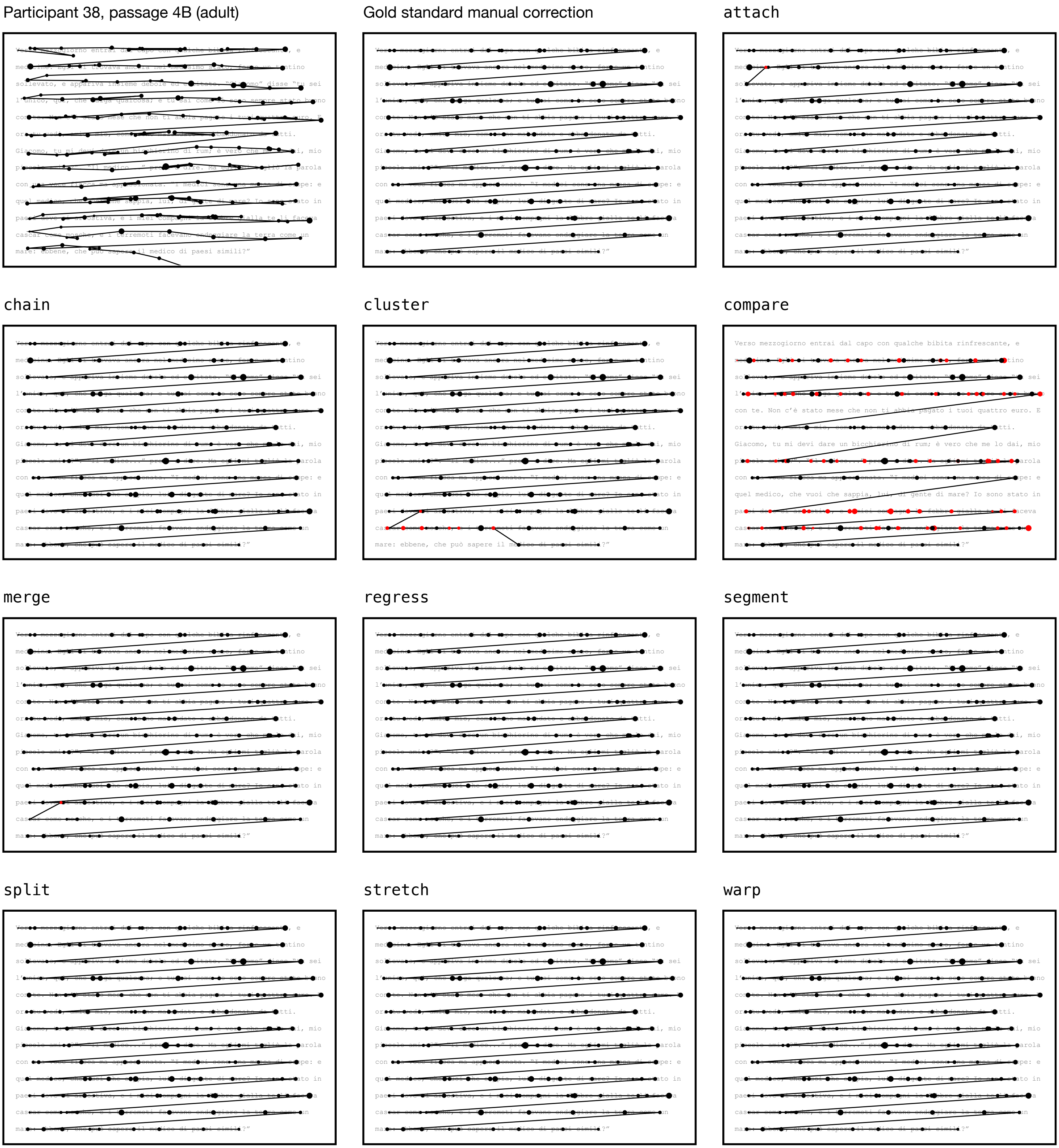
Participant 38, passage 4B (adult)
Gold standard manual correction
attach
chain
cluster
compare
merge
regress
segment
split
stretch
warp
Verso mezzogiorno entrai dal capo con qualche bibita rinfrescante, e
con te. Non c’è stato mese che non ti abbia pagato i tuoi quattro euro. E
Giacomo, tu mi devi dare un bicchierino di rum; è vero che me lo dai, mio
quel medico, che vuoi che sappia, lui, di gente di mare? Io sono stato in
mare: ebbene, che può sapere il medico di paesi simili?”

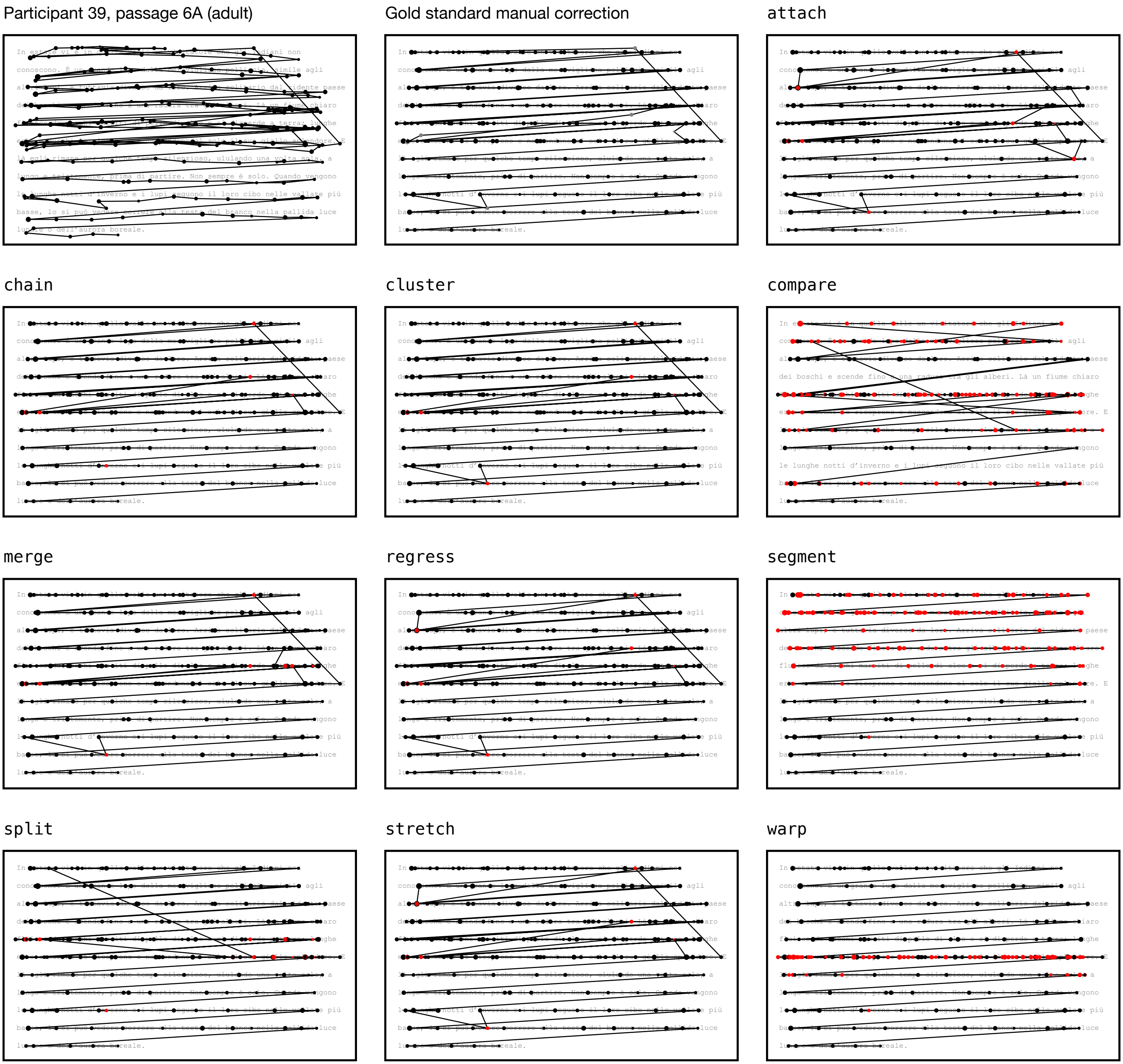
Participant 39, passage 6A (adult)
Gold standard manual correction
attach
chain
cluster
compare
merge
regress
segment
split
stretch
warp

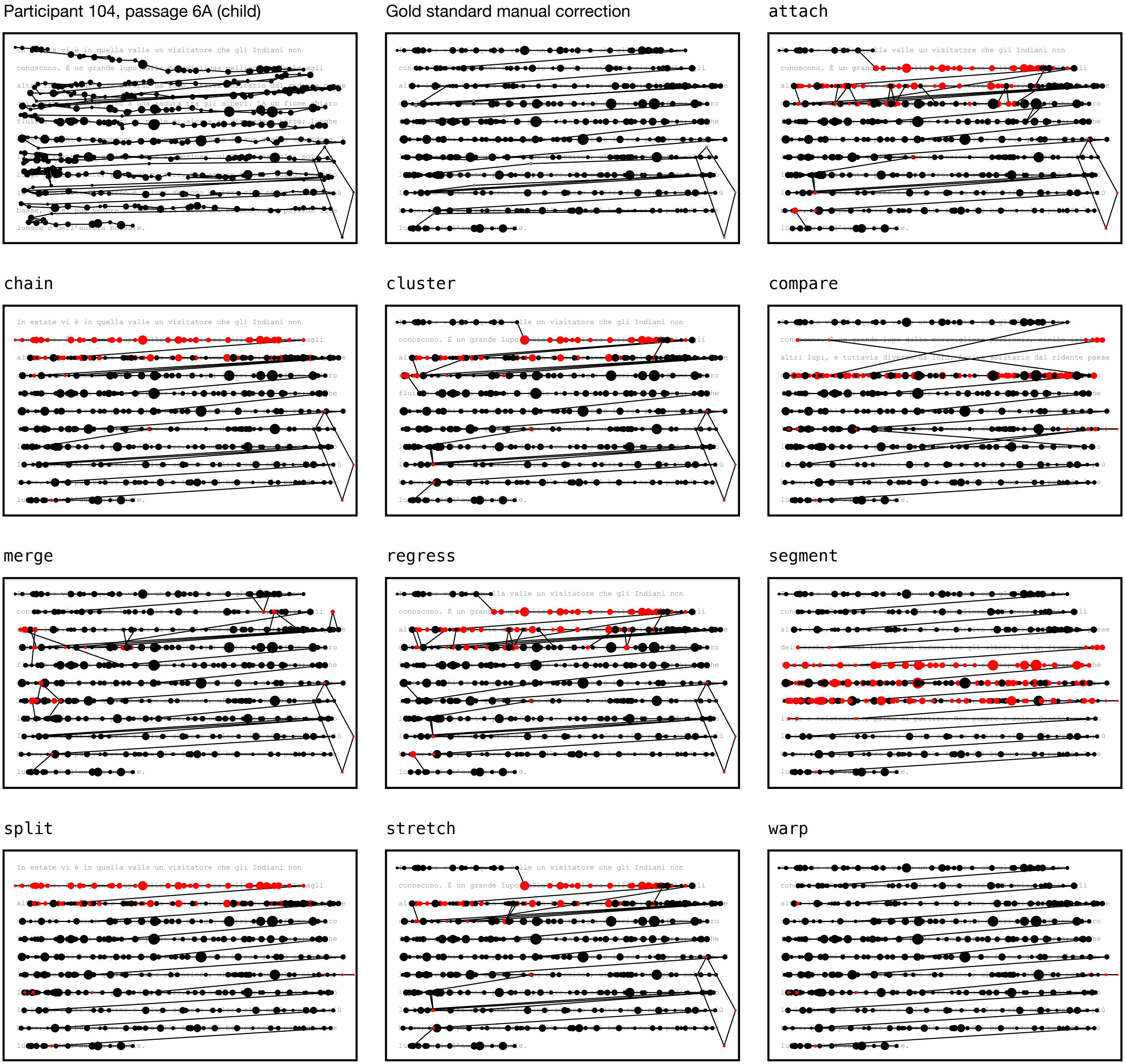
Participant 104, passage 6A (child)
Gold standard manual correction
attach
chain
cluster
compare
merge
regress
segment
split
stretch
warp

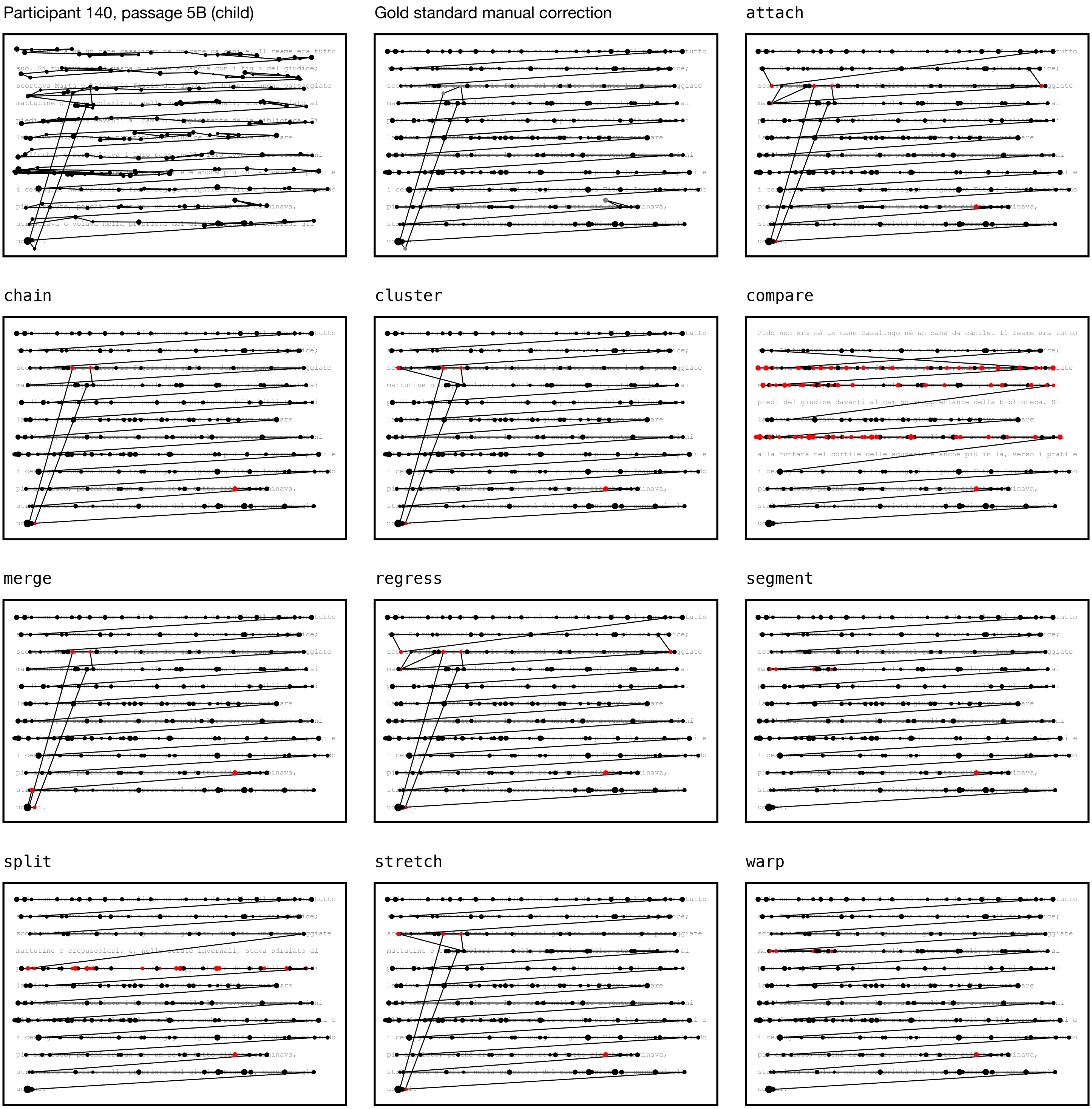
Participant 140, passage 5B (child)
Gold standard manual correction
attach
chain
cluster
compare
merge
regress
segment
split
stretch
warp

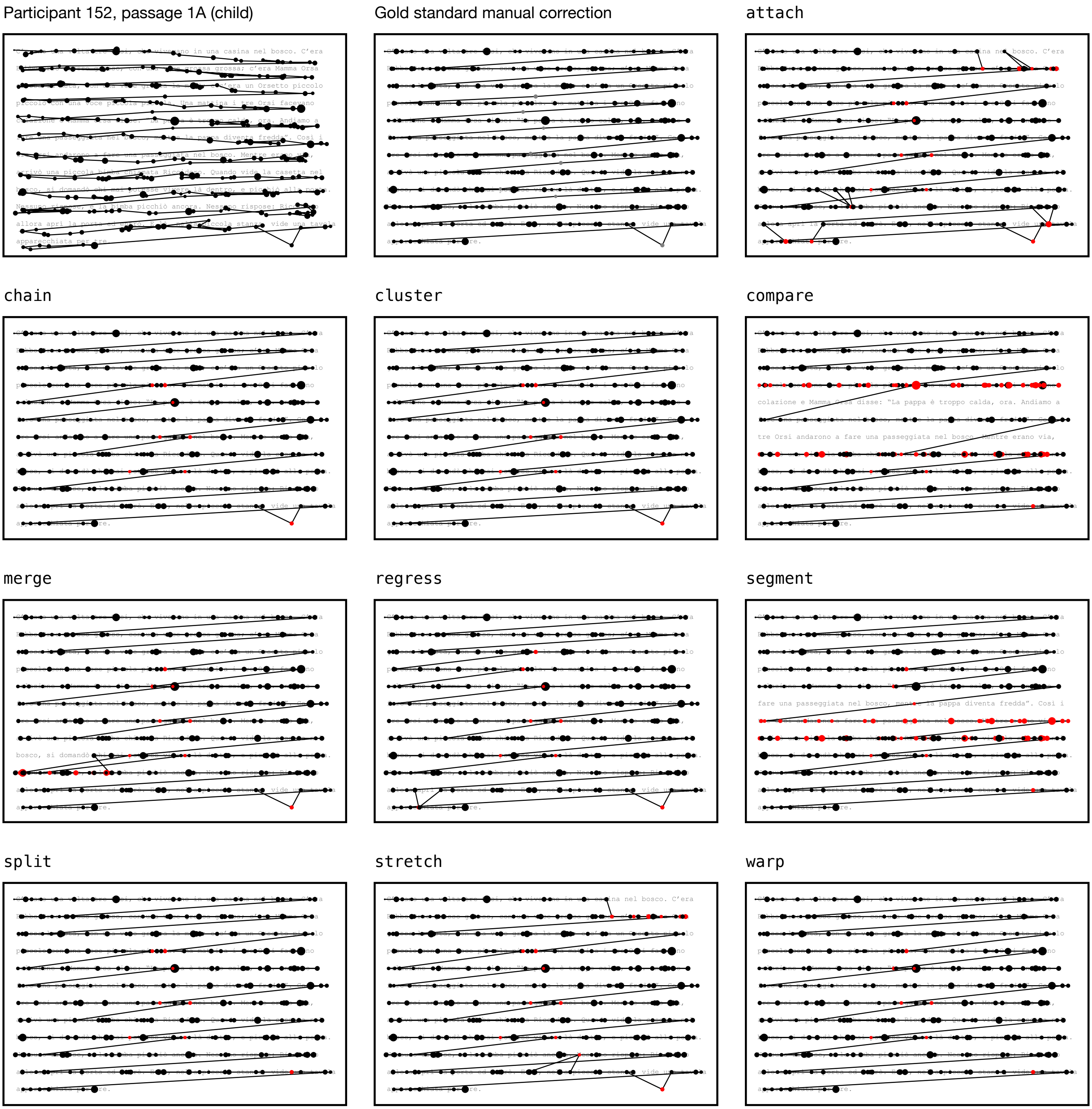
Participant 152, passage 1A (child)
Gold standard manual correction
attach
chain
cluster
compare
merge
regress
segment
split
stretch
warp

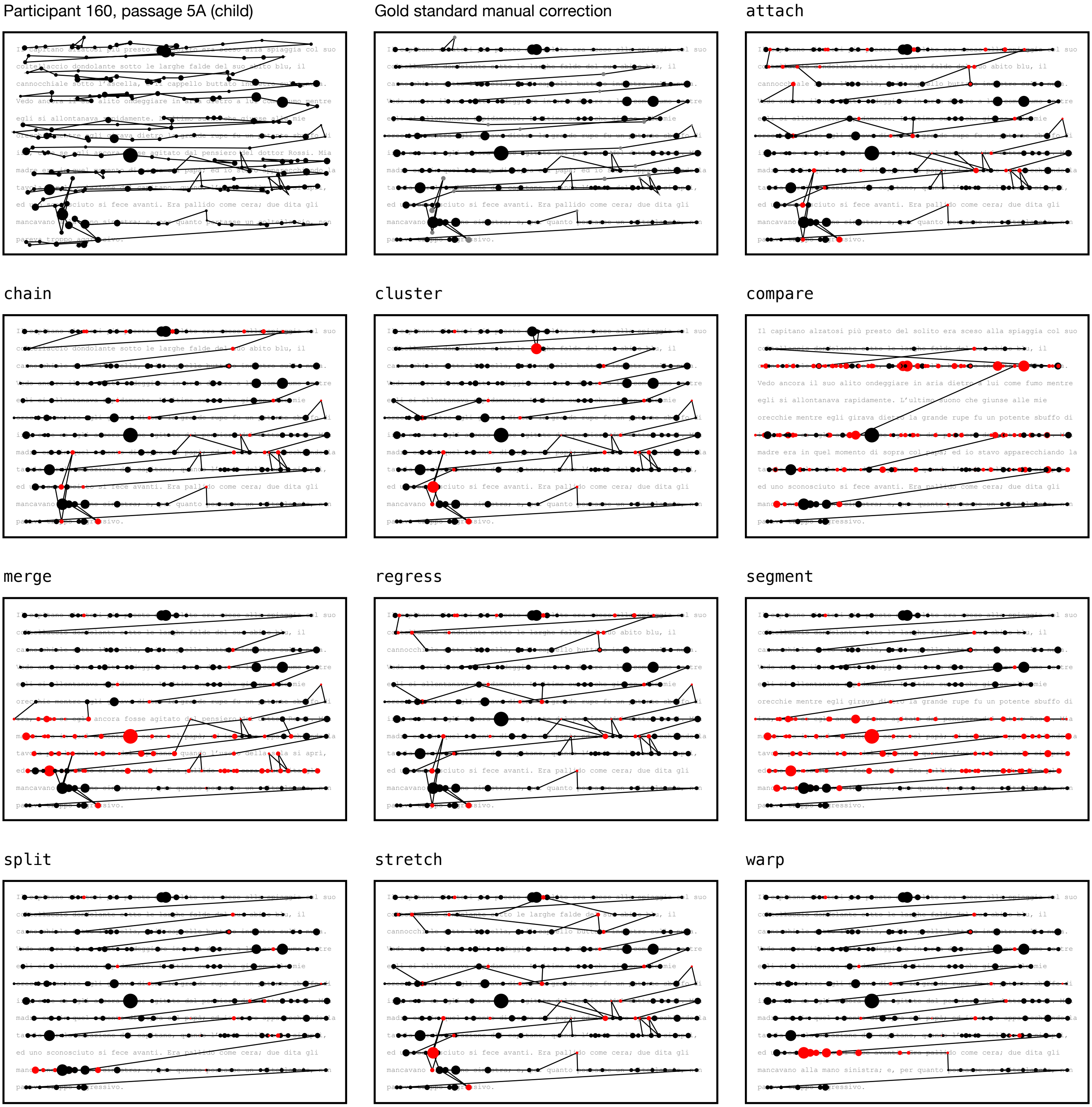
Participant 160, passage 5A (child)
Gold standard manual correction
attach
chain
cluster
compare
merge
regress
segment
split
stretch
warp

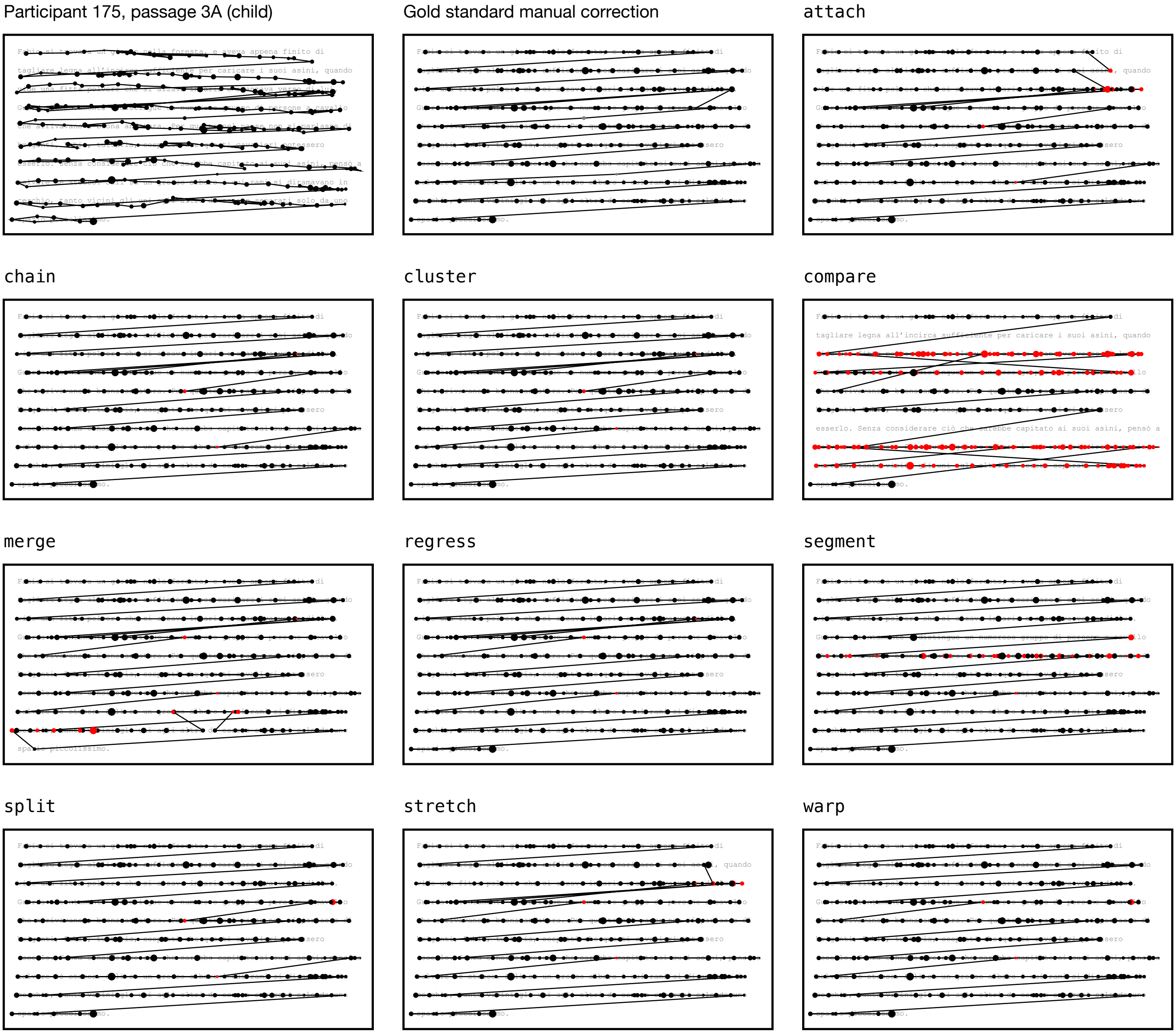
Participant 175, passage 3A (child)
Gold standard manual correction
attach
chain
cluster
compare
merge
regress
segment
split
stretch
warp

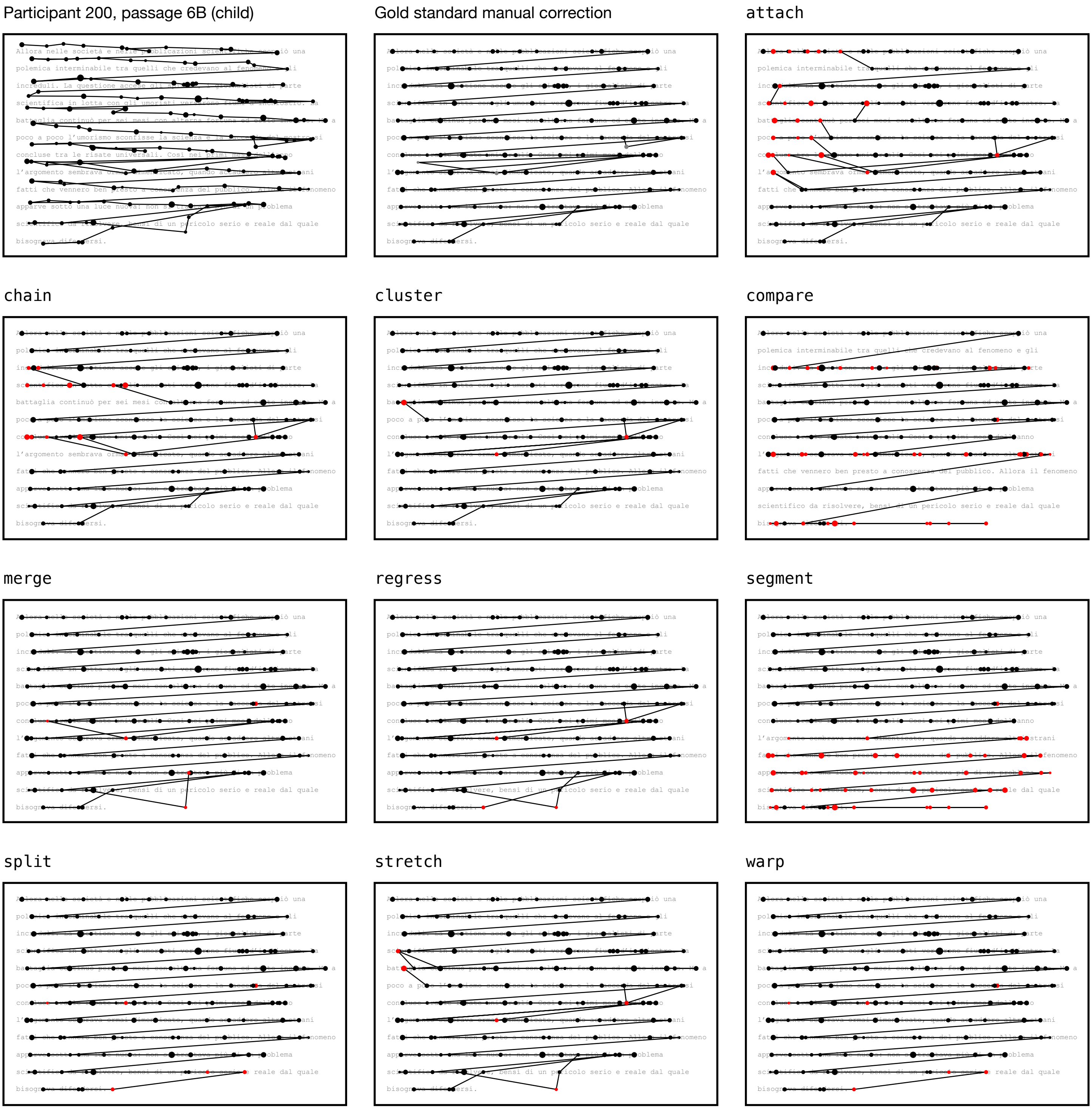
Participant 200, passage 6B (child)
Gold standard manual correction
attach
chain
cluster
compare
merge
regress
segment
split
stretch
warp

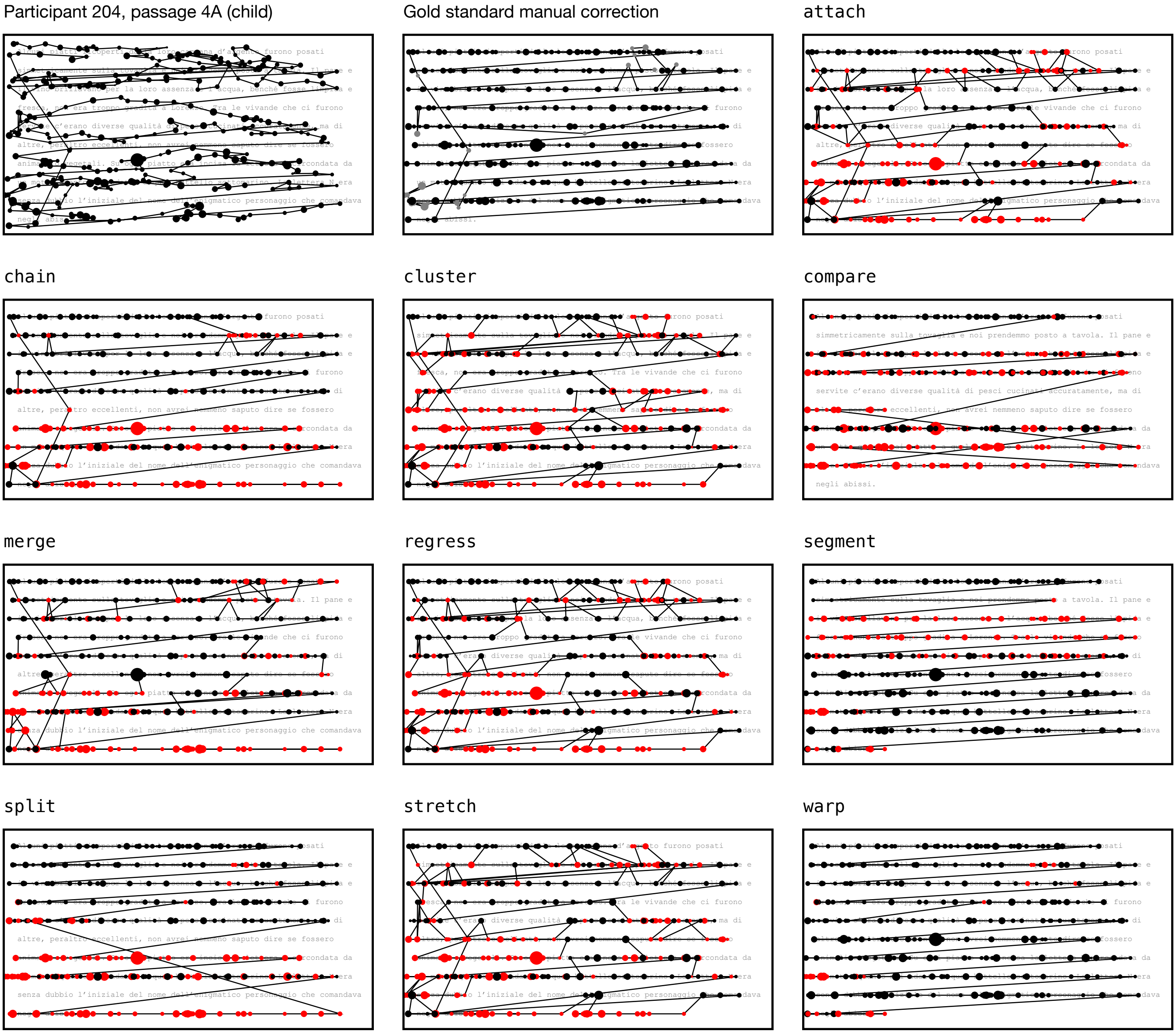
Participant 204, passage 4A (child)
Gold standard manual correction
attach
chain
cluster
compare
merge
regress
segment
split
stretch
warp

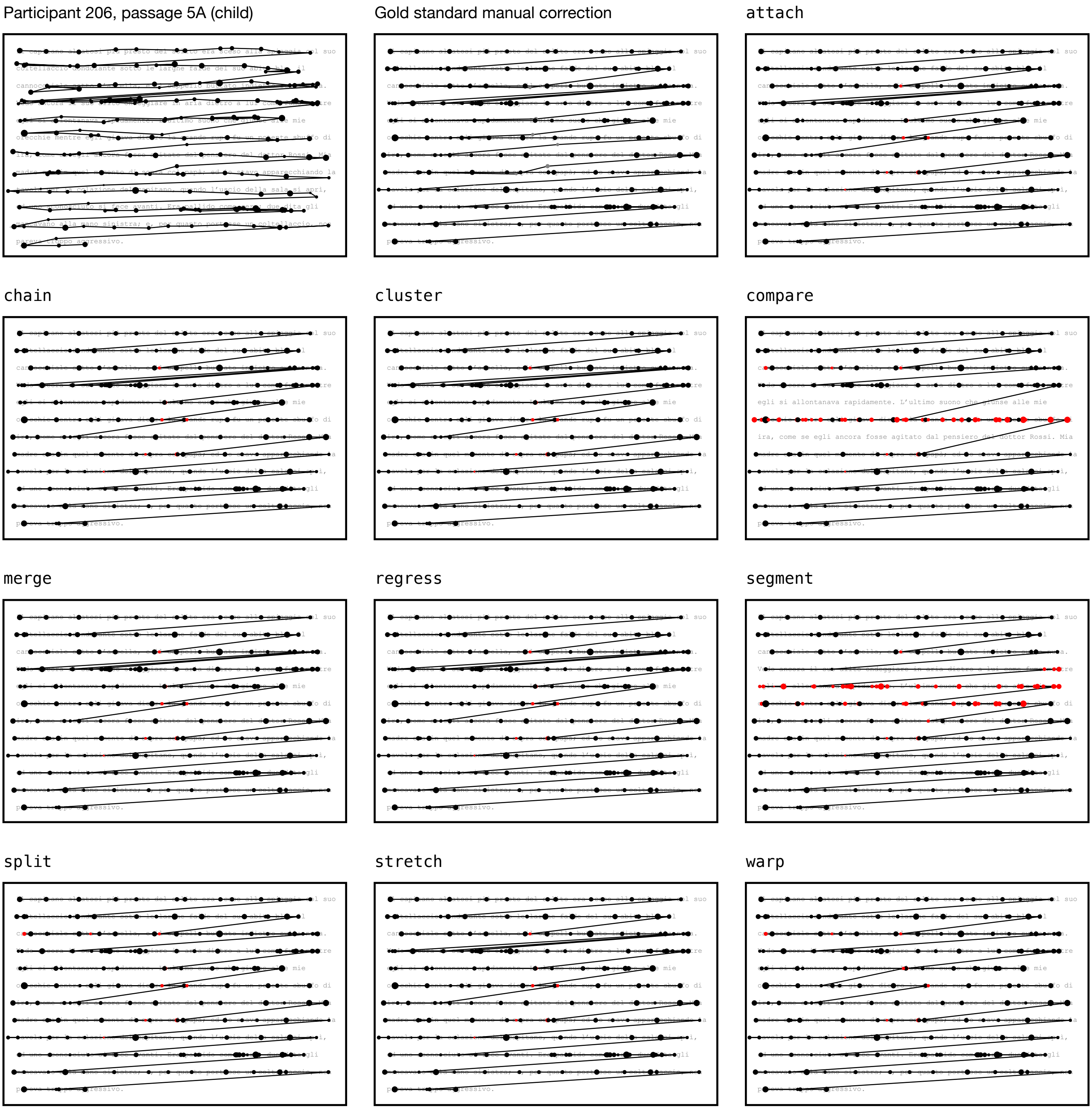
Participant 206, passage 5A (child)
Gold standard manual correction
attach
chain
cluster
compare
merge
regress
segment
split
stretch
warp

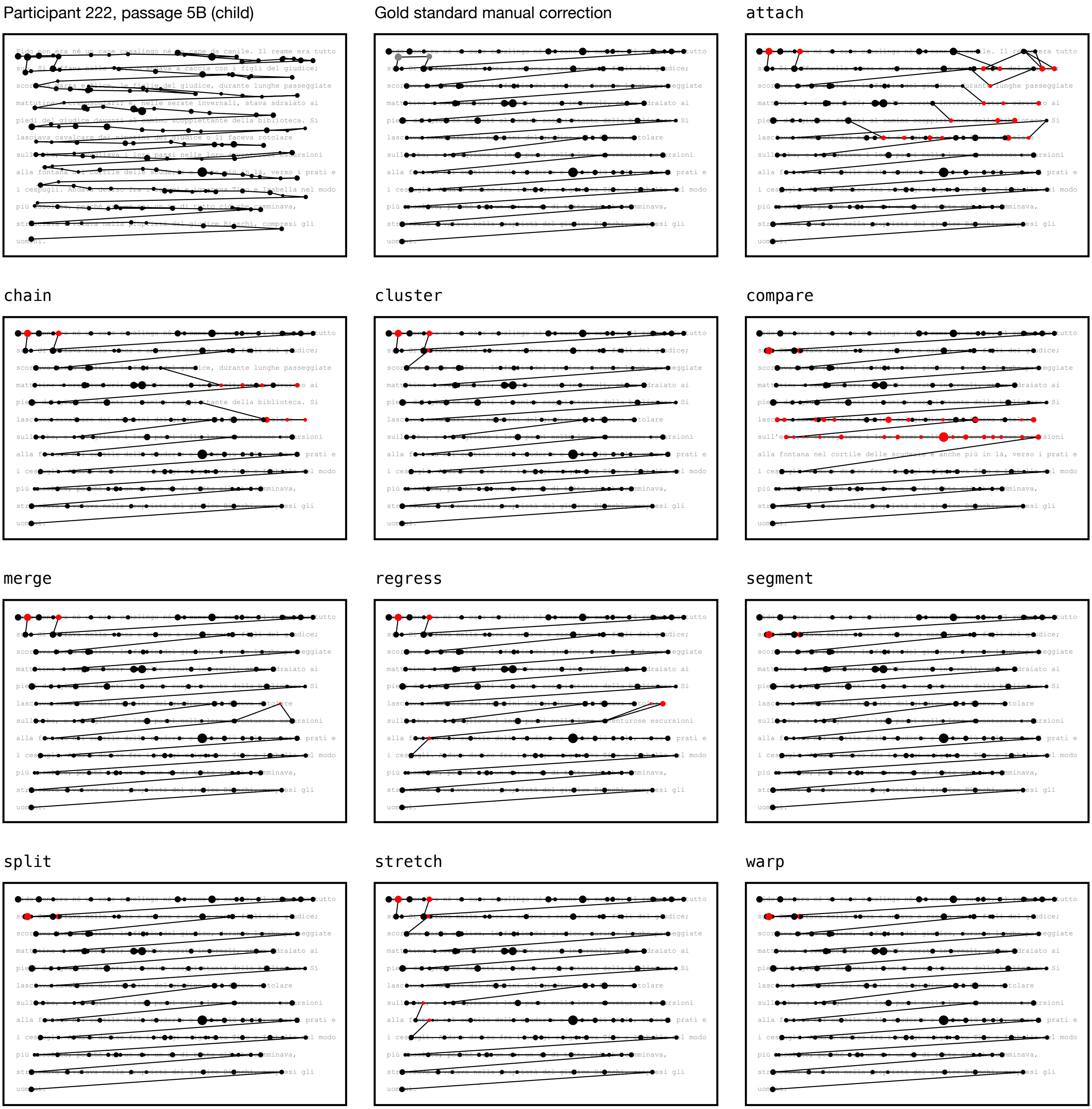
Participant 222, passage 5B (child)
Gold standard manual correction
attach
chain
cluster
compare
merge
regress
segment
split
stretch
warp

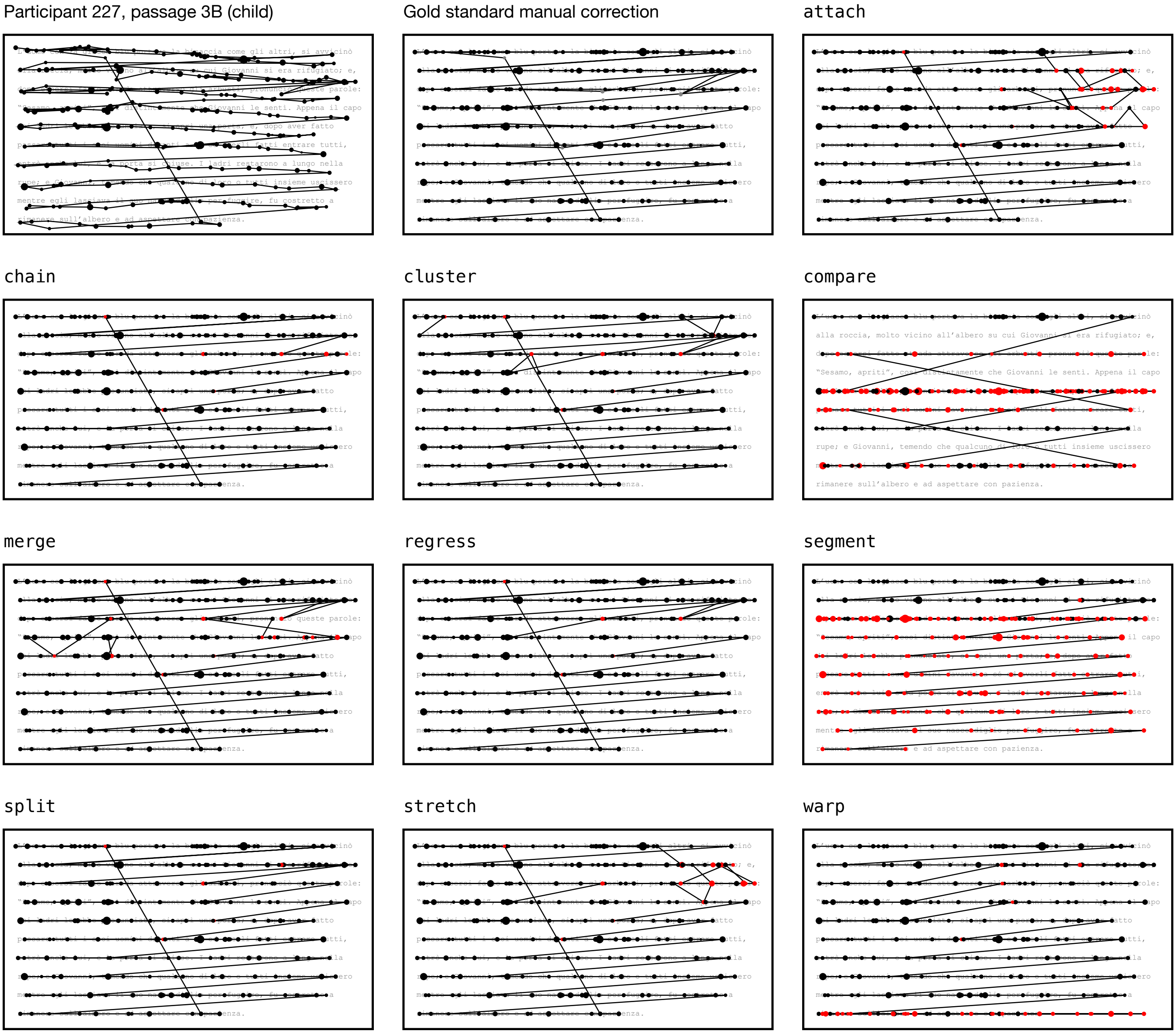
Participant 227, passage 3B (child)
Gold standard manual correction
attach
chain
cluster
compare
merge
regress
segment
split
stretch
warp

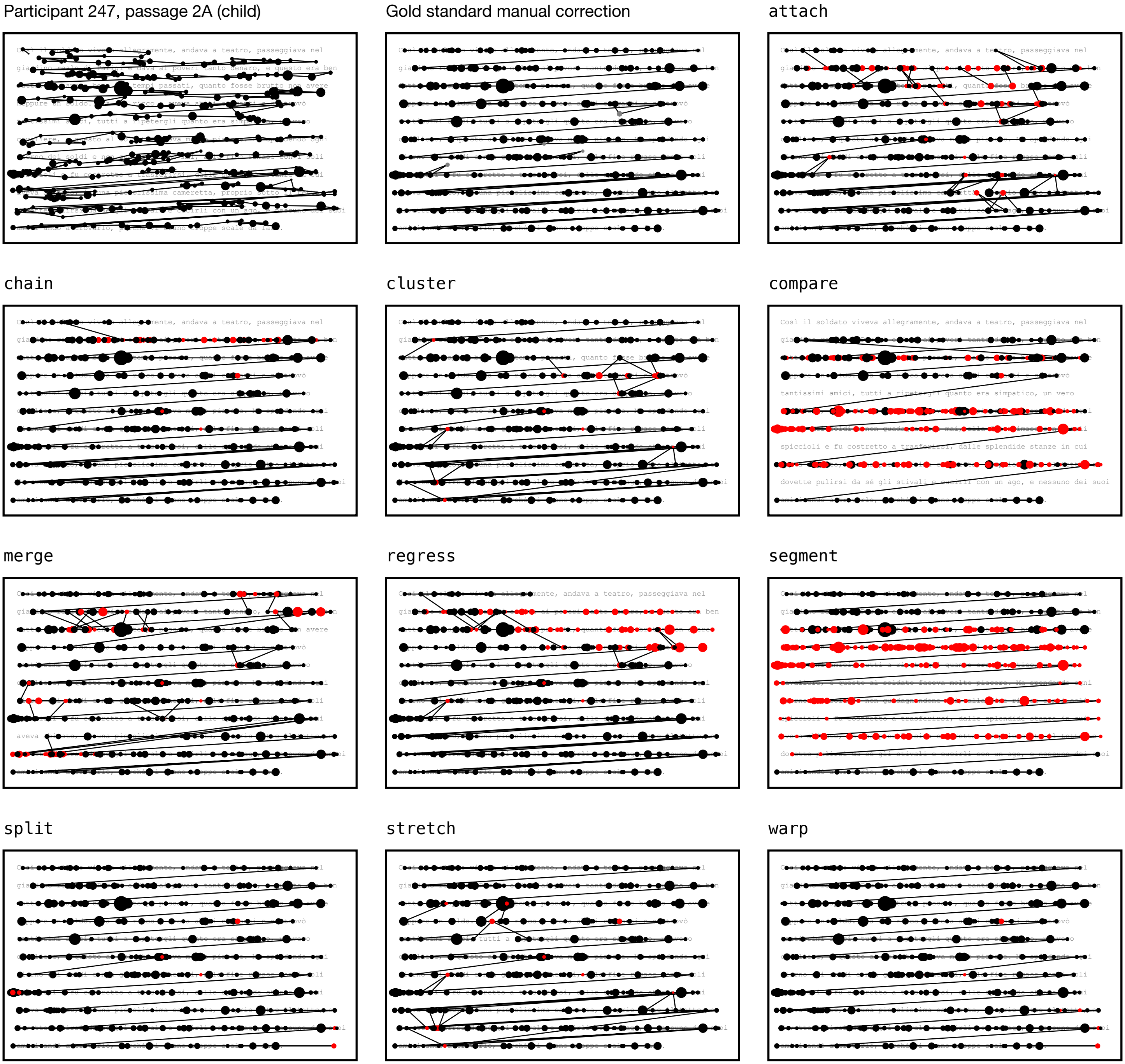
Participant 247, passage 2A (child)
Gold standard manual correction
attach
chain
cluster
compare
merge
regress
segment
split
stretch
warp

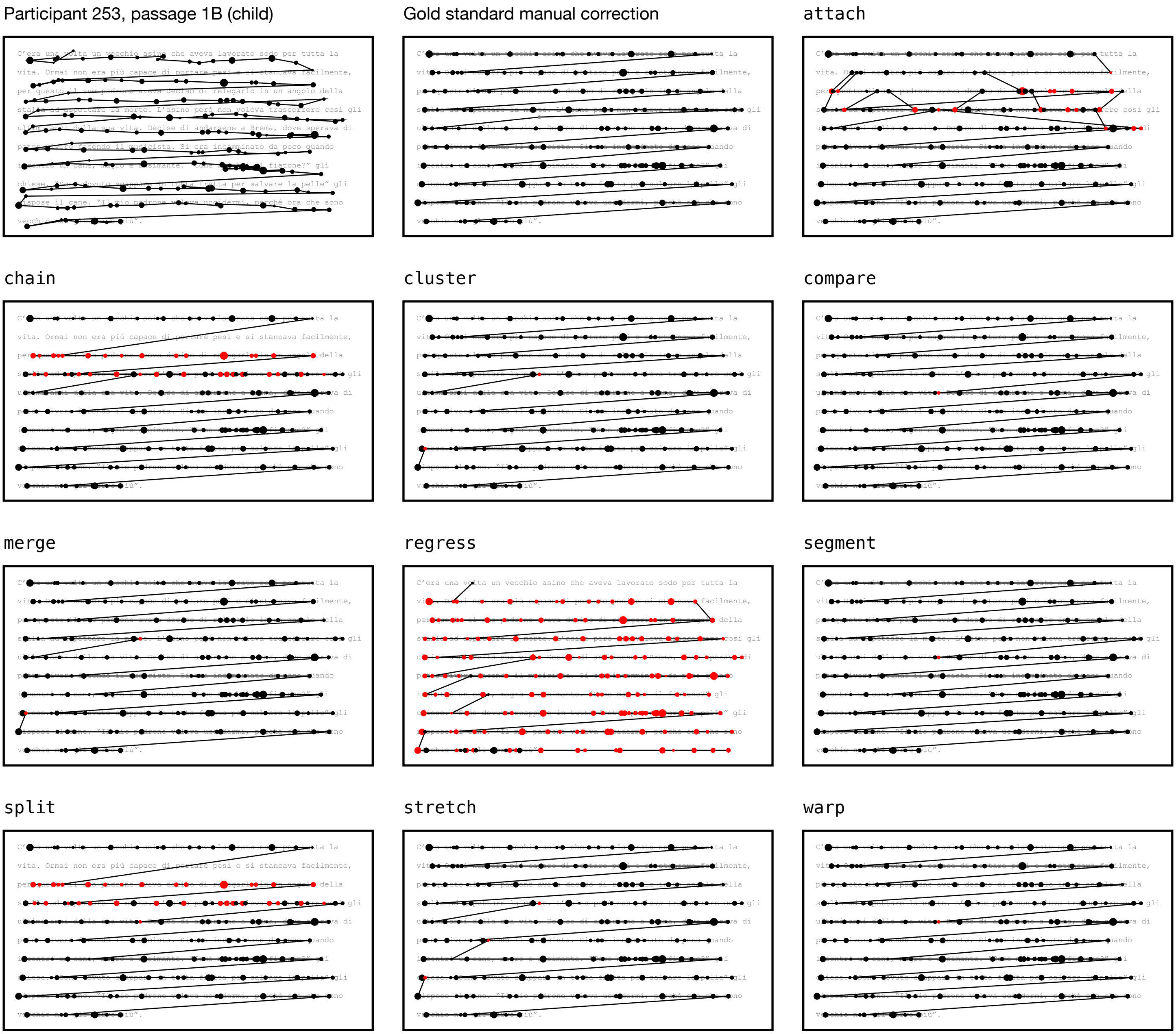
Participant 253, passage 1B (child)
Gold standard manual correction
attach
chain
cluster
compare
merge
regress
segment
split
stretch
warp

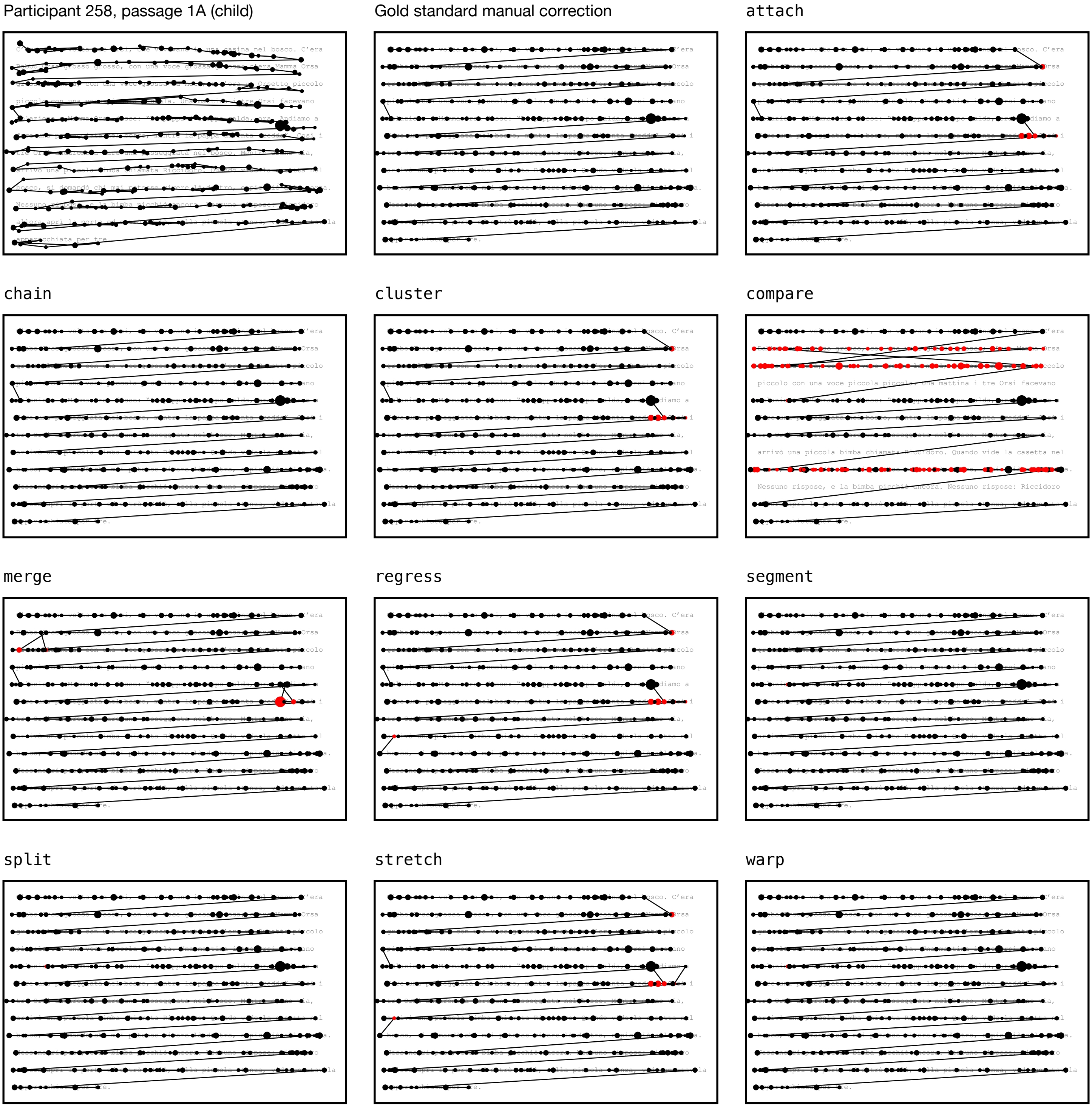

Participant 258, passage 1A (child)
Gold standard manual correction
attach
chain
cluster
compare
merge
regress
segment
split
stretch
warp

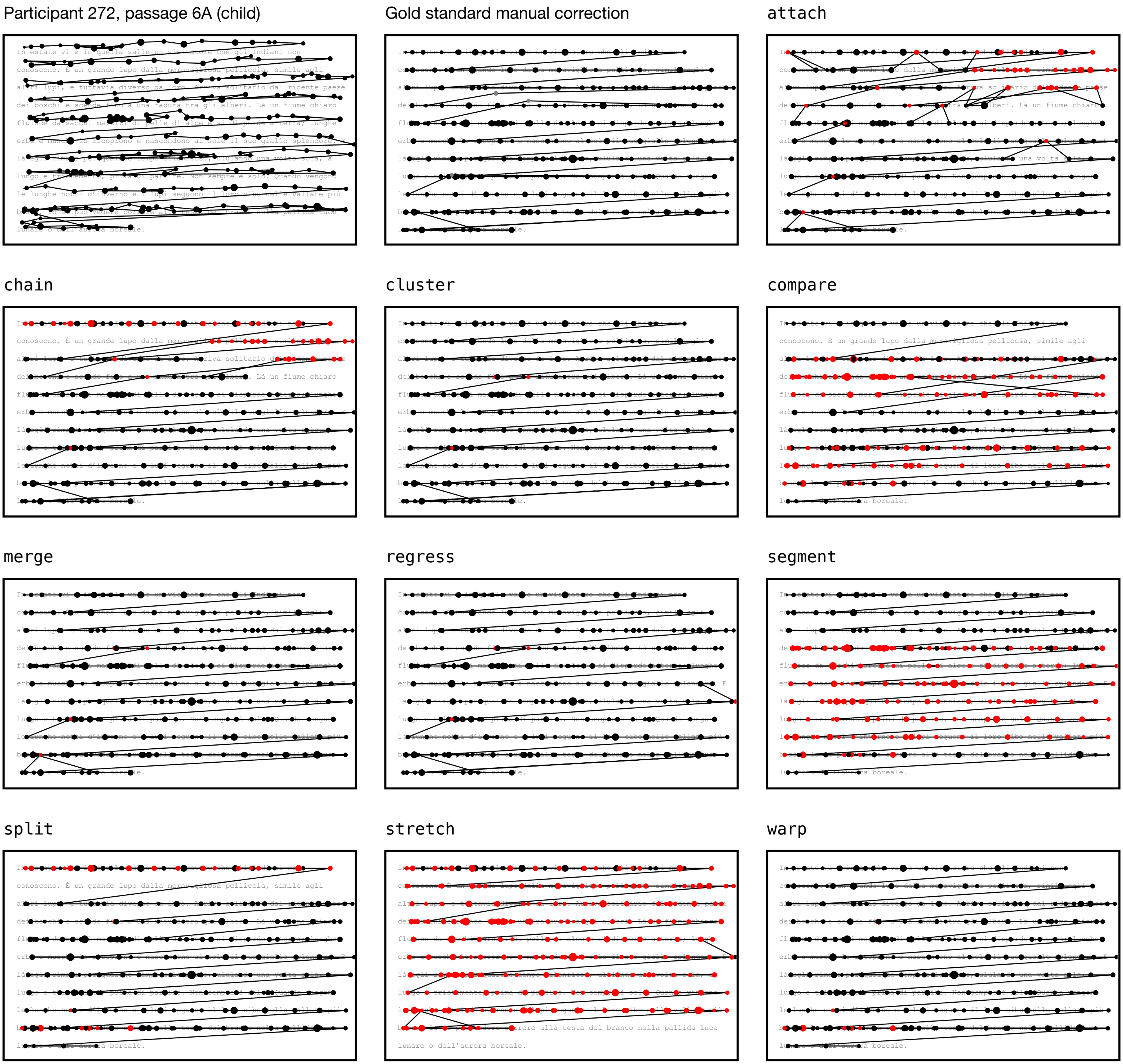
Participant 272, passage 6A (child)
Gold standard manual correction
attach
chain
cluster
compare
merge
regress
segment
split
stretch
warp

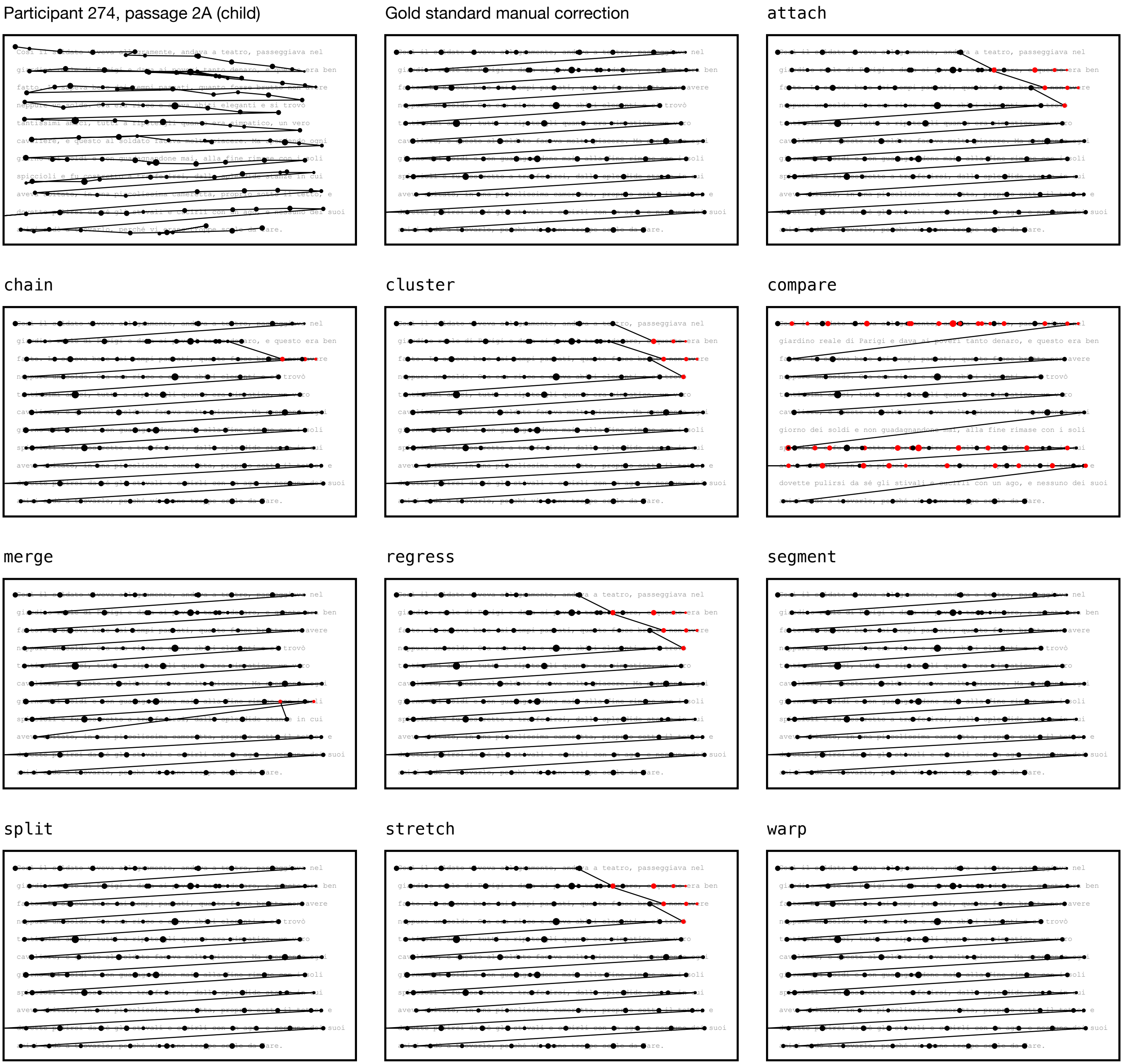
Participant 274, passage 2A (child)
Gold standard manual correction
attach
chain
cluster
compare
merge
regress
segment
split
stretch
warp

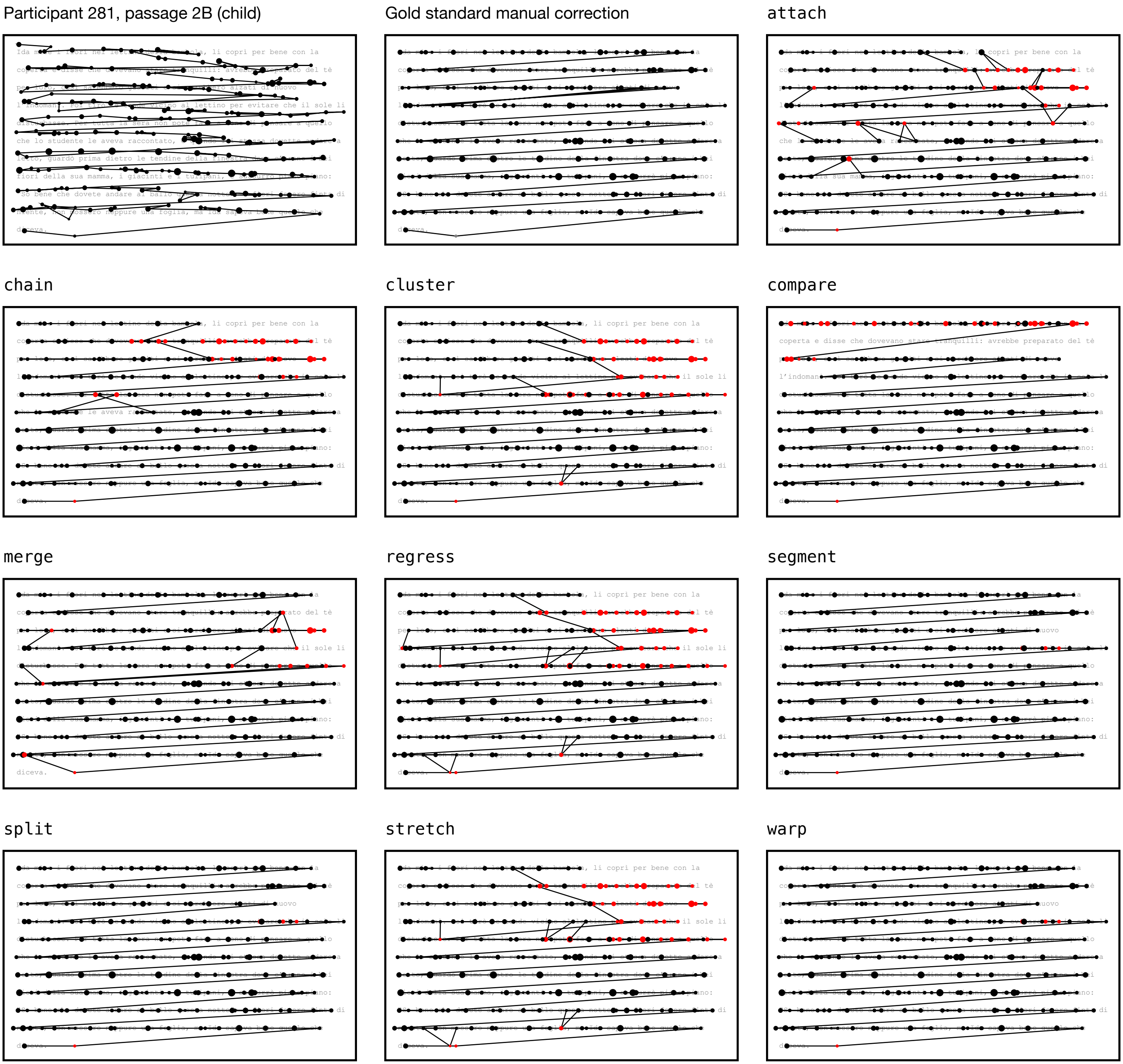

Participant 281, passage 2B (child)
Gold standard manual correction
attach
chain
cluster
compare
merge
regress
segment
split
stretch
warp

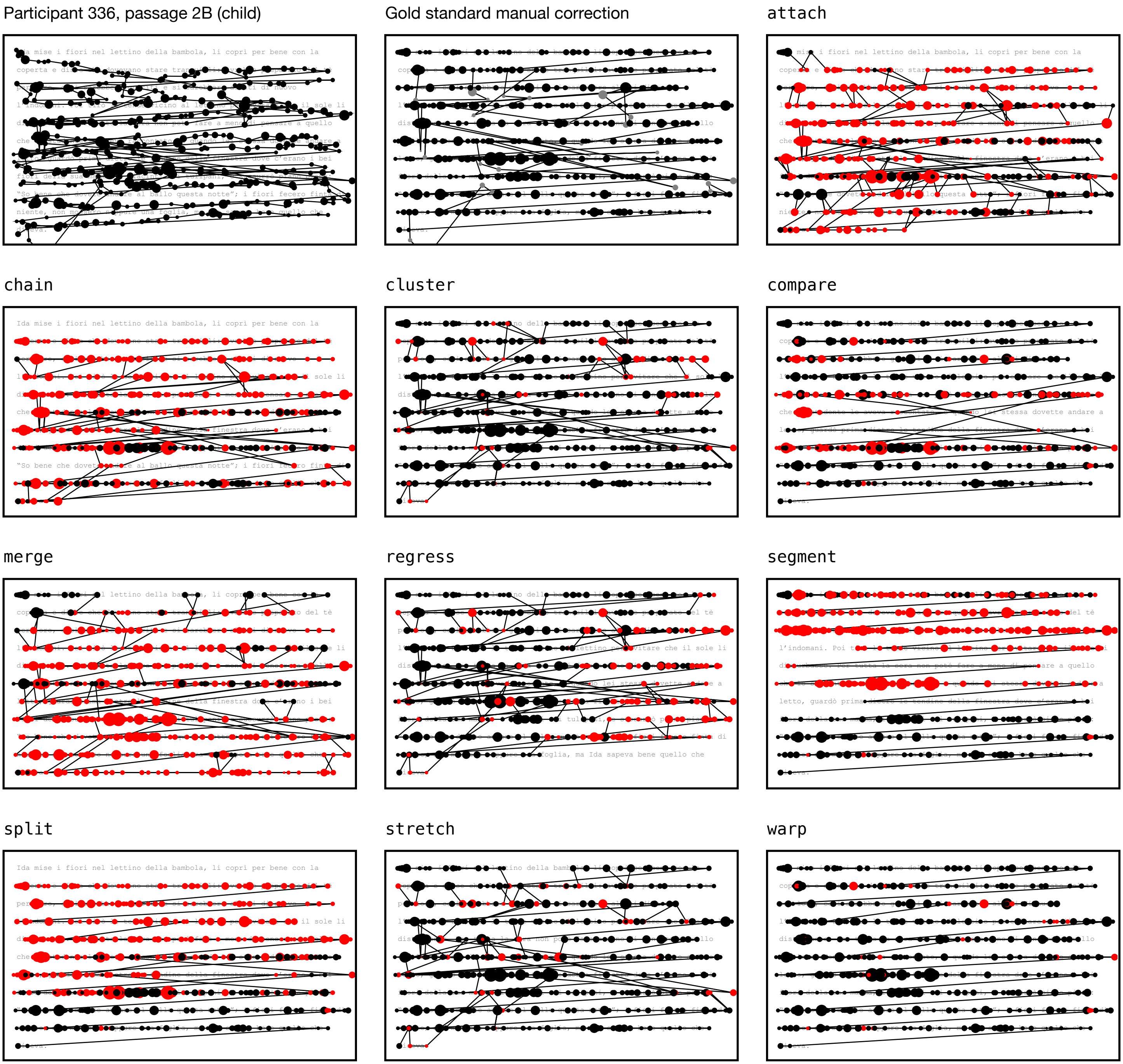
Participant 336, passage 2B (child)
Gold standard manual correction
attach
chain
cluster
compare
merge
regress
segment
split
stretch
warp

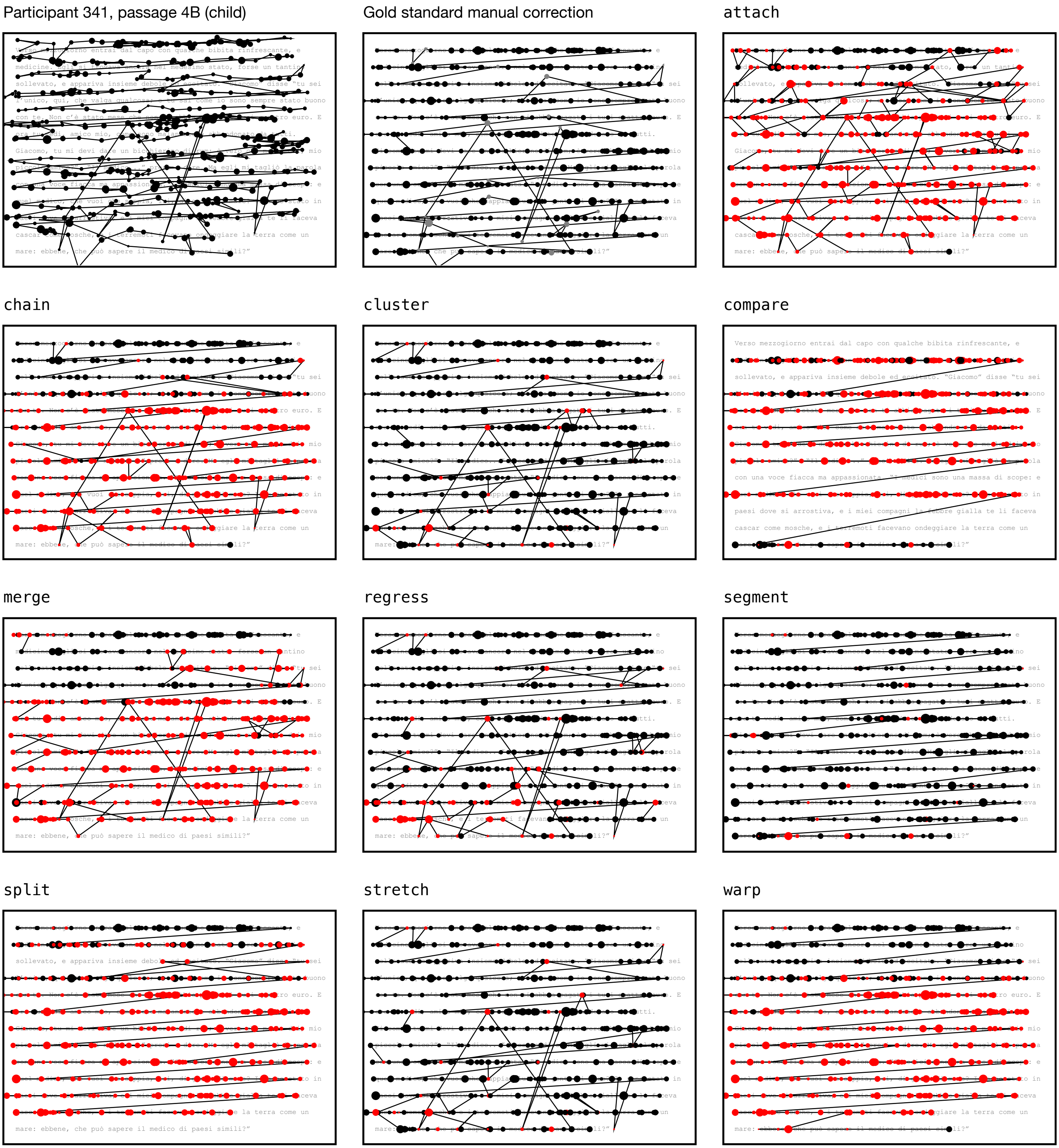
Participant 341, passage 4B (child)
Gold standard manual correction
attach
chain
cluster
compare
merge
regress
segment
split
stretch
warp

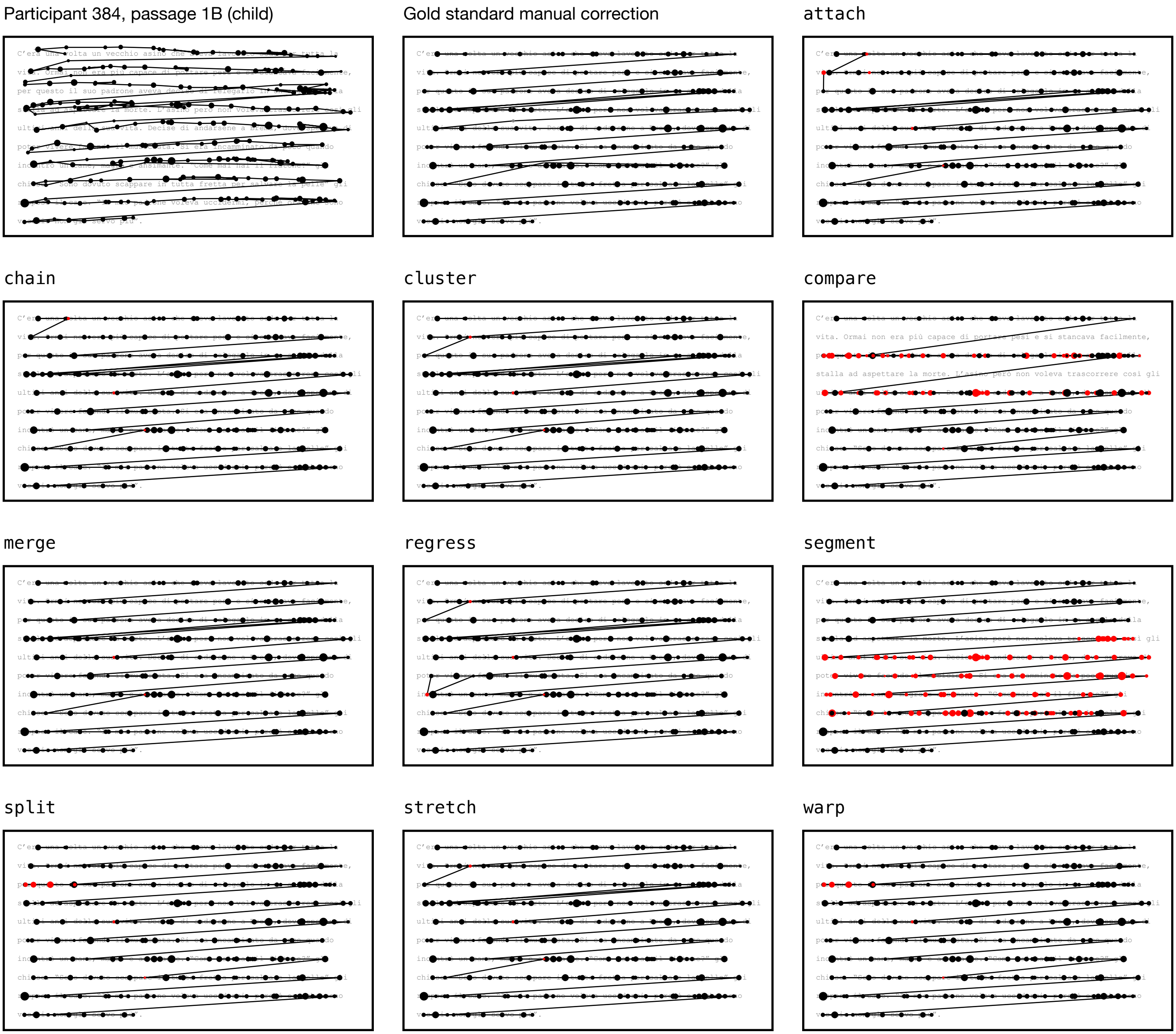
Participant 384, passage 1B (child)
Gold standard manual correction
attach
chain
cluster
compare
merge
regress
segment
split
stretch
warp

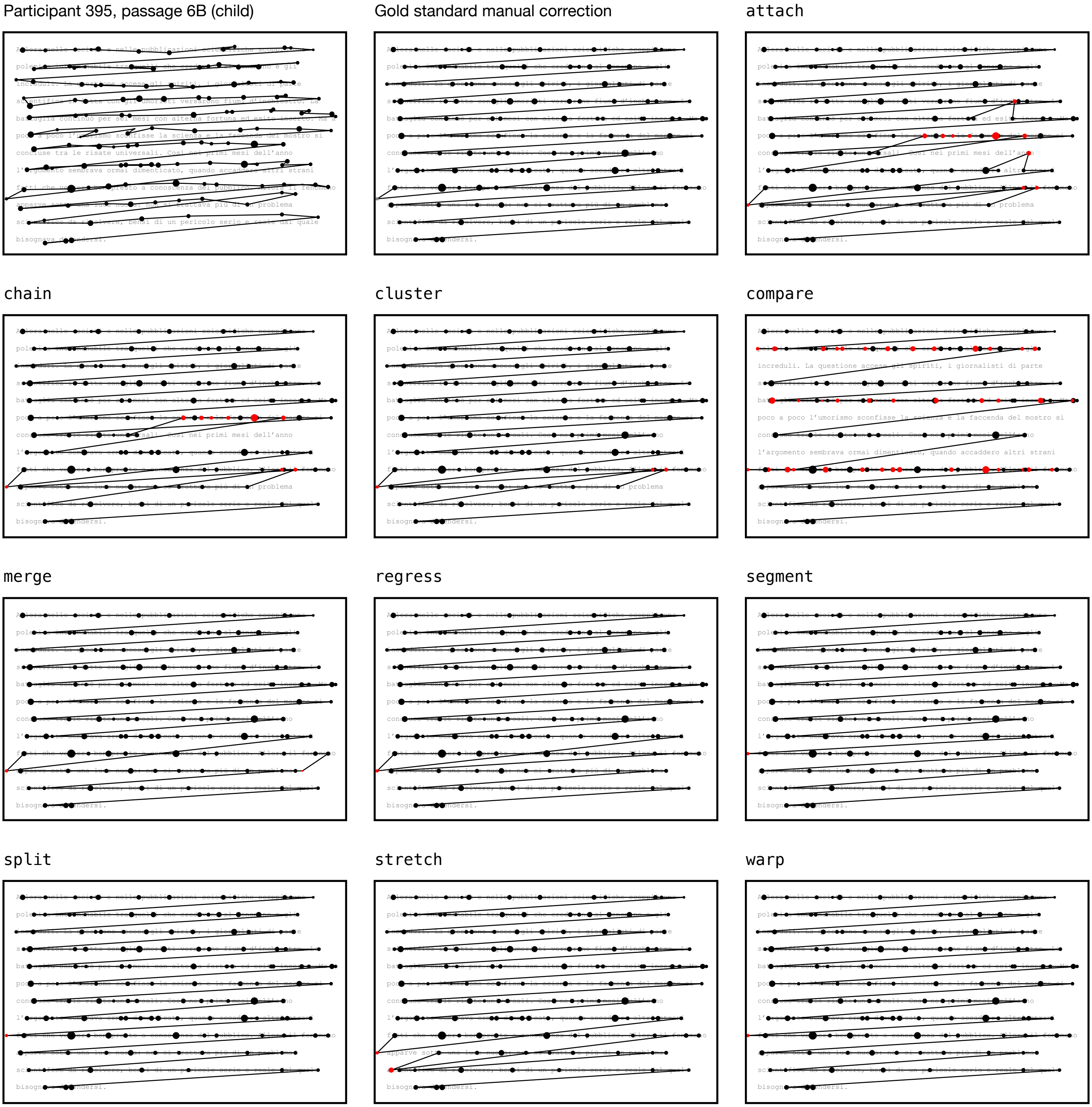

Participant 395, passage 6B (child)
Gold standard manual correction
attach
chain
cluster
compare
merge
regress
segment
split
stretch
warp

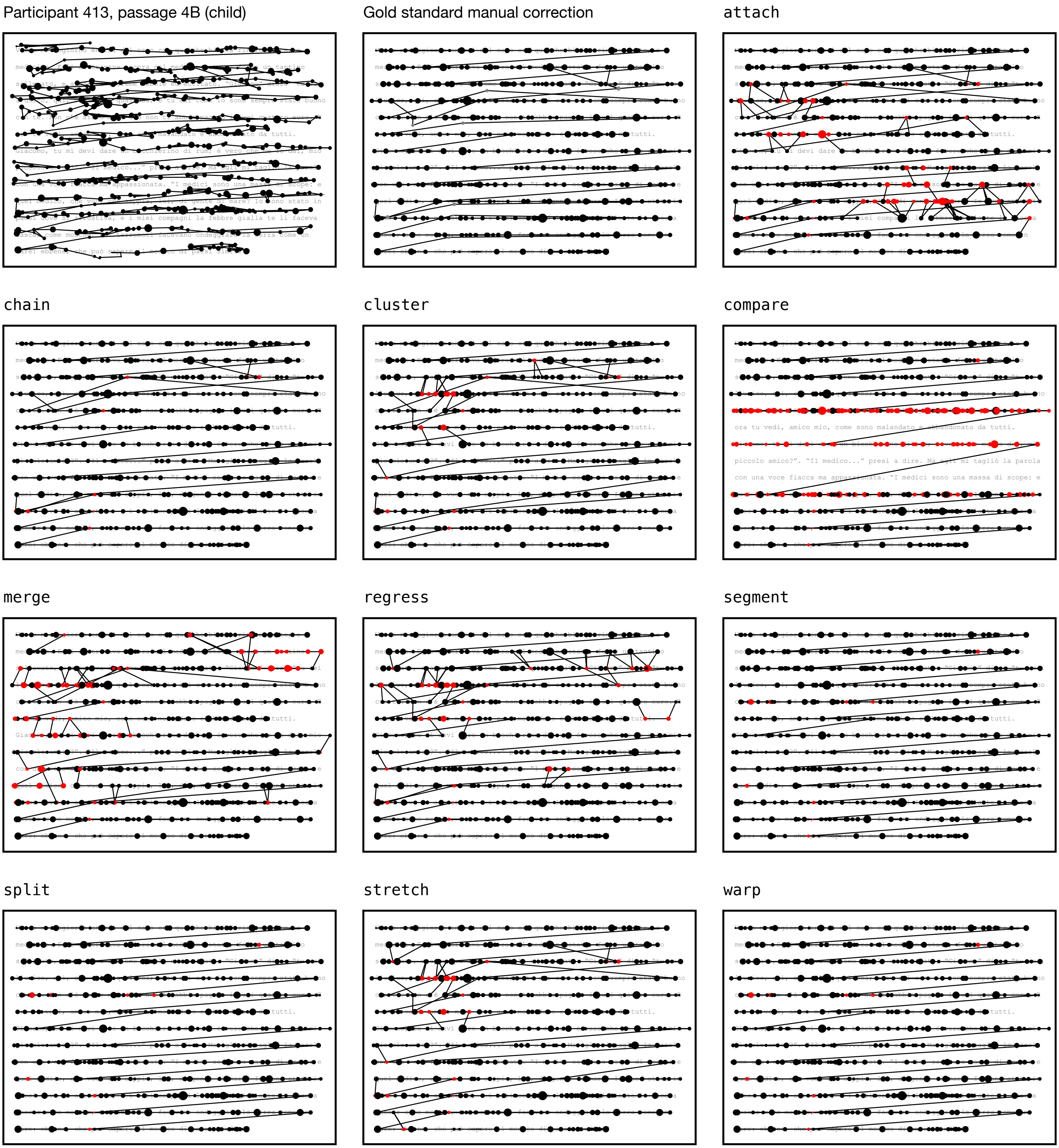
Participant 413, passage 4B (child)
Gold standard manual correction
attach
chain
cluster
compare
ora tu vedi, amico mio, come sono malandato e abbandonato da tutti.
piccolo amico?”. “Il medico...” presi a dire. Ma egli mi tagliò la parola
con una voce fiacca ma appassionata. “I medici sono una massa di scope: e
merge
regress
segment
split
stretch
warp

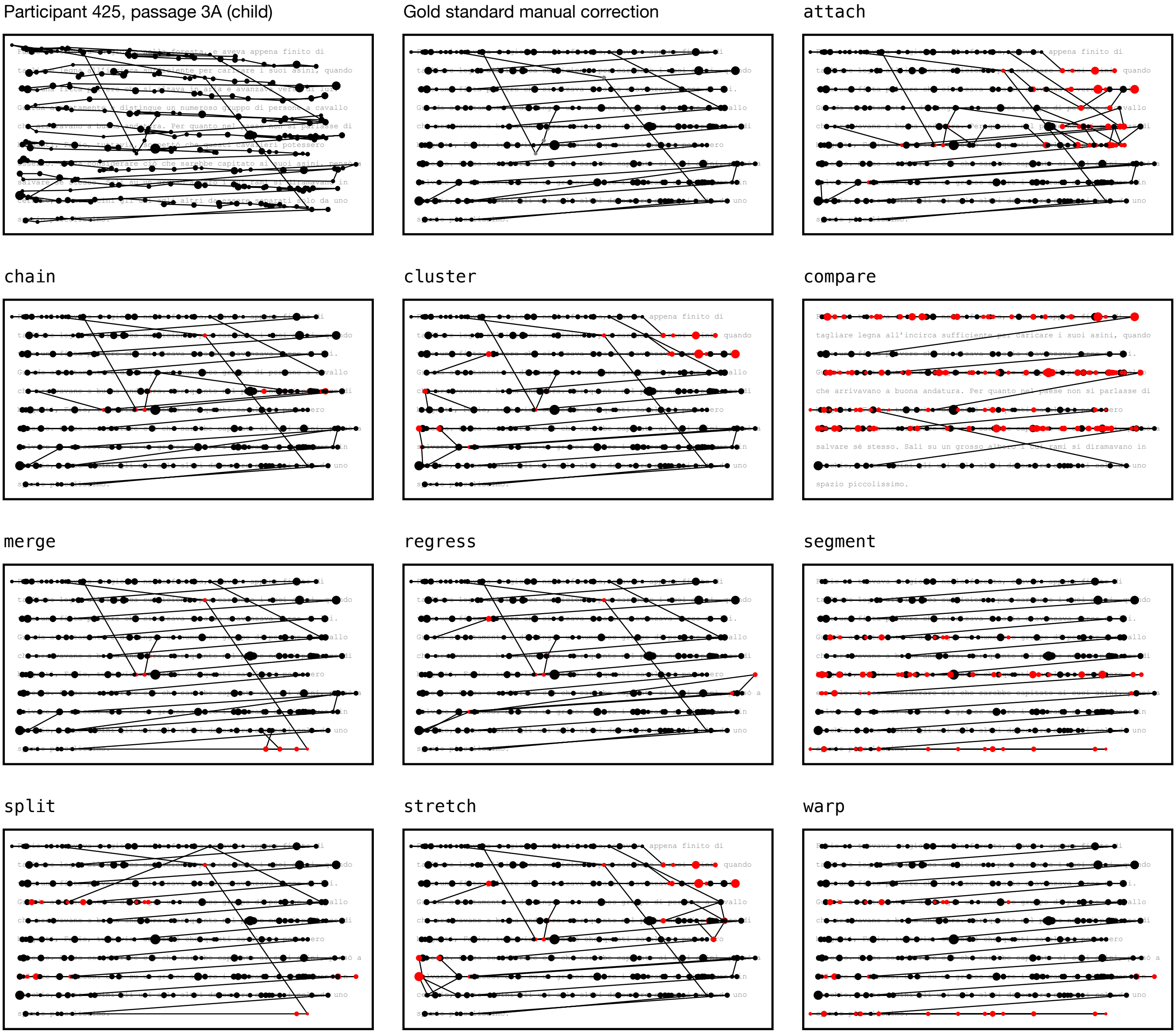
Participant 425, passage 3A (child)
Gold standard manual correction
attach
chain
cluster
compare
merge
regress
segment
split
stretch
warp

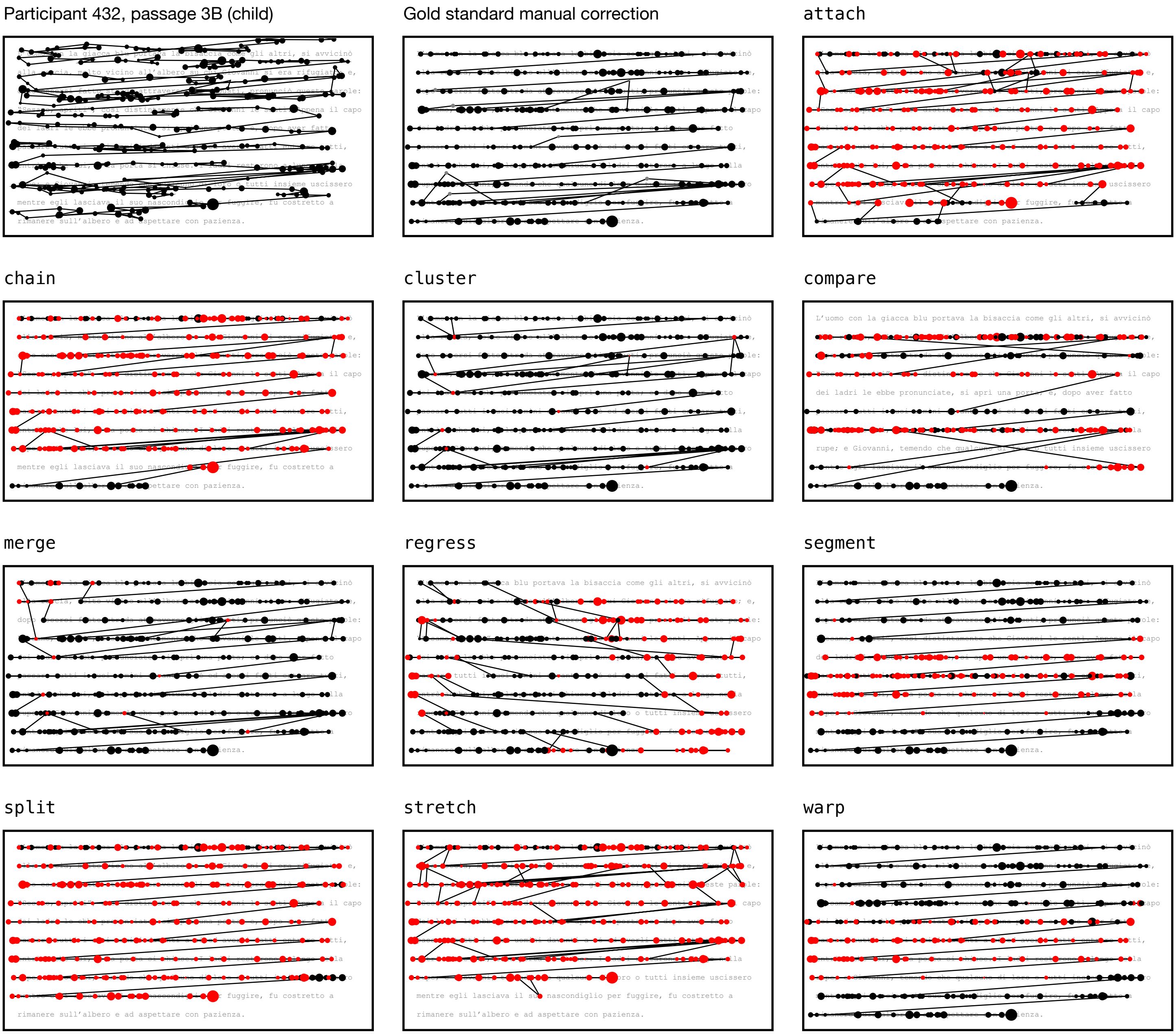
Participant 432, passage 3B (child)
Gold standard manual correction
attach
chain
cluster
compare
merge
regress
segment
split
stretch
warp

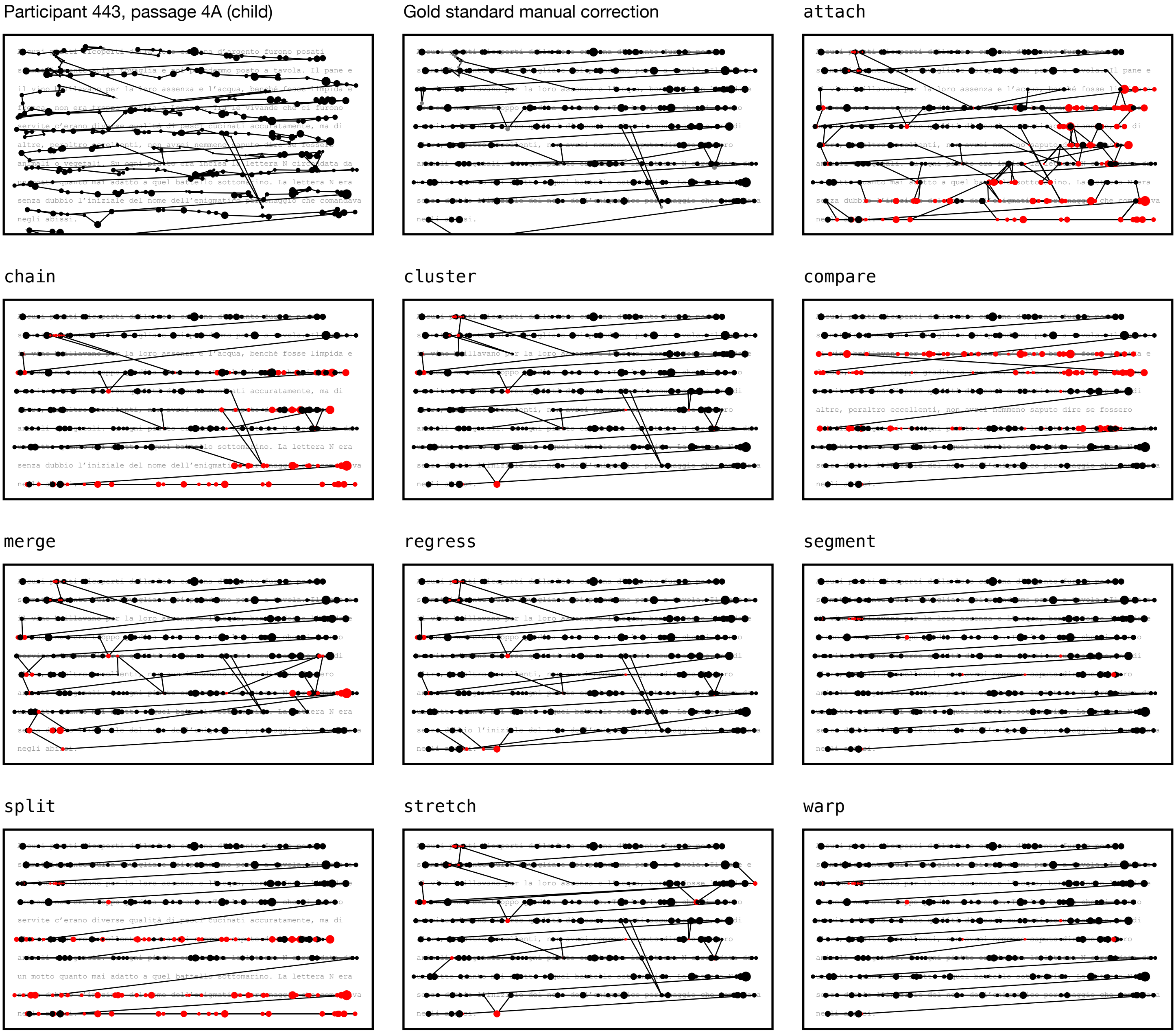
Participant 443, passage 4A (child)
Gold standard manual correction
attach
chain
cluster
compare
merge
regress
segment
split
stretch
warp
